# A Proposal of the Ur-RNAome

**DOI:** 10.3390/genes14122158

**Published:** 2023-11-29

**Authors:** Miryam Palacios-Pérez, Marco V. José

**Affiliations:** 1 Theoretical Biology Group, Instituto de Investigaciones Biomédicas, Universidad Nacional Autónoma de México, Ciudad de México 04510, Mexico; 2 Network of Researchers on the Chemical Emergence of Life (NoRCEL), Leeds LS7 3RB, UK; 3 NoRCEL’s Latin America Hub, 113 Philosophy Hall, University of California, Berkeley, CA 94720, USA

**Keywords:** RNA, RNY code, evolution of genetic code, proto-tRNA, LUCA

## Abstract

It is widely accepted that the earliest RNA molecules were folded into hairpins or mini-helixes. Herein, we depict the 2D and 3D conformations of those earliest RNA molecules with only RNY triplets, which Eigen proposed as the primeval genetic code. We selected 26 species (13 bacteria and 13 archaea). We found that the free energy of RNY hairpins was consistently lower than that of their corresponding shuffled controls. We found traces of the three ribosomal RNAs (16S, 23S, and 5S), tRNAs, 6S RNA, and the RNA moieties of RNase P and the signal recognition particle. Nevertheless, at this stage of evolution there was no genetic code (as seen in the absence of the peptidyl transferase centre and any vestiges of the anti-Shine–Dalgarno sequence). Interestingly, we detected the anticodons of both glycine (GCC) and threonine (GGU) in the hairpins of proto-tRNA.

## 1. Introduction

Before the emergence of catalytic proteins and DNA for heredity as separate molecules, RNA was the first biological molecule. Two of its characteristics, while prone to mistakes, allowed life to arise in a hypothetical “RNA world”: it could store information and act as a catalyst for processes like self-excision [1,2,3]. Test tube experiments have showed the various catalytic properties of RNA, reinforcing the idea that the first biochemical systems could have been entirely centred on that molecule [4].

Since RNA is the most versatile of all the biological macromolecules, and based on physicochemical theoretical works, RNA is thought to have originated the genetic code ~4.36 + 0.1 billion years ago [5,6,7,8,9]. Eigen and Schuster [10] glimpsed that the primeval genetic code (PGC) consisted of ribonucleotide chains following the pattern RNY, in which R means purines (A/G) and Y means pyrimidines (C/U), while N symbolises any of the nucleobases (A/C/G/U) in accordance with the parity rule R:Y. It was also shown that RNY is the main pattern in ribosomal RNA (rRNA) subunit 5S (5S rRNA) for more than 200 varied species [11], and that only primitive transfer RNA (tRNA) molecules with the RNY pattern are susceptible to being efficiently replicated, translated, and therefore amplified [12]. 

Early RNA genes could have been very short, reaching a maximum length of 50 to 100 nt, with most probably configured into mini-helixes whose strands must are stable and therefore equivalent to each other in topology and chemical composition [5,6,7,10,13,14,15,16,17,18,19]. These probably functioned as proto-tRNAs [20,21,22,23]. Indeed, these proto-tRNAs have been found to be concatenated to form all the rRNAs [24], including the peptidyl transferase centre (PTC) of the ribosome [25,26,27]; the concatamers can be translated into functional, modern-like proteins [28]. It has been proposed that the Ur-gene was composed of RNY sequences, while the RNA was folded in hairpins and mini-helixes resembling proto-tRNAs, which were able to bind prebiotic amino acids (aa) [5,6,7,10,29] that were also encoded by RNY triplets i.e., aa encoded by the aforementioned PGC. Actually, the mini-helix is thought to be the most ancient historical domain of tRNAs (3.6–3.7 BYA) [30].

We previously obtained the phenotype of amino acids and proteins corresponding to the evolution of genetic code [29,31], but RNA evolution was simply ignored. In this work, we determine the 2D and 3D structures of early RNA molecules based on the PGC. We find that those RNAs can indeed fold into short hairpins, and we even capture the anticodon loop of some of the earliest tRNA isoacceptors able to carry prebiotic aa.

## 2. Methods

We retrieved the RNAome from phylogenetically distant organisms, from which the triplets that did not belong to the early genetic code (RNY) were discarded. The sequences were then grouped according to type; then, each fragment was assembled into its original order in cases that the original gene had more than one fragment encoded by RNY triplets. To generate negative controls, the sequences were shuffled thrice. If more than one organism contained at least one RNA fragment encoded by RNY triplets, the fragments of each RNA type were arranged according to the original order in the gene; the RNAs were then aligned with each other to obtain a consensus sequence, and the corresponding logo sequences were generated. It is worth recalling that Ts were replaced by Us. Finally, we obtained the 2D and 3D structures of the RNAs encoded by RNY triplets. Each of the steps that we followed is detailed below, and a graphical flowchart can be found in Supplementary File S1.

### 2.1. Data Sources

From https://ftp.ncbi.nlm.nih.gov/genomes/refseq/ accessed on 4 October 2023, we obtained all the RNAs (*RNA*.fna) of 13 bacteria (bac.) [*Aquifex aeolicus* VF5 (NC_000918.1) → “AqfxV”, *Bacillus subtilis* 168 (NC_000964.3) → “Basub”, *Borreliella burgderfori* B31 (NC_001318.1) → “Bobur”, *Deinococcus radiodurans* R1 (NC_001263.1 y NC_001264.1) → “Derad”, *Escherichia coli* K12 MG1655 (NZ_CP025268.1) → “EcoK12”, *Mycoplasma genitalium* G37 (NC_000908.2) → “Mygen”, Ca. *Pelagibacter ubique* HTCC1062 (NC_007205.1) → “Peubi”, *Shewanella piezotolerans* WP3 (NC_011566.1) → “Shpz3”, *Streptococcus agalactiae* A909 (NC_007432.1) → “SagA”, *Synechococcus* CC9902 (NC_007513.1) → “SynCC”, *Thermotoga maritima* MSB8 (NC_000853.1) → “Thmar”, *Thermus aquaticus* Y51MC23 (NZ_CP010822.1) → “TaqY51”, *Thermus thermophilus* HB8 (NC_006461.1), → “Ther2”] and 13 archaea (arc.) [*Acidianus hospitalis* W1 (NC_015518.1) → “Aciho”, Ca. *Nitrosopumilus sediminis* AR2 (NC_018656.1) → “Nised”, *Haloarcula marismortui* ATCC 43049 (NC_006396.1 y NC_006397.1) → “Hamar”, *Haloferax volcanii* DS2 (NC_013967.1) → “Hxvol”, *Haloquadratum walsbyi* DSM 16790 (NC_008212.1) → “Haqwa”, *Korarchaeum cryptofilum* OPF8 (NC_010482.1) → “Kocry”, *Methanocaldococcus jannaschii* DSM_2661 (NC_000909.1) → “Mejan”, *Methanosarcina acetivorans* C2A (NC_003552.1) → “Macet”, *Pyrococcus furiosus* DSM 3638 (NC_003413.1) → “Pyfur”, *Sulfolobus acidocaldarius* DSM 639 (NC_007181.1) → “Sacid”, *Thermococcus gammatolerans* EJ3 (NC_012804.1) → “Thgam”, *Thermococcus sibiricus* MM739 (NC_012883.1) → “Thsib”, *Thermoplasma volcanium* GSS1 (NC_002689.2) → “Tmvol”].

### 2.2. Reconstruction of Sequences of Ancient RNAomes

To reconstruct the original arrangement of the RNAome, all RNAs were assembled one after the other, i.e., coding-wise (CW) with an ad hoc program, just as they are reported in the file *RNA*.fna, allowing a posterior alignment.

To generate an ad hoc filter for our arrangements, we also generated a random sequence from each assembled RNAome, shuffling the nucleotides thrice to eliminate the biological sense and information of the sequence. From each RNAome, and from its corresponding shuffled control, we discarded all triplets except those of the RNY type.

### 2.3. Grouping and Assembly of RNAs

Using BLASTn [32] as a standalone version, we used the biological RNAomes that we constructed (previously mentioned) by concatenating all the RNA sequences of each organism and their corresponding controls (the shuffled sequences) as queries. This allowed us to obtain the RNAs encoded by the PGC using the file *RNA*.fna* of every organism as the databases. Since RNY possesses only a quarter of the number of triplets as the SGC, we adjusted the parameters to allow as many outcomes as possible from those commonly used in BLAST searches, while preserving the maximum E-value at 10. To determine a cut-off value for the RNAs retrieved, numerical comparisons of the E-values of each of the biological RNAomes in RNY with their corresponding controls (those previously shuffled) were performed, thus setting the cut-offs for each organism.

The length of the RNA molecules was not selected beforehand but was the result of using a BLAST alignment for two sequences so that the fragments were retrieved as they were encoded by RNY triplets in the RNA molecules of each organism.

We grouped all the retrieved fragments according to the RNA molecule to which each one belongs; for instance, all fragments belonging to 5S rRNA were grouped together, all RNA fragments of A-type RNase P were grouped together, etc., and this was performed for each organism. Additionally, the tRNAs were sorted according to their cognate aa and the anticodon of each, which we identified using the programs ‘tRNA finder’ [33] and ‘tRNA scan’ [34]. Note that we provide the anticodon, and not the codon, of each aa.

### 2.4. MSAs of RNAs

Not all forms of RNA recovered have RNY triplet-encoded segments in more than one organism. In fact, only ribosomal RNAs (rRNAs) can be aligned with each other because several organisms contain more than one copy of the same gene, and the RNY-encoded portions are at similar positions. To align the small fragments encoded by RNY triplets of the ribosomal genes, we used the CHAOS-DIALIGN software (version 2.2.2) [35], as it works best with fragmentary sequences in local alignments. From the multiple sequence alignments (MSAs) generated, we obtained the consensus sequence using the UGENE suite [36].

### 2.5. Sequence Logos

We generated a graphical representation, in the form of sequence logos [37], of the MSAs of the RNA molecules encoded by RNY triplets.

### 2.6. Representation in 2D of the Recovered Fragments

We predicted the secondary structure of all our individual RNA sequences, or their consensuses, within the webserver RNAfold of the ViennaRNA suite [38], selecting the structure with the minimum free energy (MFE) under the Andronescu model, avoiding isolated base pairs, and leaving all other parameters the same. The 2D structures were visualised with the tool forna [39].

### 2.7. Representation in 3D of the Reconstructed Fragments

For RNA molecules encoded by RNY triplets, we adopted the Vienna format (dot-bracket notation) provided by the 2D-structure prediction program to construct de novo the corresponding 3D structure on the automated modelling server RNAcomposer (version 1.0) [40]. The structures were visualised using Chimera software (version 1.14) [41].

### 2.8. Negative Controls

To generate control sequences, we shuffled each of the RNY-encoded fragments (or their consensus sequences) thrice and obtained their thermodynamic descriptions and 2D and 3D structures, as was performed for the biological sequences.

## 3. Results and Analyses

We used the genomes of 26 organisms with different lifestyles (13 bacteria and 13 archaea) based upon the latest update of the tree of life (ToL), which places eukaryotes among the latter [42,43,44,45,46,47].

There are several challenges in modelling an RNA molecule de novo, and the difficulty increases as the length does by virtue of the fact that RNA folding depends on numerous parameters [48]. Accordingly, the folding of RNY-encoded fragments did not entail additional difficulties, as they are mostly short and self-complementary; however, this detail is particularly interesting because several authors have considered early RNAs to be folded like hairpins or mini-helixes.

Table S1T in Supplementary File S2 lists the MFE of the RNA fragments encoded by RNY triplets, as well as the corresponding controls (shuffled sequences). Notice that the MFEs of the majority of the negative controls are higher than the biological sequences from which they come, i.e., the biological structures are more stable than their controls. In some cases, the MFEs of the biological sequences and the negative controls are zero (or just slightly lower than the biological one), which indicates that the results are not artefactual.

In some organisms, the three rRNAs and nine tRNAs have well-defined portions encoded by RNY triplets. Moreover, the RNA moieties of some signal recognition particles (SRP-RNA), RNases P (RNA-P), and RNAs 6S, retain small antique portions. All figures not shown in the main text can be found in Supplementary File S3.

The 5′ end is always the first nucleotide in 2D structures; however, we placed each 3D RNA structure with the 5′ position towards the viewer and labelled both ends (5′ and 3′) in Supplementary File S3 (so as not to clutter the main text).

### 3.1. Ribosomal RNAs

The ribosome is a ribonucleoprotein lair formed by two subunits—a large ribosomal subunit (LSU) and a small ribosomal subunit (SSU)—in which, in turn, peptide growth is enabled entirely by RNA and the structural scaffold is provided by ribosomal proteins. The 16S rRNA couples with the messenger RNA (mRNA) to be translated into proteins according to the codons in it. The most critical portion of the translation is the peptidyl transferase centre (PTC), embedded in 23S rRNA; the PTC is formed by the A site and P site, while the important E site does not belong to the PTC. Finally, 5S rRNA keeps the tRNAs positioned at the A and P sites until translation finishes. Both 23S and 5S rRNAs belong to the LSU, while 16S rRNA belongs to the SSU [49,50,51,52,53].

**Figure 1:**
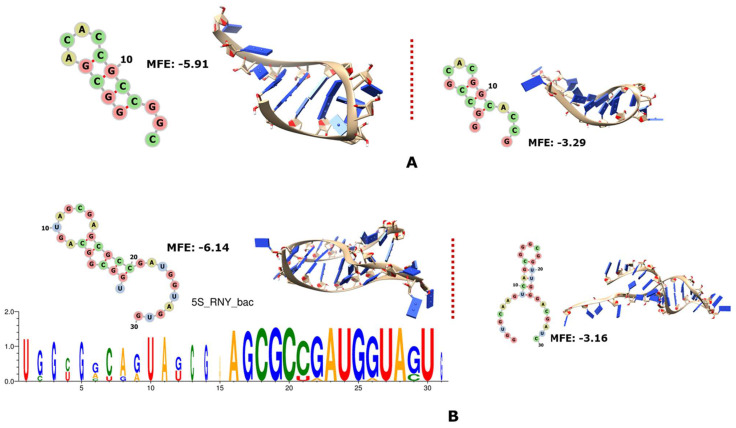
The 2D and 3D structures of RNY−encoded portions of 5S rRNA, as well as the logo sequences of the bacterial alignment; the MFE is also indicated in each case. On each panel, the structures and MFE values of the biological sequences are on the left side of the dotted line and the controls are on the right side. Archaea are shown in (**A**) and bacteria in (**B**).

**Figure 2:**
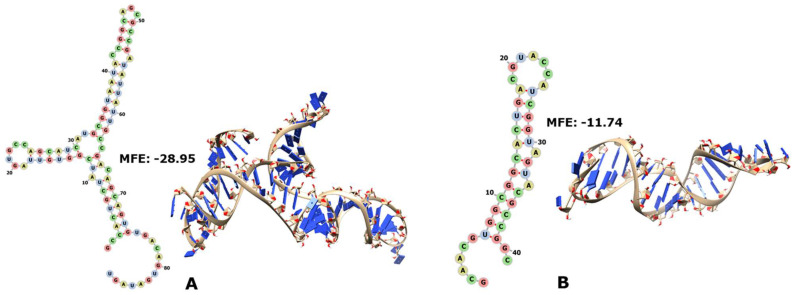
In (**A**), 2D and 3D structures of RNY−encoded portions of 16S rRNA from archaea; in (**B**), 2D and 3D structures of RNY-encoded portions of 16S rRNA from bacteria. The MFE is indicated in each case.

**Figure 3:**
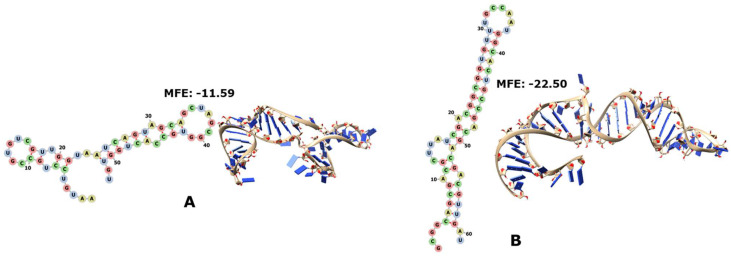
In (**A**), 2D and 3D structures of RNY−encoded portions of 23S rRNA from archaea; in (**B**), 2D and 3D structures of RNY−encoded portions of 23S rRNA from bacteria. The MFE is indicated in each case.

The three types of rRNA have portions encoded by RNY triplets. In some cases, only one organism has a recognisable sequence of this type, but for most of them the ribosomal RNAs from several organisms have PGC-encoded portions, so it makes sense to generate sequence alignments to obtain consensuses that can be modelled in 2D and 3D. In all cases, we can see that the biological sequence is more stable than its control (Figure 1, Figure 2 and Figure 3 here below and Supplementary File S2), and they tend to conform into short or complex helixes.

Only the 5S rRNA of the archaeon “Mejan” has a portion encoded by RNY triplets at the end of the molecule, and this fragment is folded like a hairpin (Figure 1A). On the other hand, the 5S rRNAs of many of our bacteria have RNY-encoded regions in the first quarter and also mostly in the third quarter of the molecule (Figure 1B). 

The portions encoded by RNY triplets in both 16S and 23S rRNAs are punctually dispersed throughout both types of sequences (Figures S2 and S3 in Supplementary File S3, all the corresponding logo sequences and their corresponding controls can be found).

As we can observe, the RNY-encoding of 16S rRNA conforms to a clearly defined region, with weak nucleotides (A and T/U) flanked by strong nucleotides (G and C) in the middle of the sequence in both archaea (Figure S2A in Supplementary File S3) and bacteria (Figure S2B in Supplementary File S3); additionally, the 3′ end in bacteria is slightly less defined than in archaea.

On the other hand, RNY triplets recovered only a tiny fragment (60 nt) of 23S rRNA, with less conservation in the archaeal (Figure 3A) than in the bacterial (Figure 3B) reconstructions.

### 3.2. RNase P, SRP, and 6S

**Figure 4:**
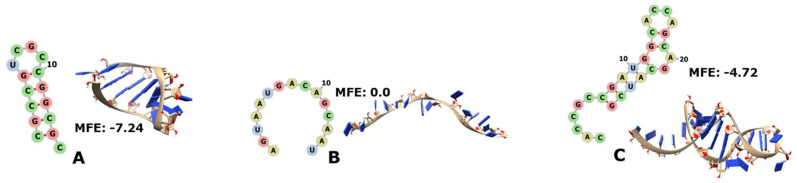
The 2D and 3D structures of RNY−encoded portions of the RNA moiety of RNase P (RNA-P). In (**A**), the archaeon “Hxvol”; in (**B**), the bacterium “Mygen”; in (**C**), the two sequences from bacterium “SynCC”. The corresponding controls are in Supplementary File S2.

**Figure 5:**
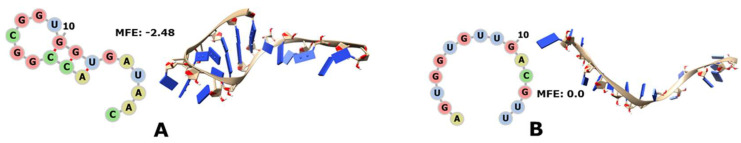
The 2D and 3D structures of RNY−encoded portions of the RNA moiety of SRP. In (**A**), the archaeon “Kocry”; in (**B**), the bacterium “Basub”. The corresponding controls are in Supplementary File S2.

**Figure 6:**
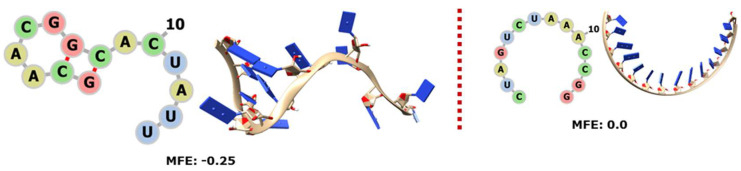
MFE values and 2D and 3D structures of the RNY−encoded portion of 6S RNA of the bacterium “Basub” and its corresponding control; the biological sequence is on the left side of the dotted line and its shuffling is on the right side.

The RNA moieties of some types of RNase P (Figure 4), some types of SRPs (Figure 5), and one 6S RNA (Figure 6), have at least one portion encoded by RNY triplets. Each of these fragments is present in only one, although not the same, of the 26 organisms selected here.

RNase P is a ubiquitous ribonucleoprotein that catalyses the maturation of tRNAs by removing their extraneous 5′ sequences. All species require the RNA moiety of the RNase P (RNA-P), but whereas in bacteria and archaea the protein portion is totally or just marginally dispensable, respectively, eukaryotes cannot survive without the proteins of their RNase P [54,55,56]. The RNA-Ps of the archaeon “Hxvol” (Figure 4A) and of the bacterium “Mygen” (Figure 4B) each have one portion encoded by RNY triplets. The archaeal RNA-P fragment is more stable than its control, whereas the MFE of the second bacterial RNA-P fragment is zero, as is that of its shuffled control (Supplementary File S2). On other hand, the RNA-P of the bacterium “SynCC” has two fragments encoded by the PGC. In Figure 4C, we observe the concatenation of both fragments in the same order as they appear in the original RNA molecule, and this construct is more stable than its corresponding control. When each fragment is modelled individually (Supplementary File S2), we see that the first one is more stable than its control, and that the stability of the control of the second fragment is just slightly higher than that of the biological sequence. All those RNA-P fragments encoded by RNY triplets, whose MFE is different from zero, tend to form mini-helixes or hairpin-like structures.

The signal recognition particle (SRP) is a widely distributed GTP-dependent ribonucleoprotein that helps direct the protein synthesis towards the membrane when needed. This SRP-RNA serves as the scaffold on which all its proteins will be assembled. SRP has so far been described in two variants in bacteria and one in archaea, as well as many more versions in eukaryotes. The SRP-RNA has several self-complementary regions that can fold into a few or many helixes [57,58]. The RNY triplets partially encode the RNA moieties of the SRP of the archaeon “Kocry” (Figure 5A) and of the bacterium “Basub” (Figure 5B). The archaeal SRP-RNA is much more stable than its control (Figure S5A in Supplementary File S3); the bacterial SRP-RNA and its control (Figure S5B in Supplementary File S3) have an MFE of zero, although the entropy is slightly higher than in the shuffled sequence.

The 6S RNA molecule is a widespread small global regulator of bacterial transcription that mimics B-form DNA and then binds to the active site of RNA polymerase (RNApol), thus blocking the transcription and enabling the release of the enzyme RNApol. It folds into a single, long self-complementary structure with some internal loops along the length of the molecule [59,60,61,62,63,64]. The molecule 6S RNA of the bacterium “Basub” is the only one of its kind with a portion encoded by RNY triplets (Figure 6), and it has a slight hairpin-like folding; although the paired bases are too few to achieve this, their entropy is certainly lower than that of its corresponding control.

### 3.3. tRNAs

**Figure 7:**
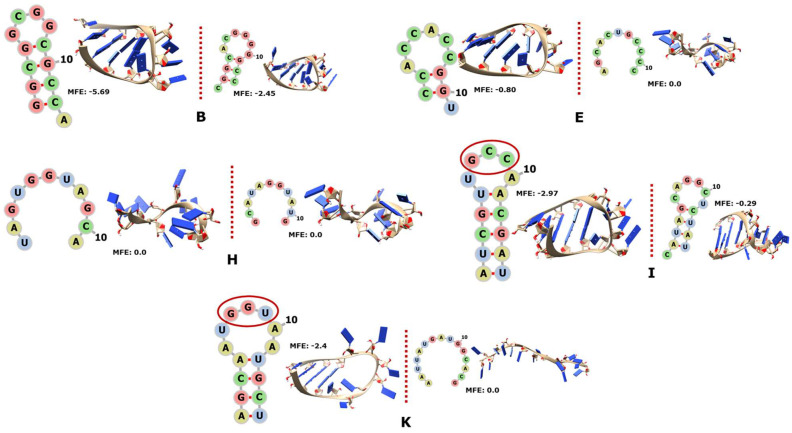
The 2D and 3D structures of RNY−encoded portions of some tRNAs. In (**B**), Asn-5′GUU from “Thgam”; in (**E**), Cys-5′GCA from “Derad”; in (**H**), Gly-5′UCC from “Derad”; in (**I**), Gly-5′**GCC** from “Peubi” with the anticodon circled in red; in (**K**), Thr-5′**GGU** from “SagA” with the anticodon circled in red. The complete catalogue is in Supplementary File S2.

The tRNAs are molecules ranging from 76 to about 90 nt in length that fold into a 2D cloverleaf or a 3D L-shape. The tRNAs serve as the physical adaptors between the genetic code “read” by the anticodon in the middle of the molecule and the phenotype in the form of the corresponding aa charged in the distal 3′ portion [65,66,67]. We found 12 tRNAs with one portion each encoded by RNY triplets (Figure 7, with only a few examples; the complete catalogue 7A to 7L is in Supplementary File S2); five of them can fold into small hairpins (sort of helix-like), while the other tRNA fragments remain unfolded.

In most cases, the biological sequences are more stable than their corresponding controls, such as the tRNA of the archaea “Haqwa” for Gln-UUG (Figure S7A in Supplementary File S3 or of “Thgam” for Asn-GUU (Figure S7B below). Some other cases are only slightly more stable than the controls, such as the tRNA of the bacterium “Derad” for Cys-GCA (Figure S7E in Supplementary File S3). On a few occasions, the MFE of the biological sequence is zero, as is that of its control sequence, as in the case of bacteria “Bobur” for Gln-UUG (Figure S7D in Supplementary File S3) or “Derad” for Gly-UCC (Figure S7H below).

Remarkably, three of the RNY-encoded fragments capture the anticodons of their corresponding tRNAs. To wit, Gly-tRNA_**GCC** of the bacterium “Peubi” (Figure S7I below) and Thr-tRNA_**GGU** of the bacterium “SagA” (Figure S7K below) totally capture the anticodons (letters underlined in the text and circled in red in the corresponding figures) that are located just in the middle of the fragments and therefore at the loop of hairpin. Moreover, the bases of the anticodons point outwards as in the full tRNA molecules; each of the activating amino acids of these anticodons is also encoded by the PGC (Gly and Thr). This contrasts with Gly-tRNA_UCC of the bacterium “Derad” (Figure S7H below) because, even if Gly were encoded very early, the PGC would not include the anticodon UCC. On the other hand, the fragment of tRNA-Gln_C**UG** of the bacterium “Derad” (Figure S7F in Supplementary File S3) encoded by RNY triplets remains unfolded, as we mentioned earlier; the contrast relies on the fact that the anticodon is only partially included (only the letters underlined) in the fragment encoded by the PGC and that glutamine is not encoded by RNY triplets.

Finally, in several cases the control sequence is even more stable than the biological one but has a minor difference, as with the tRNA of the bacterium “SagA” for Asn-GUU (Figure S7H below) or “Thmar” for Phe-GAA (Figure S7L in Supplementary File S3).

## 4. Discussion

**Figure 8:**
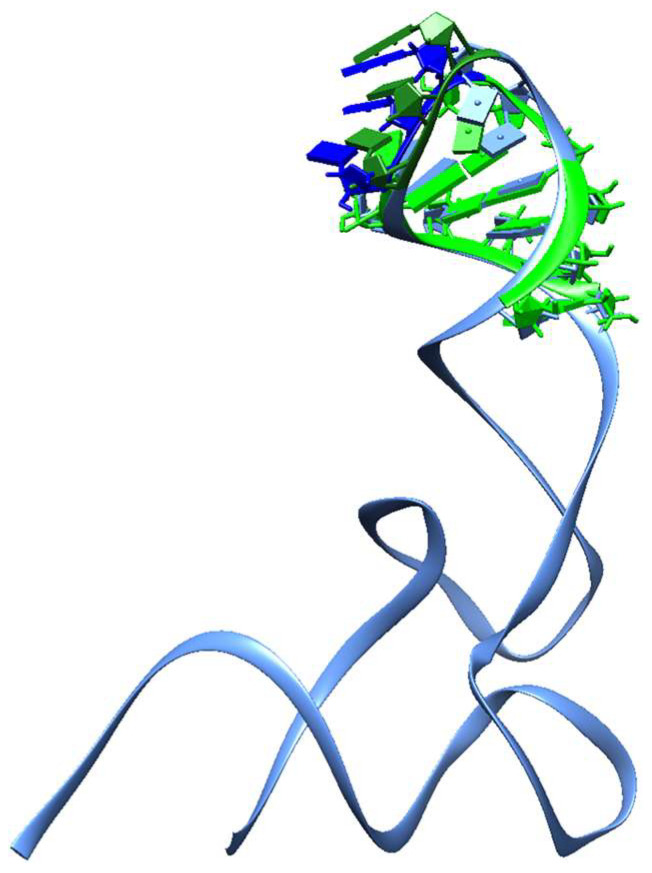
Structural comparison among the predicted structure of the sequence encoded by RNY triplets of Gly-tRNA_GCC and its modern structure (PDB ID 4mgn). The structure encoded by the PGC is in light green, and its anticodon is in forest green; the crystallographic structure is in mid-blue, and its anticodon is in deep blue.

Though not all organisms have RNA with RNY-encoded portions, and although such fragments are very small and can barely be aligned, it is noteworthy that all the RNA molecules directly involved in the modern translation process withhold a snippet encoded by the PGC. We found that such RNY triplet-encoded RNA snippets can fold into small hairpins shorter than the size proposed for early functional genes (or even for proto-tRNAs) able to shape all the other biomolecules [24], suggesting an earlier stage in the evolution [68] that probably constituted the beginnings of these RNA molecules. Moreover, when we compare the predicted structure of the sequence encoded by RNY triplets of Gly-tRNA_GCC with its modern structure (PDB ID 4mgn), we observe that not only the whole structures, but even the anticodon bases of both, are in almost the same outward positions (Figure 8). In contrast, in the case of the Gly-tRNA_UCC fragment, the anticodon is not retrieved, a fact already reckoned with in 1981 [20,21] regarding a differential emergence of tRNA isoacceptors.

It is strikingly important to have discovered that the anticodon stem of tRNA-Gly_GCC is purely encoded by RNY triplets because the whole tRNA cloverleaf may possibly have been formed via the ligation of proto-tRNA mini-helixes, 3–31 nt in length (one of which encoded for glycine_GCC [69]), which resemble some of the other small RNA molecules found to be encoded using RNY triplets. Without going any further, any modern tRNA could have its origin in mini-helixes [29,69,70,71] that could be replicated themselves [72], combining the operational code in an ancient anticodon helix with the informational code in an early acceptor helix [19], prior to the appearance of contemporary tRNA specificities, and quite before the three domains of life diverge [73].

Life most probably originated when proteins began to be translated, for which a well-established PTC (as well as the respective anti-Shine–Dalgarno sequence) is the sine qua non [27,74,75]; however, we did not find any of them encoded by RNY triplets, which places our work in the realm of the protobiotic stage, and the small RNA hairpins encoded by RNY as the primordial seeds that eventually grew and ligated to each other to form more recognisable modern RNA molecules.

RNA could then have polymerised and randomly generated short ribonucleic chains in which the RNY pattern gradually began to prevail, as if it was a quasi-species [6,58] that evolved through cooperative interaction via cyclic coupling, i.e., hypercycles. Those RNA short sequences and their limited diversity supported prebiotic, autocatalytic reproduction by means of hypercycles [5,10,15,16,17,18,21]. Lastly, it is safe to assume that the Ur-RNA proposed here, encoded by the PGC, emerged before the so-called “First Universal Common Ancestor” (FUCA), because the PTC cannot be found encoded by RNY triplets [69,70].

RNA evolved as one of the first phenotypic biomolecules and the primordial genotypic biomolecule, and the PGC of such RNAs followed the pattern RNY. The modern translation molecules have their origins from short RNA hairpins formed by triplets pertaining to the PGC. All the small hairpins here described possibly constituted the beginnings of the corresponding modern RNA molecules and were probably part of a larger pool of RNA molecules that served as the seeds of more complex molecules. The lengths of our RNA sequences (20–30 nt) are far from the error catastrophe limit [5], and the Ur-gene is proposed [20,21] to have a length between 50 and 100 nt.

Speaking of contemporary issues, synthetic genetic codes can be designed to generate new proteins, and it is known that mutations of tRNA are associated with several diseases. For instance, cellular and mitochondrial tRNA overexpression and mutation relate to a wide range of human diseases [76,77,78], such as breast cancer [79] and neuro-gastrointestinal encephalopathy [80].

The results presented here provide astounding evidence that our approach can detect molecular structures from the protobiotic stage >3.7 billion years ago with surprising confidence.

## Data Availability

Data are contained within the article and Supplementary Materials.

## Supplementary Materials

The following supporting information can be downloaded at: https://www.mdpi.com/article/10.3390/genes14122158/s1. File S1 contains the graphical flowchart of the Methodology. File S2 contains a table (S1T) of the MFE of each of the structures in this work and their controls. File S3 contains all the RNA sequence logos, and the 2D and 3D structures of each of the RNAs declared in this work.

## References

[1] Kruger K., Grabowski P.J., Zaug A.J., Sands J., Gottschling D.E., Cech T.R. (1982). Self-Splicing RNA: Autoexcision and Autocyclization of the Ribosomal RNA Intervening Sequence of Tetrahymena. Cell.

[2] Guerrier-Takada C., Gardiner K., Marsh T., Pace N., Altman S. (1983). The RNA Moiety of Ribonuclease P Is the Catalytic Subunit of the Enzyme. Cell.

[3] Gilbert W. (1986). Origin of Life: The RNA World. Nature.

[4] Brown T.A. (2002). Genomes.

[5] Eigen M., Gardiner W., Schuster P., Winkler-Oswatitsch R. (1981). The Origin of Genetic Information. Sci. Am..

[6] Wächtershäuser G. (2014). The Place of RNA in the Origin and Early Evolution of the Genetic Machinery. Life.

[7] Chatterjee S., Yadav S. (2019). The Origin of Prebiotic Information System in the Peptide/RNA World: A Simulation Model of the Evolution of Translation and the Genetic Code. Life.

[8] Benner S.A., Bell E.A., Biondi E., Brasser R., Carell T., Kim H.-J., Mojzsis S.J., Omran A., Pasek M.A., Trail D. (2020). When Did Life Likely Emerge on Earth in an RNA-First Process?. ChemSystemsChem.

[9] Lehman N. (2015). The RNA World: 4,000,000,050 Years Old. Life.

[10] Eigen M., Schuster P. (1978). The Hypercycle—A Principle of Natural Self-Organization Part C: The Realistic Hypercycle. Naturwissenschaften.

[11] Eigen M., Lindemann B., Winkler-Oswatitsch R., Clarke C.H. (1985). Pattern Analysis of 5S rRNA. Proc. Natl. Acad. Sci. USA.

[12] Lehmann J. (2002). Amplification of the Sequences Displaying the Pattern RNY in the RNA World: The Translation → Translation/Replication Hypothesis. J. Theor. Biol..

[13] Eigen M., Winkler-Oswatitsch R. (1981). Transfer-RNA: The Early Adaptor. Naturwissenschaften.

[14] Eigen M., Winkler-Oswatitsch R. (1981). Transfer-RNA, an Early Gene?. Naturwissenschaften.

[15] Delaye L., Lazcano A. (2005). Prebiological Evolution and the Physics of the Origin of Life. Phys. Life Rev..

[16] Kun Á., Szilágyi A., Könnyű B., Boza G., Zachar I., Szathmáry E. (2015). The Dynamics of the RNA World: Insights and Challenges. Ann. N. Y. Acad. Sci..

[17] Szilágyi A., Zachar I., Scheuring I., Kun Á., Könnyű B., Czárán T. (2017). Ecology and Evolution in the RNA World Dynamics and Stability of Prebiotic Replicator Systems. Life.

[18] Szilágyi A., Könnyű B., Czárán T. (2020). Dynamics and Stability in Prebiotic Information Integration: An RNA World Model from First Principles. Sci. Rep..

[19] Schimmel P., de Pouplana L.R. (1995). Transfer RNA: From Minihelix to Genetic Code. Cell.

[20] Ribas de Pouplana L., Buechter D., Sardesai N.Y., Schimmel P. (1998). Functional Analysis of Peptide Motif for RNA Microhelix Binding Suggests New Family of RNA-Binding Domains. EMBO J..

[21] Mizuuchi R., Lehman N. (2019). Limited Sequence Diversity Within a Population Supports Prebiotic RNA Reproduction. Life.

[22] Giulio M.D. (1992). On the Origin of the Transfer RNA Molecule. J. Theor. Biol..

[23] Dick T.P., Schamel W.W.A. (1995). Molecular Evolution of Transfer RNA from Two Precursor Hairpins: Implications for the Origin of Protein Synthesis. J. Mol. Evol..

[24] de Farias S.T., José M.V. (2020). Transfer RNA: The Molecular Demiurge in the Origin of Biological Systems. Prog. Biophys. Mol. Biol..

[25] Farias S.T., Rêgo T.G., José M.V. (2014). Origin and Evolution of the Peptidyl Transferase Center from Proto-tRNAs. FEBS Open Bio.

[26] Torres de Farias S., Gaudêncio Rêgo T., José M.V. (2017). Peptidyl Transferase Center and the Emergence of the Translation System. Life.

[27] Prosdocimi F., Zamudio G.S., Palacios-Pérez M., Torres de Farias S., José M.V. (2020). The Ancient History of Peptidyl Transferase Center Formation as Told by Conservation and Information Analyses. Life.

[28] de Farias S.T., do Rêgo T.G., José M.V. (2014). Evolution of Transfer RNA and the Origin of the Translation System. Front. Genet..

[29] Palacios-Pérez M., Andrade-Díaz F., José M.V. (2018). A Proposal of the Ur-Proteome. Orig. Life Evol. Biosph..

[30] Maizels N., Weiner A.M. (1994). Phylogeny from Function: Evidence from the Molecular Fossil Record That tRNA Originated in Replication, Not Translation. Proc. Natl. Acad. Sci. USA.

[31] José M.V., Zamudio G.S., Palacios-Pérez M., Bobadilla J.R., de Farías S.T. (2015). Symmetrical and Thermodynamic Properties of Phenotypic Graphs of Amino Acids Encoded by the Primeval RNY Code. Orig. Life Evol. Biosph..

[32] Altschul S.F., Gish W., Miller W., Myers E.W., Lipman D.J. (1990). Basic Local Alignment Search Tool. J. Mol. Biol..

[33] Kinouchi M., Kurokawa K. (2006). tRNAfinder: A Software System to Find All tRNA Genes in the DNA Sequence Based on the Cloverleaf Secondary Structure. J. Comput. Aided Chem..

[34] Lowe T.M., Chan P.P. (2016). tRNAscan-SE On-Line: Integrating Search and Context for Analysis of Transfer RNA Genes. Nucleic Acids Res..

[35] Brudno M., Steinkamp R., Morgenstern B. (2004). The CHAOS/DIALIGN WWW Server for Multiple Alignment of Genomic Sequences. Nucleic Acids Res..

[36] Okonechnikov K., Golosova O., Fursov M. (2012). Unipro UGENE: A Unified Bioinformatics Toolkit. Bioinformatics.

[37] Crooks G.E., Hon G., Chandonia J.-M., Brenner S.E. (2004). WebLogo: A Sequence Logo Generator. Genome Res..

[38] Gruber A.R., Lorenz R., Bernhart S.H., Neuböck R., Hofacker I.L. (2008). The Vienna RNA Websuite. Nucleic Acids Res..

[39] Kerpedjiev P., Hammer S., Hofacker I.L. (2015). Forna (Force-Directed RNA): Simple and Effective Online RNA Secondary Structure Diagrams. Bioinformatics.

[40] Popenda M., Szachniuk M., Antczak M., Purzycka K.J., Lukasiak P., Bartol N., Blazewicz J., Adamiak R.W. (2012). Automated 3D Structure Composition for Large RNAs. Nucleic Acids Res..

[41] Pettersen E.F., Goddard T.D., Huang C.C., Couch G.S., Greenblatt D.M., Meng E.C., Ferrin T.E. (2004). UCSF Chimera—A Visualization System for Exploratory Research and Analysis. J. Comput. Chem..

[42] Williams T.A., Foster P.G., Nye T.M.W., Cox C.J., Embley T.M. (2012). A Congruent Phylogenomic Signal Places Eukaryotes within the Archaea. Proc. R. Soc. B Biol. Sci..

[43] Raymann K., Brochier-Armanet C., Gribaldo S. (2015). The Two-Domain Tree of Life Is Linked to a New Root for the Archaea. Proc. Natl. Acad. Sci. USA.

[44] Spang A., Saw J.H., Jørgensen S.L., Zaremba-Niedzwiedzka K., Martijn J., Lind A.E., van Eijk R., Schleper C., Guy L., Ettema T.J.G. (2015). Complex Archaea That Bridge the Gap between Prokaryotes and Eukaryotes. Nature.

[45] Spang A., Ettema T.J.G. (2016). Microbial Diversity: The Tree of Life Comes of Age. Nat. Microbiol..

[46] Cornish-Bowden A., Cárdenas M.L. (2017). Life before LUCA. J. Theor. Biol..

[47] Imachi H., Nobu M.K., Nakahara N., Morono Y., Ogawara M., Takaki Y., Takano Y., Uematsu K., Ikuta T., Ito M. (2020). Isolation of an Archaeon at the Prokaryote–Eukaryote Interface. Nature.

[48] Mustoe A.M., Brooks C.L., Al-Hashimi H.M. (2014). Hierarchy of RNA Functional Dynamics. Annu. Rev. Biochem..

[49] Moore P.B., Steitz T.A. (2002). The Involvement of RNA in Ribosome Function. Nature.

[50] Sievers A., Beringer M., Rodnina M.V., Wolfenden R. (2004). The Ribosome as an Entropy Trap. Proc. Natl. Acad. Sci. USA.

[51] Wallin G., Åqvist J. (2010). The Transition State for Peptide Bond Formation Reveals the Ribosome as a Water Trap. Proc. Natl. Acad. Sci. USA.

[52] Jüttner M., Ferreira-Cerca S., Entian K.-D. (2022). A Comparative Perspective on Ribosome Biogenesis: Unity and Diversity Across the Tree of Life. Ribosome Biogenesis: Methods and Protocols.

[53] Wang Q., Su H. (2022). A Tale of Water Molecules in the Ribosomal Peptidyl Transferase Reaction. Biochemistry.

[54] Jarrous N. (2017). Roles of RNase P and Its Subunits. Trends Genet..

[55] Daniels C.J., Lai L.B., Chen T.-H., Gopalan V. (2019). Both Kinds of RNase P in All Domains of Life: Surprises Galore. RNA.

[56] Di Giulio M. (2022). The RNase P, LUCA, the Ancestors of the Life Domains, the Progenote, and the Tree of Life. Biosystems.

[57] Larsen N., Zwieb C. (1991). SRP-RNA Sequence Alignment and Secondary Structure. Nucleic Acids Res..

[58] Rosenblad M.A., Larsen N., Samuelsson T., Zwieb C. (2009). Kinship in the SRP RNA Family. RNA Biol..

[59] Barrick J.E., Sudarsan N., Weinberg Z., Ruzzo W.L., Breaker R.R. (2005). 6S RNA Is a Widespread Regulator of Eubacterial RNA Polymerase That Resembles an Open Promoter. RNA.

[60] Wassarman K.M. (2007). 6S RNA: A Small RNA Regulator of Transcription. Curr. Opin. Microbiol..

[61] Cavanagh A.T., Wassarman K.M. (2014). 6S RNA, a Global Regulator of Transcription in Escherichia Coli, Bacillus Subtilis, and Beyond. Annu. Rev. Microbiol..

[62] Burenina O.Y., Elkina D.A., Hartmann R.K., Oretskaya T.S., Kubareva E.A. (2015). Small Noncoding 6S RNAs of Bacteria. Biochemistry.

[63] Chen J., Wassarman K.M., Feng S., Leon K., Feklistov A., Winkelman J.T., Li Z., Walz T., Campbell E.A., Darst S.A. (2017). 6S RNA Mimics B-Form DNA to Regulate Escherichia Coli RNA Polymerase. Mol. Cell.

[64] Wassarman K.M. (2018). 6S RNA, A Global Regulator of Transcription. Microbiol. Spectr..

[65] Berg J.M., Tymoczko J.L., Stryer L. (2002). Biochemistry.

[66] Alberts B., Johnson A., Lewis J., Raff M., Roberts K., Walter P. (2002). Molecular Biology of the Cell.

[67] Berg M.D., Brandl C.J. (2021). Transfer RNAs: Diversity in Form and Function. RNA Biol..

[68] Hyeon C., Thirumalai D. (2012). Chain Length Determines the Folding Rates of RNA. Biophys. J..

[69] Root-Bernstein R., Kim Y., Sanjay A., Burton Z.F. (2016). tRNA Evolution from the Proto-tRNA Minihelix World. Transcription.

[70] Rodin S., Rodin A., Ohno S. (1996). The Presence of Codon-Anticodon Pairs in the Acceptor Stem of tRNAs. Proc. Natl. Acad. Sci. USA.

[71] Tamura K., Schimmel P. (2004). Chiral-Selective Aminoacylation of an RNA Minihelix. Science.

[72] Guo X., Su M. (2023). The Origin of Translation: Bridging the Nucleotides and Peptides. Int. J. Mol. Sci..

[73] Widmann J., Di Giulio M., Yarus M., Knight R. (2005). tRNA Creation by Hairpin Duplication. J. Mol. Evol..

[74] Prosdocimi F., de Farias S.T. (2022). Entering the Labyrinth: A Hypothesis about the Emergence of Metabolism from Protobiotic Routes. Biosystems.

[75] Prosdocimi F., de Farias S.T. (2023). Origin of Life: Drawing the Big Picture. Prog. Biophys. Mol. Biol..

[76] Abbott J.A., Francklyn C.S., Robey-Bond S.M. (2014). Transfer RNA and Human Disease. Front. Genet..

[77] Brandon M.C., Lott M.T., Nguyen K.C., Spolim S., Navathe S.B., Baldi P., Wallace D.C. (2005). MITOMAP: A Human Mitochondrial Genome Database—2004 Update. Nucleic Acids Res..

[78] Yarham J.W., Elson J.L., Blakely E.L., McFarland R., Taylor R.W. (2010). Mitochondrial tRNA Mutations and Disease. WIREs RNA.

[79] Pavon-Eternod M., Gomes S., Geslain R., Dai Q., Rosner M.R., Pan T. (2009). tRNA Over-Expression in Breast Cancer and Functional Consequences. Nucleic Acids Res..

[80] Glatz C., D’Aco K., Smith S., Sondheimer N. (2011). Mutation in the Mitochondrial tRNAVal Causes Mitochondrial Encephalopathy, Lactic Acidosis and Stroke-like Episodes. Mitochondrion.

