# Supplementary material for: A Proposal of the Ur-RNAome
Source: Genes (Basel). 2023 Nov 29;14(12):2158. doi: 10.3390/genes14122158 (PMC10743229; doi:10.3390/genes14122158)

S1 for **A proposal of the Ur-RNAome**

Miryam Palacios-Pérez [a,b,c,◎] & Marco V. José [a,b,*]

[a] *Theoretical Biology Group, Instituto de Investigaciones Biomédicas, Universidad Nacional Autónoma de México, Ciudad de México, México*

[b] *Member of the Network of Researchers on the Chemical Emergence of Life (NoRCEL)*

[c] *Second vice-president of NoRCEL and Head of NoRCEL's Latin America Hub*

Corresponding authors E-mail addresses:

◎MPP – mir.pape@iibiomedicas.uunam.mx ; *MVJ – marcojose@iibiomedicas.unam.mx

S2. Graphical flowchart of the Methodology

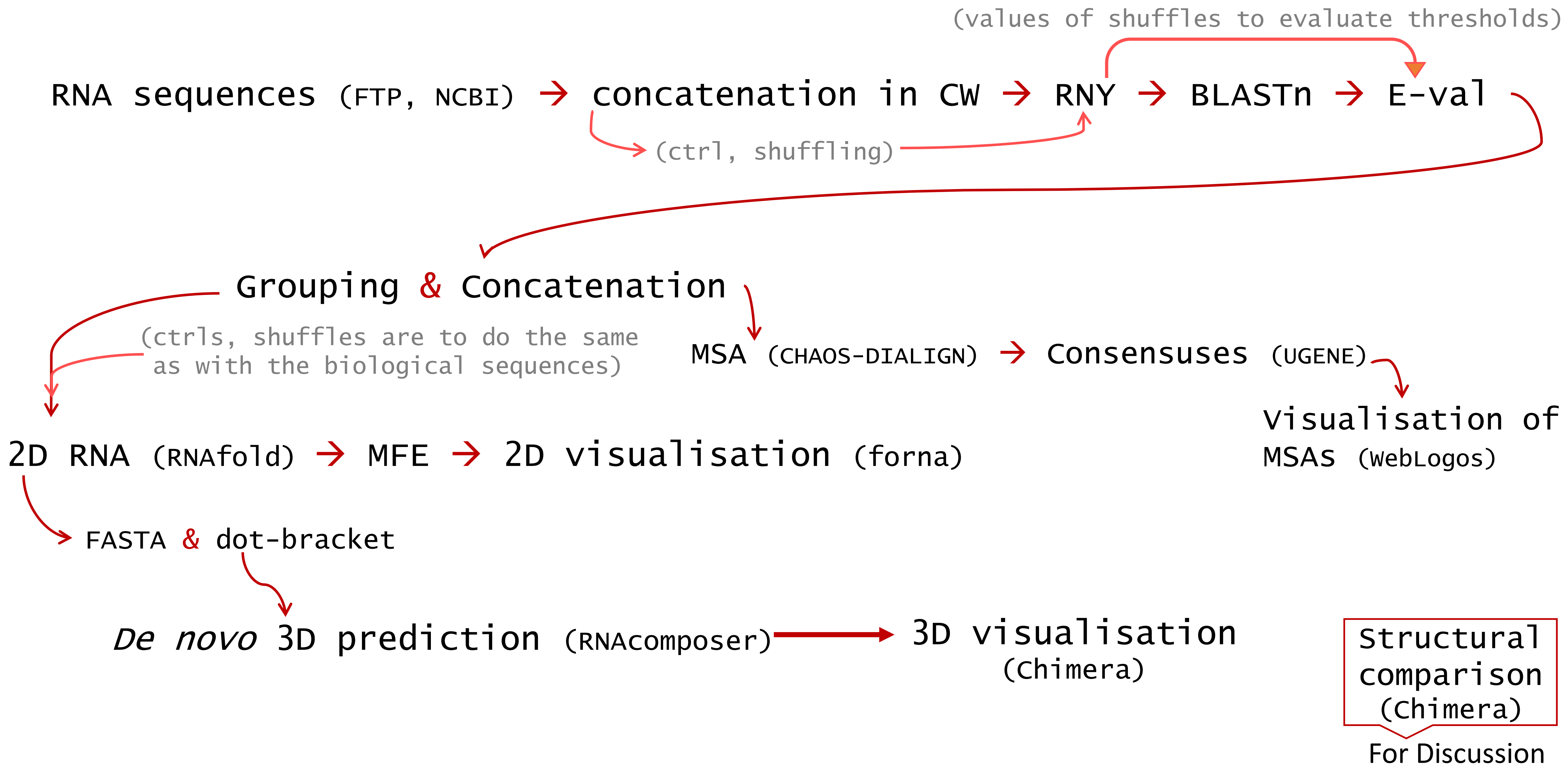

Supplement: Supplementary file 1 [file genes-14-02158-s001.zip › S1_Ur-RNAome_FlowChart-Methodology.pdf]
