# Supplementary material for: A Proposal of the Ur-RNAome
Source: Genes (Basel). 2023 Nov 29;14(12):2158. doi: 10.3390/genes14122158 (PMC10743229; doi:10.3390/genes14122158)
Supplement: Supplementary file 1 [file genes-14-02158-s001.zip › S2_Ur-RNAome_TableS1T.pdf]

S2 for **A proposal of the Ur-RNAome**

Miryam Palacios-Pérez [a,b,c,◎] & Marco V. José [a,b,*]

[a] *Theoretical Biology Group, Instituto de Investigaciones Biomédicas, Universidad Nacional Autónoma de México, Ciudad de México, México*

[b] *Member of the Network of Researchers on the Chemical Emergence of Life (NoRCEL)*

[c] *Second vice-president of NoRCEL and Head of NoRCEL's Latin America Hub*

Corresponding authors E-mail addresses:

◎MPP – mir.pape@iibiomedicas.uunam.mx ;

*MVJ – marcojose@iibiomedicas.unam.mx

Table S1T: Minimum free energy (MFE) of the RNA fragments encoded by the RNY triplets and their corresponding negative control, according to *ViennaRNA* software.

RNA fragment encoded by RNY triplets	MFE
**1A**$_{\mathbf{bio.}}$- 5S archaea consensus	-5.91
**1A**$_{\mathbf{ctrl.}}$- 5S archaea consensus shuffled	-3.29
**1B**$_{\mathbf{bio.}}$- 5S bacteria consensus	-6.14
**1A**$_{\mathbf{ctrl.}}$- 5S bacteria consensus shuffled	-3.16
**2A**$_{\mathbf{bio.}}$- 16S archaea consensus	-28.95
**2A**$_{\mathbf{ctrl.}}$- 16S archaea consensus shuffled	-20.97
**2B**$_{\mathbf{bio.}}$- 16S bacteria consensus	-11.74
**2B**$_{\mathbf{bio.}}$- 16S bacteria consensus shuffled	-10.66
**3A**$_{\mathbf{bio.}}$- 23S archaea consensus	-11.59
**3A**$_{\mathbf{ctrl.}}$- 23S archaea consensus shuffled	-8.92
**3B**$_{\mathbf{bio.}}$- 23S bacteria consensus	-22.50
**3B**$_{\mathbf{ctrl.}}$- 23S bacteria consensus shuffled	-20.87
**4A**$_{\mathbf{bio.}}$- RNA-P archaeon Hxvol	-7.24
**4A**$_{\mathbf{ctrl.}}$- RNA-P archaeon Hxvol shuffled	-4.49
**4B**$_{\mathbf{bio.}}$- RNA-P bacterium Mygen	0
**4B**$_{\mathbf{ctrl.}}$- RNA-P bacterium Mygen shuffled	0
**4C**$_{\mathbf{bio.}}$- RNA-P bacterium SynCC	-4.72
**4C**$_{\mathbf{ctrl.}}$- RNA-P bacterium SynCC shuffled	-4.1
**4D**$_{\mathbf{bio.}}$- RNA-P bacterium SynCC a	-1.51
**4D**$_{\mathbf{ctrl.}}$- RNA-P bacterium SynCC a shuffled	-1.21
**4E**$_{\mathbf{bio.}}$- RNA-P bacterium SynCC b	0
**4E**$_{\mathbf{ctrl.}}$- RNA-P bacterium SynCC b shuffled	-0.65
**5A**$_{\mathbf{bio.}}$- SRP archaeon Kocry	-2.48
**5A**$_{\mathbf{ctrl.}}$- SRP archaeon Kocry shuffled	-0.86
**5B**$_{\mathbf{bio.}}$- SRP bacterial-large Basub	0
**5B**$_{\mathbf{ctrl.}}$- SRP bacterial-large Basub shuffled	0
**6A**$_{\mathbf{bio.}}$- RNA 6S bacterium Basub	-0.25
**6A**$_{\mathbf{ctrl.}}$- RNA 6S bacterium Basub shuffled	0

**7A**$_{\mathbf{bio}}$.- tRNA archaeon Haqwa Gln-UUG	-4.87
**7A**$_{\mathbf{ctrl}}$.- tRNA archaeon Haqwa Gln-UUG shuffled	-4.14
**7B**$_{\mathbf{bio}}$.- tRNA archaeon Thgam Asn-GUU	-5.69
**7B**$_{\mathbf{ctrl}}$.- tRNA archaeon Thgam Asn-GUU shuffled	-2.45
**7C**$_{\mathbf{bio}}$.- tRNA archaeon Thsib Asn-GUU	-2.9
**7C**$_{\mathbf{ctrl}}$.- tRNA archaeon Thsib Asn-GUU shuffled	-2.08
**7D**$_{\mathbf{bio}}$.- tRNA bacterium Bobur Gln-UUG	0
**7D**$_{\mathbf{ctrl}}$.- tRNA bacterium Bobur Gln-UUG shuffled	0
**7E**$_{\mathbf{bio}}$.- tRNA bacterium Derad Cys-GCA	-0.8
**7E**$_{\mathbf{ctrl}}$.- tRNA bacterium Derad Cys-GCA shuffled	0
**7F**$_{\mathbf{bio}}$.- tRNA bacterium Derad Gln-CUG	0
**7F**$_{\mathbf{ctrl}}$.- tRNA bacterium Derad Gln-CUG shuffled	0
**7G**$_{\mathbf{bio}}$.- tRNA bacterium Derad Glu-UUC	0
**7G**$_{\mathbf{ctrl}}$.- tRNA bacterium Derad Glu-UUC shuffled	0
**7H**$_{\mathbf{bio}}$.- tRNA bacterium Derad Gly-UCC	0
**7H**$_{\mathbf{ctrl}}$.- tRNA bacterium Derad Gly-UCC shuffled	0
**7I**$_{\mathbf{bio}}$.- tRNA bacterium Peubi Gly-GCC	-2.97
**7I**$_{\mathbf{ctrl}}$.- tRNA bacterium Peubi Gly-GCC shuffled	-0.29
**7J**$_{\mathbf{bio}}$.- tRNA bacterium SagA Asn-GUU	0
**7J**$_{\mathbf{ctrl}}$.- tRNA bacterium SagA Asn-GUU shuffled	0
**7K**$_{\mathbf{bio}}$.- tRNA bacterium SagA Thr-GGU	-2.4
**7K**$_{\mathbf{ctrl}}$.- tRNA bacterium SagA Thr-GGU shuffled	0
**7L**$_{\mathbf{bio}}$.- tRNA bacterium Thmar Phe-GAA	0
**7L**$_{\mathbf{ctrl}}$.- tRNA bacterium Thmar Phe-GAA shuffled	-0.30

Only the underlined letters in some of the tRNA anticodons were recovered using the approach outlined in the article, in some cases completely and in others only partially.
