# Supplementary material for: A Proposal of the Ur-RNAome
Source: Genes (Basel). 2023 Nov 29;14(12):2158. doi: 10.3390/genes14122158 (PMC10743229; doi:10.3390/genes14122158)
Supplement: Supplementary file 1 [file genes-14-02158-s001.zip › S3_Ur-RNAome_AllRNAstructures.pdf]

S3 for **A proposal of the Ur-RNAome**

Miryam Palacios-Pérez [a,b,c,◎] & Marco V. José [a,b,*]

[a] *Theoretical Biology Group, Instituto de Investigaciones Biomédicas, Universidad Nacional Autónoma de México, Ciudad de México, México*
[b] *Member of the Network of Researchers on the Chemical Emergence of Life (NoRCEL)*
[c] *Second vice-president of NoRCEL and Head of NoRCEL's Latin America Hub*
Corresponding authors E-mail addresses:
[◎]MPP – mir.pape@iibiomedicas.uunam.mx ; [*]MVJ – marcojose@iibiomedicas.unam.mx

The minimum free energy (MFE) is provided for each RNA, either the biological or the corresponding shuffled ones.

S1.- 5S rRNA, **RNY**.
A_{bio}.- Archaea: logo, 2D & 3D.
A_{ctrl}.- Archaea, shuffled: 2D & 3D.
B_{bio}.- Bacteria: logo, 2D & 3D.
B_{ctrl}.- Bacteria, shuffled: 2D & 3D.

S2.- 16S rRNA, **RNY**
A_{bio}.- Archaea: logo, 2D & 3D.
A_{ctrl}.- Archaea, shuffled: 2D & 3D.
B_{bio}.- Bacteria: logo, 2D & 3D.
B_{ctrl}.- Bacteria, shuffled: 2D & 3D.

S3.- 23S rRNA, **RNY**
A_{bio}.- Archaea: logo, 2D & 3D.
A_{ctrl}.- Archaea, shuffled: 2D & 3D.
B_{bio}.- Bacteria: logo, 2D & 3D.
B_{ctrl}.- Bacteria, shuffled: 2D & 3D.

S4.- RNA-P, **RNY**
A_{bio}.- Of the archaeon "Hxvol": 2D & 3D.
A_{ctrl}.- Of the archaeon "Hxvol", shuffled: 2D & 3D.
B_{bio}.- Of the bacterium "Mygen": 2D & 3D.
B_{ctrl}.- Of the bacterium "Mygen", shuffled: 2D & 3D.
C_{bio}.- Of the bacterium "SynCC": 2D & 3D.
C_{ctrl}.- Of the bacterium "SynCC", shuffled: 2D & 3D.
D_{bio}.- Of the bacterium "SynCC", fragment 1: 2D & 3D.
D_{ctrl}.- Of the bacterium "SynCC", fragment 1, shuffled: 2D & 3D.
E_{bio}.- Of the bacterium "SynCC", fragment 2: 2D & 3D.
E_{ctrl}.- Of the bacterium "SynCC", fragment 2, shuffled: 2D & 3D.

S5.- SRP-RNA, **RNY**
A_{bio}.- Of the archaeon "Kocry": 2D & 3D.
A_{ctrl}.- Of the archaeon "Kocry", shuffled: 2D & 3D.
B_{bio}.- Large version, from the bacterium "Basub": 2D & 3D.
B_{ctrl}.- Large version, from the bacterium "Basub", shuffled: 2D & 3D.

S6.- 6S RNA, **RNY**
A_{bio}.- RNA 6S of the bacterium "Basub": 2D & 3D.
A_{ctrl}.- RNA 6S of the bacterium "Basub", shuffled: 2D & 3D.

S7.- tRNA, **RNY**
A_{bio}.- Of the archaeon "Haqwa", for Gln-UUG: 2D & 3D.
A_{ctrl}.- Of the archaeon "Haqwa", for Gln-UUG, shuffled: 2D & 3D.
B_{bio}.- Of the archaeon "Thgam", for Asn-GUU: 2D & 3D.
B_{ctrl}.- Of the archaeon "Thgam", for Asn-GUU, shuffled: 2D & 3D.
C_{bio}.- Of the archaeon "Thsib", for Asn-GUU: 2D & 3D.
C_{ctrl}.- Of the archaeon "Thsib", for Asn-GUU, shuffled: 2D & 3D.
D_{bio}.- Of the bacterium "Bobur", for Gln-UUG: 2D & 3D.
D_{ctrl}.- Of the bacterium "Bobur", for Gln-UUG, shuffled: 2D & 3D.
E_{bio}.- Of the bacterium "Derad", for Cys-GCA: 2D & 3D.
E_{ctrl}.- Of the bacterium "Derad", for Cys-GCA, shuffled: 2D & 3D.
F_{bio}.- Of the bacterium "Derad", for Gln-CUG: 2D & 3D.
F_{ctrl}.- Of the bacterium "Derad", for Gln-CUG, shuffled: 2D & 3D.
G_{bio}.- Of the bacterium "Derad", for Glu-UUC: 2D & 3D.
G_{ctrl}.- Of the bacterium "Derad", for Glu-UUC, shuffled: 2D & 3D.
H_{bio}.- Of the bacterium "Derad", for Gly-UCC: 2D & 3D.
H_{ctrl}.- Of the bacterium "Derad", for Gly-UCC, shuffled: 2D & 3D.
I_{bio}.- Of the bacterium "Peubi", for Gly-GCC: 2D & 3D.
I_{ctrl}.- Of the bacterium "Peubi", for Gly-GCC, shuffled: 2D & 3D.
J_{bio}.- Of the bacterium "SagA", for Asn-GUU: 2D & 3D.
J_{ctrl}.- Of the bacterium "SagA", for Asn-GUU, shuffled: 2D & 3D.
K_{bio}.- Of the bacterium "SagA", for Thr-GGU: 2D & 3D.
K_{ctrl}.- Of the bacterium "SagA", for Thr-GGU, shuffled: 2D & 3D.
L_{bio}.- Of the bacterium "Thmar", for Phe-GAA: 2D & 3D.
L_{ctrl}.- Of the bacterium "Thmar", for Phe-GAA, shuffled: 2D & 3D.

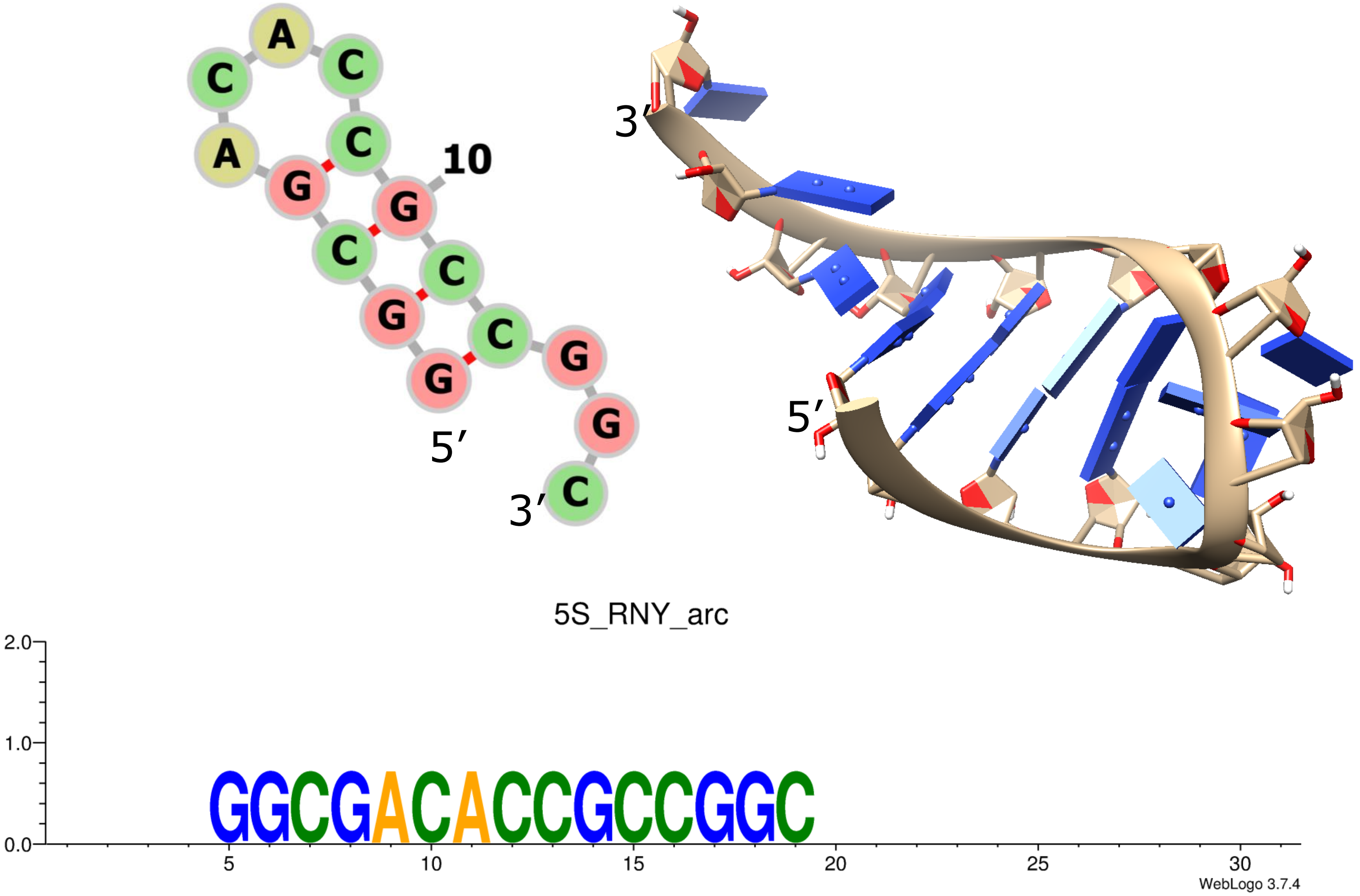

1A$_{bio}$: 5S archaea consensus, MFE = -5.91

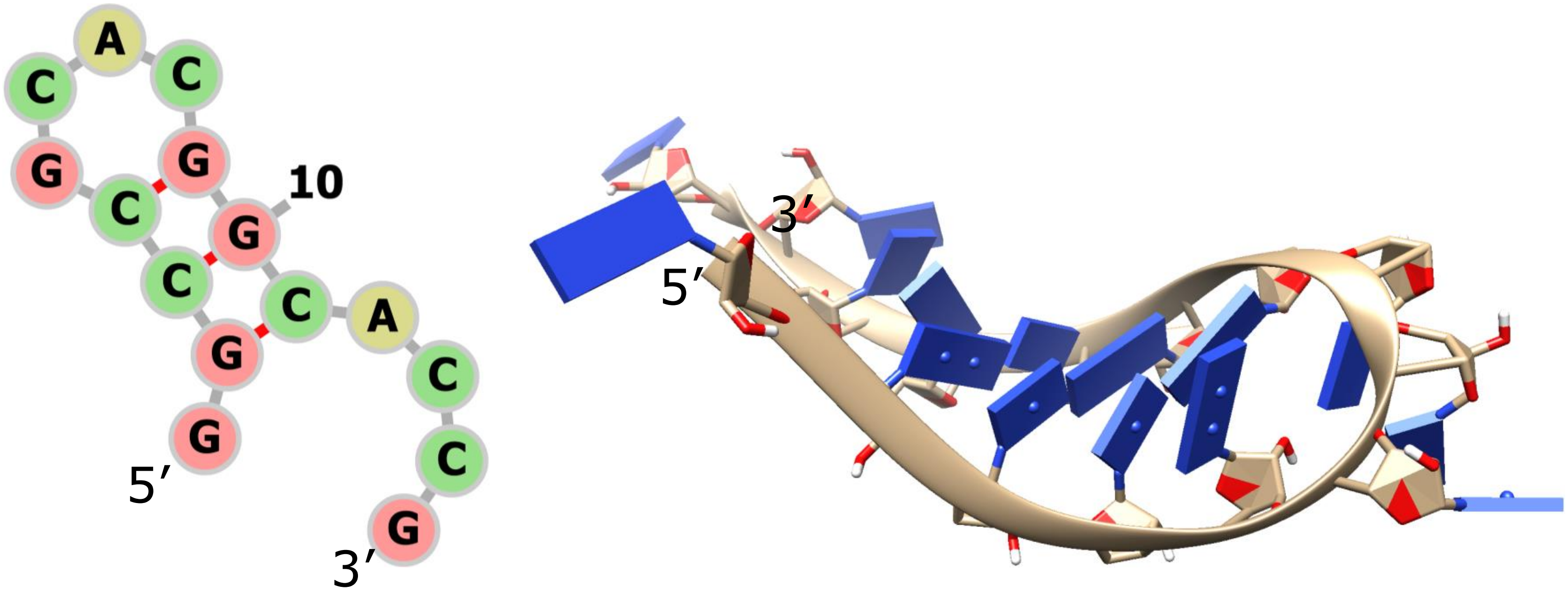

1A$_{ctrl}$: 5S archaea consensus shuffled, MFE = -3.29

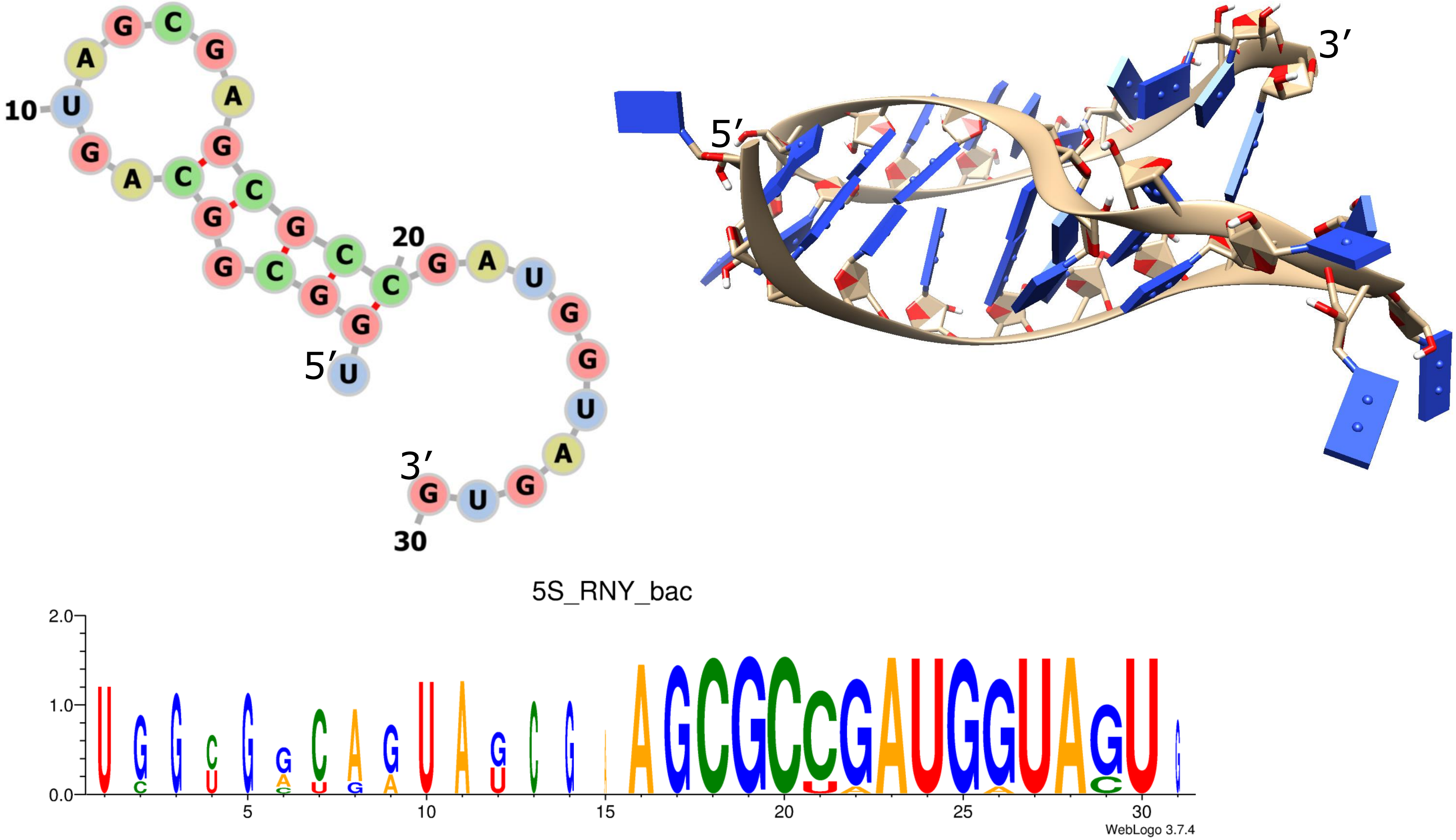

1B$_{\textbf{bio}}$: 5S bacteria consensus, MFE = -6.14

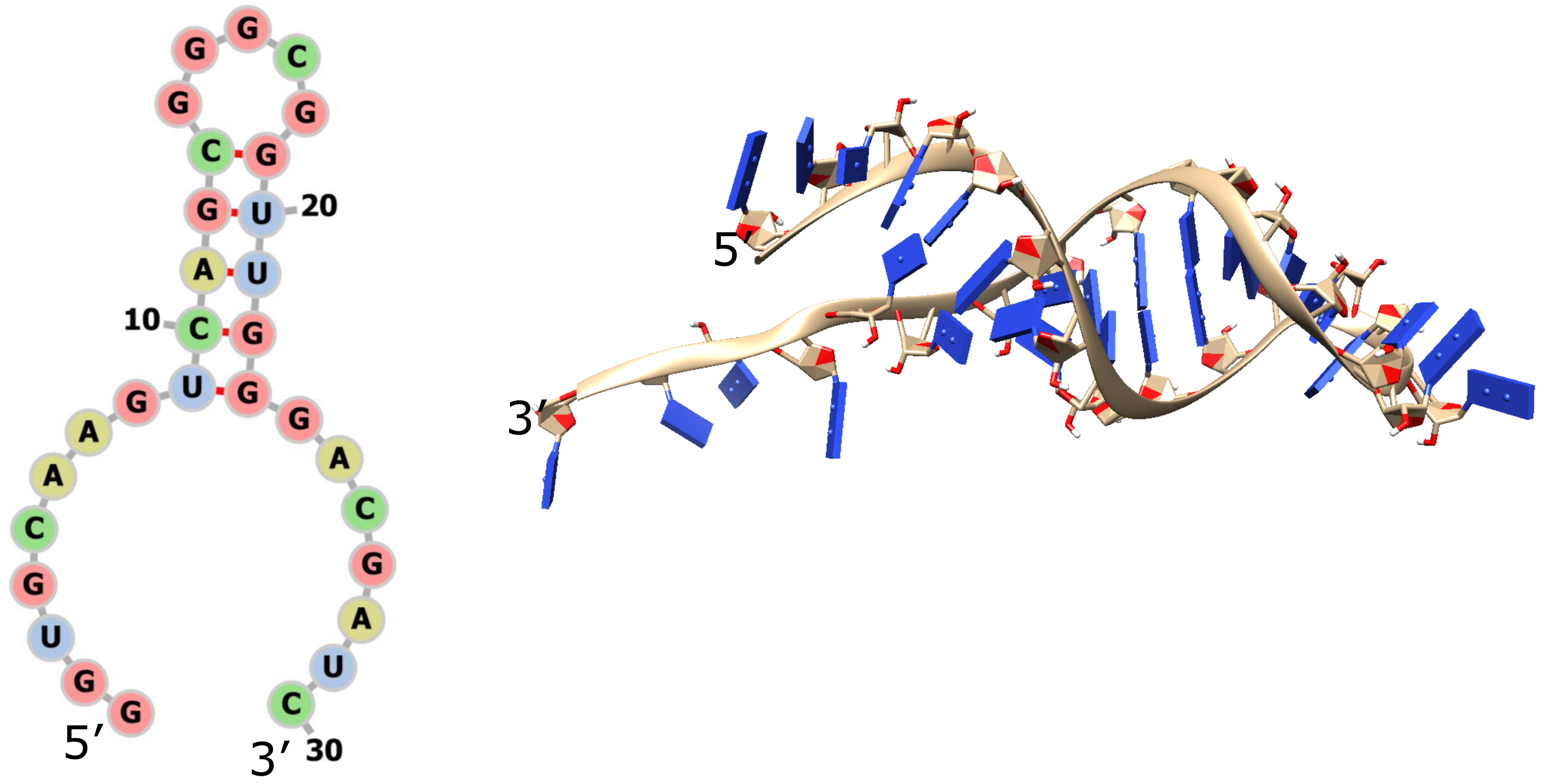

1B$_{ctrl}$: 5S bacteria consensus shuffled, MFE = -3.16

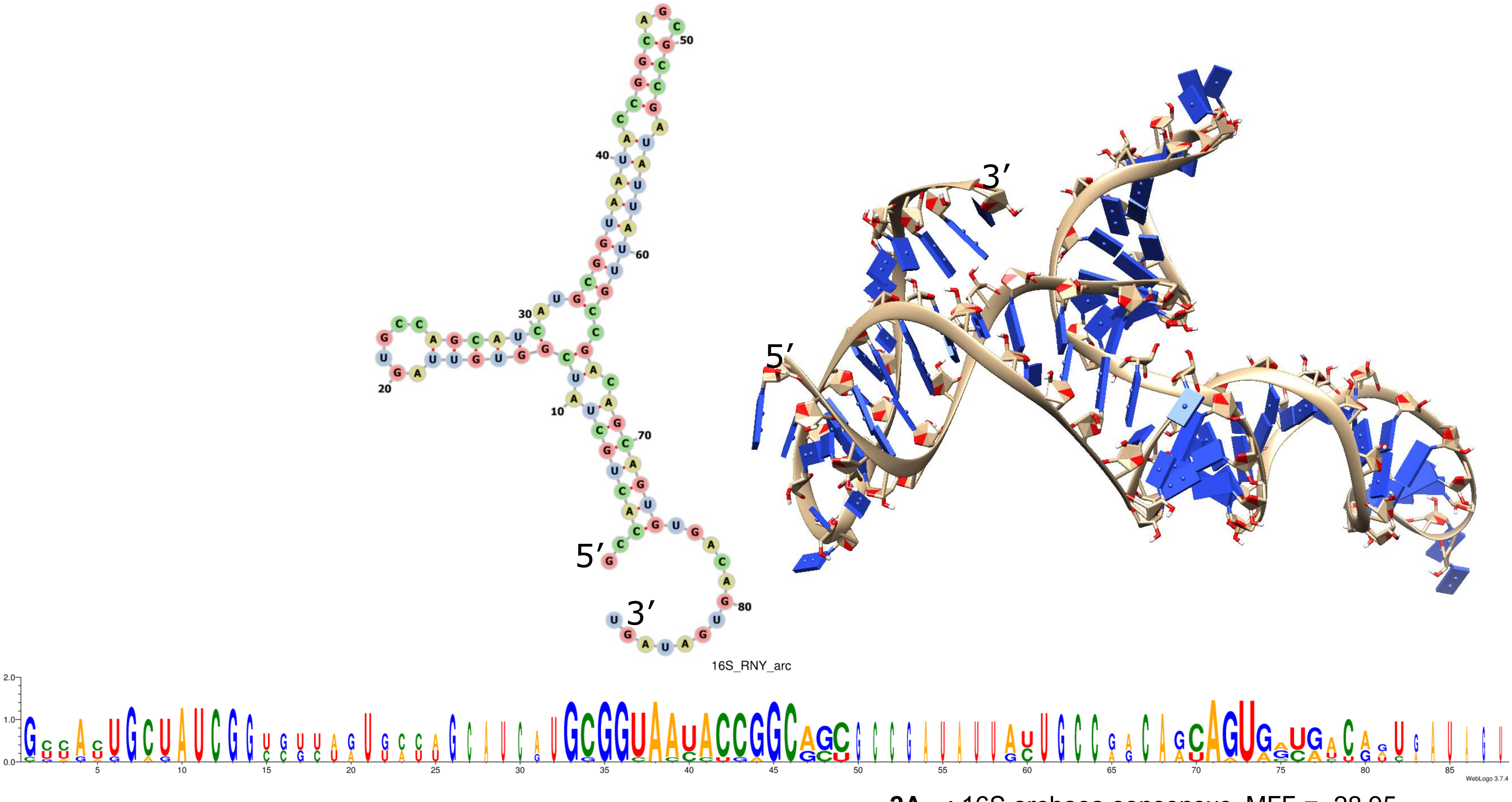

$2A_{bio}$: 16S archaea consensus, MFE = -28.95

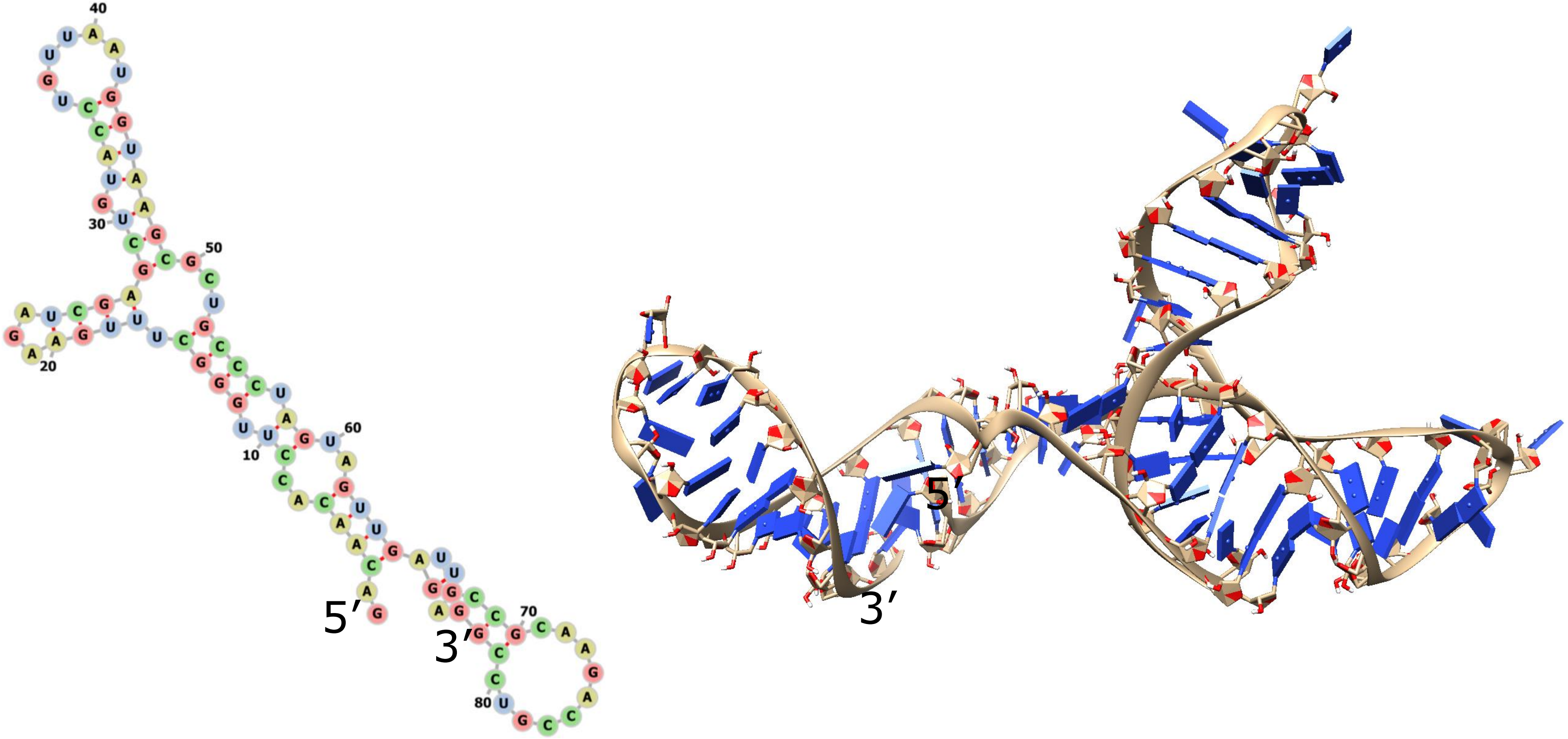

2A$_{ctrl}$: 16S archaea consensus shuffled, MFE = -20.97

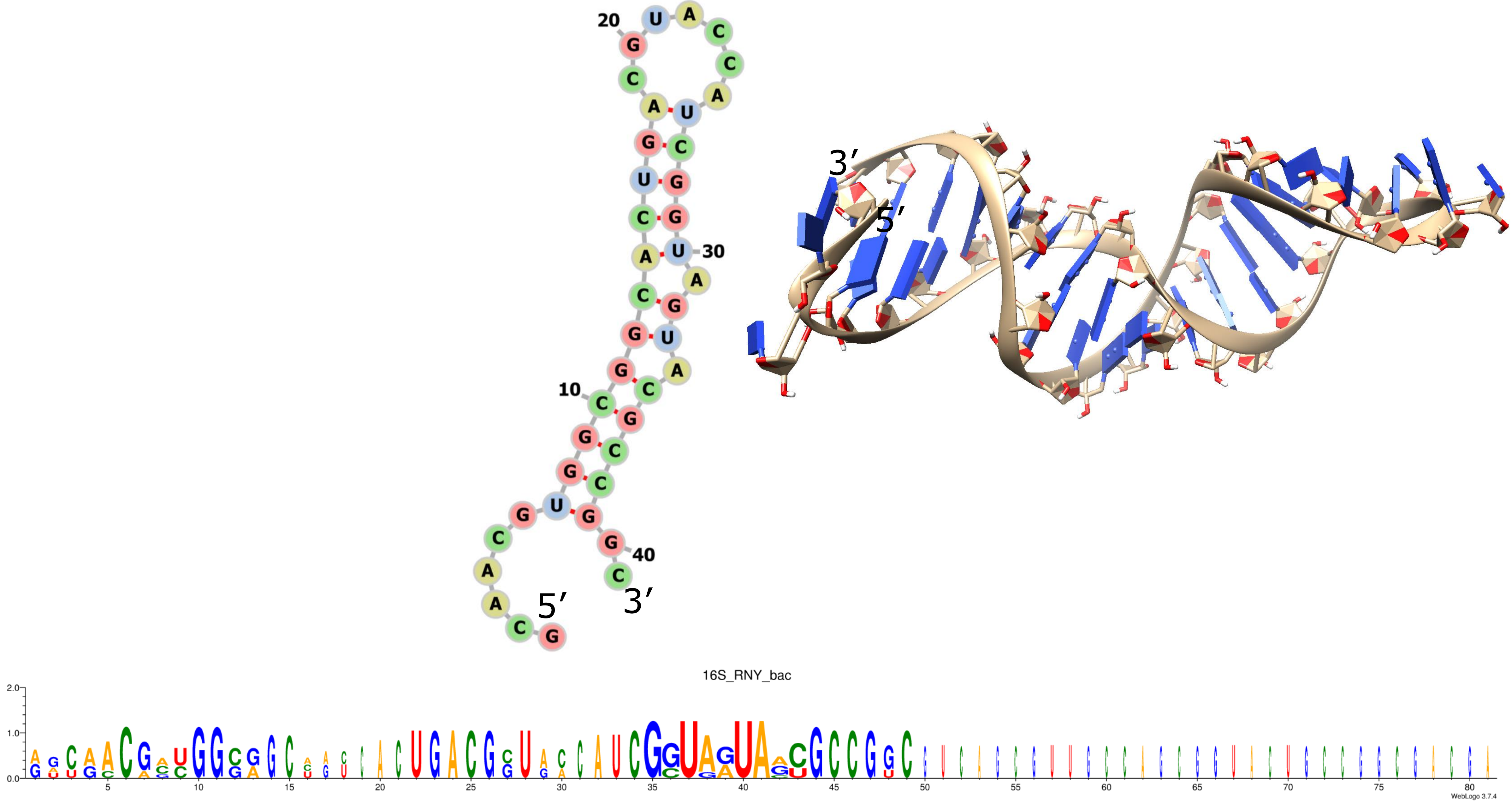

2B$_{bio}$: 16S bacteria consensus, MFE = -11.74

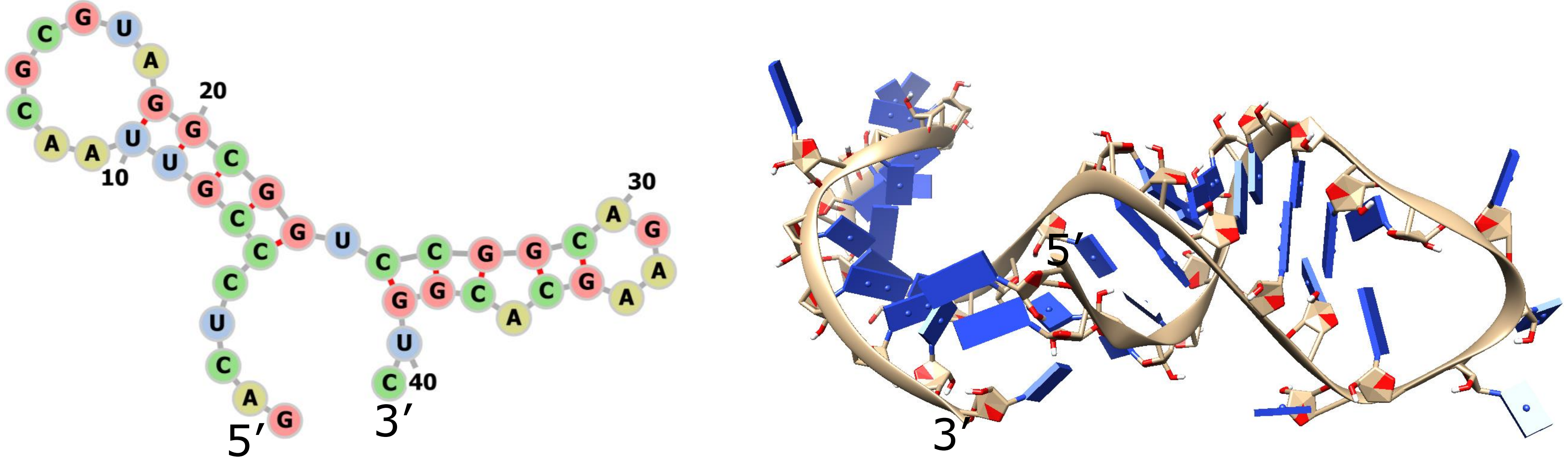

2B$_{ctrl}$: 16S bacteria consensus shuffled, MFE = -10.66

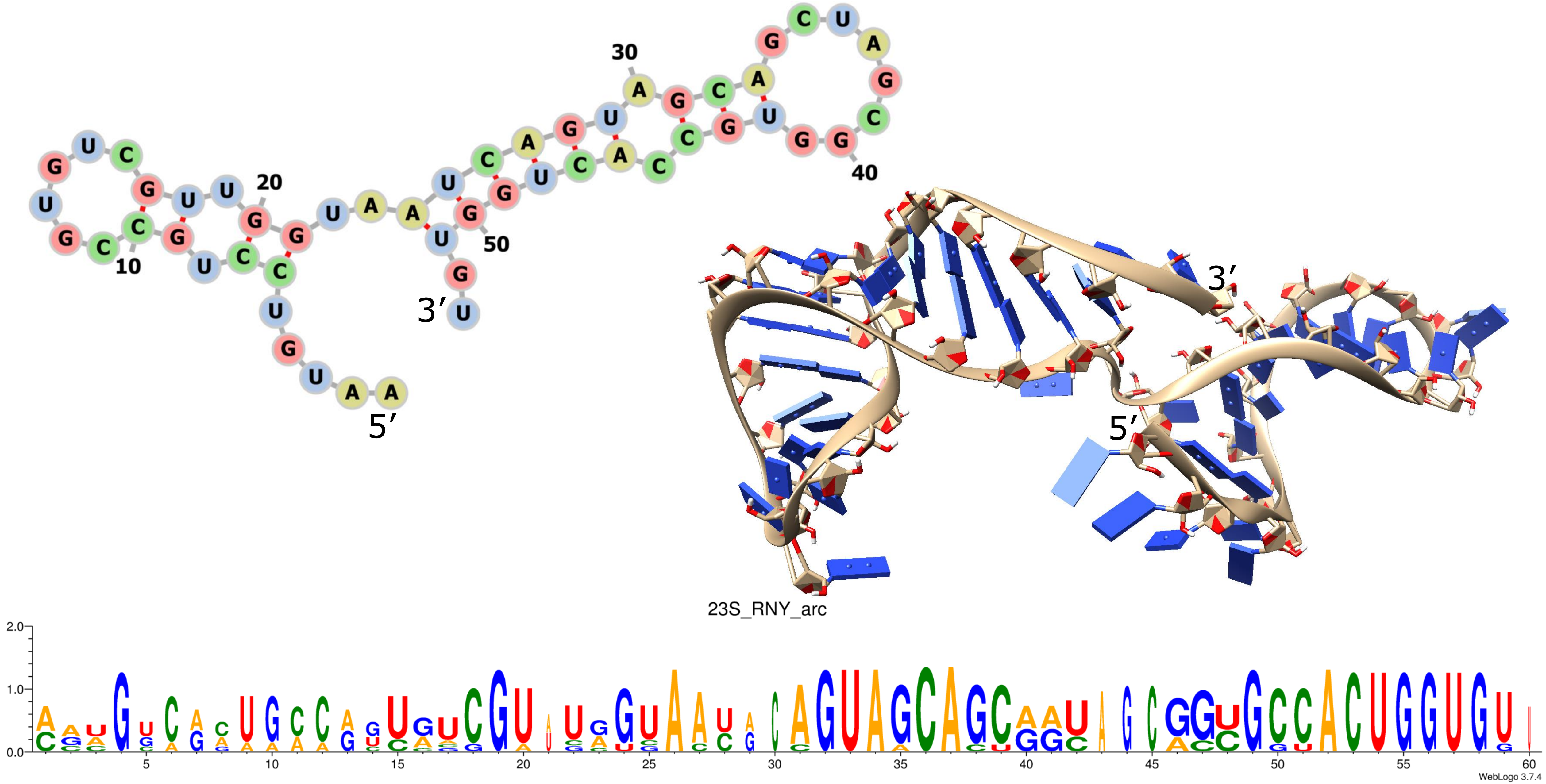

3A$_{bio}$: 23S archaea consensus, MFE = -11.59

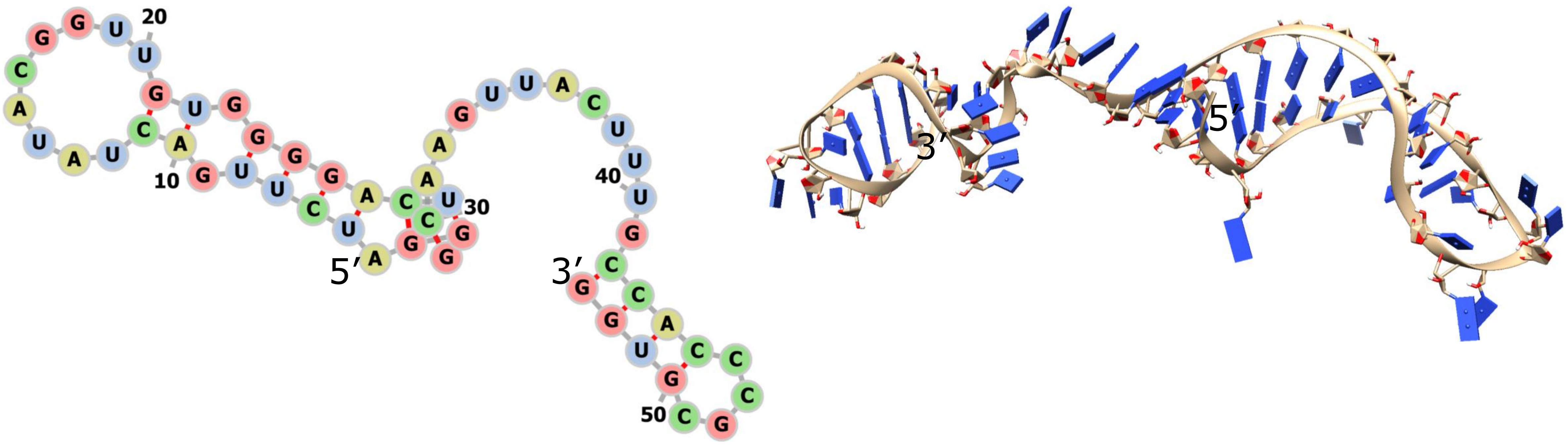

$3A_{ctrl}$: 23S archaea consensus shuffled, MFE = -8.92

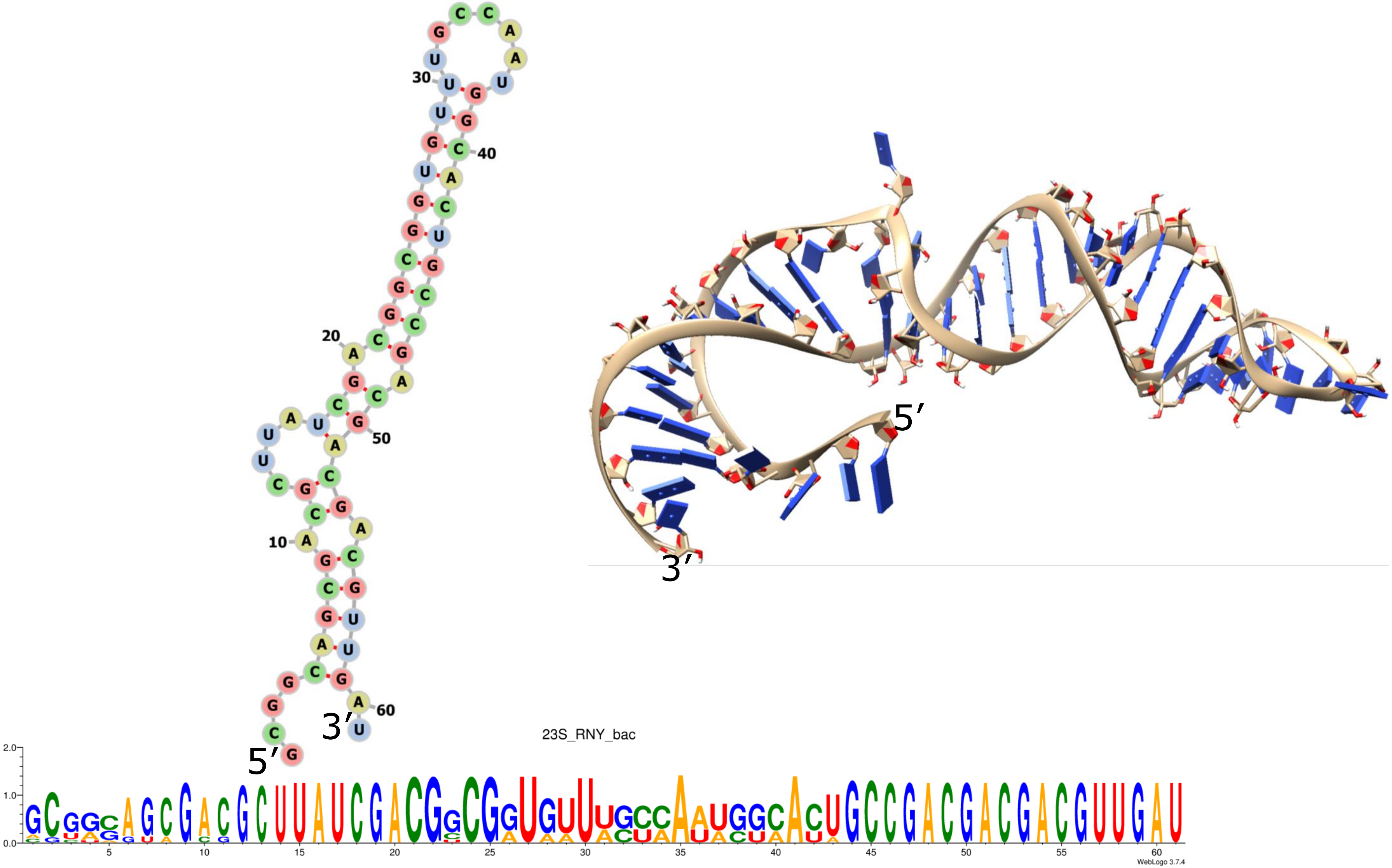

$3B_{bio}$: 23S bacteria consensus, MFE = -22.50

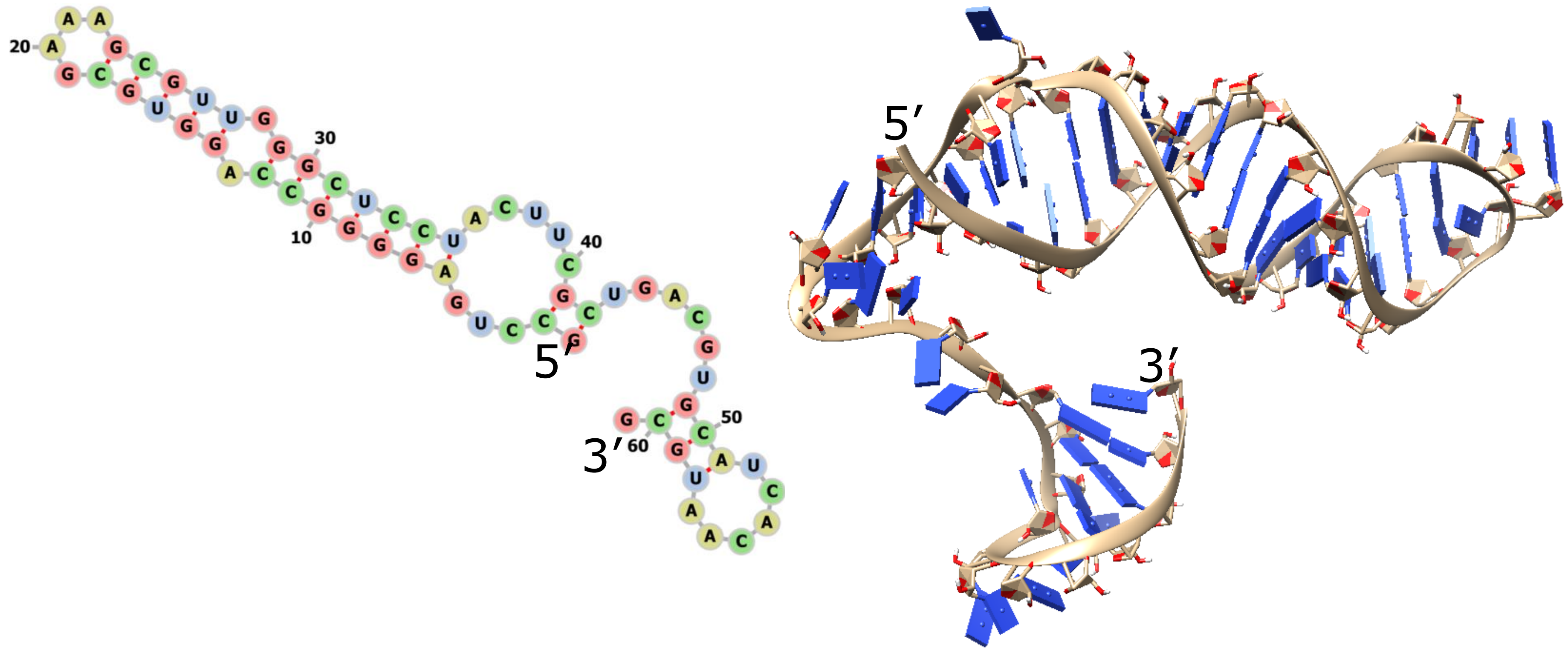

3B$_{ctrl}$: 23S bacteria consensus shuffled, MFE = -20.87

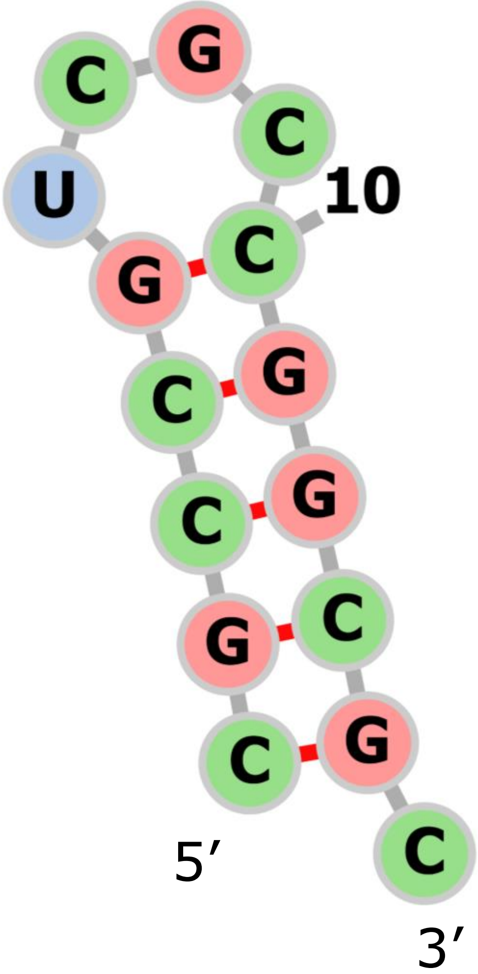

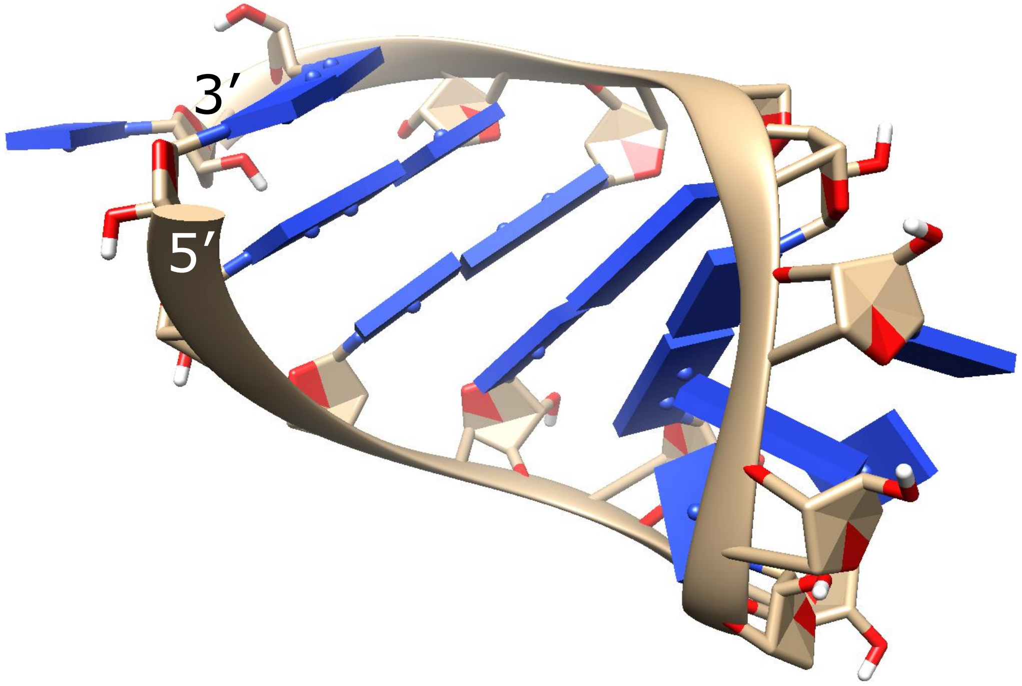

4A$_{bio}$: RNA moiety from RNAseP of the archaeon “Hxvol”,
MFE = -7.24

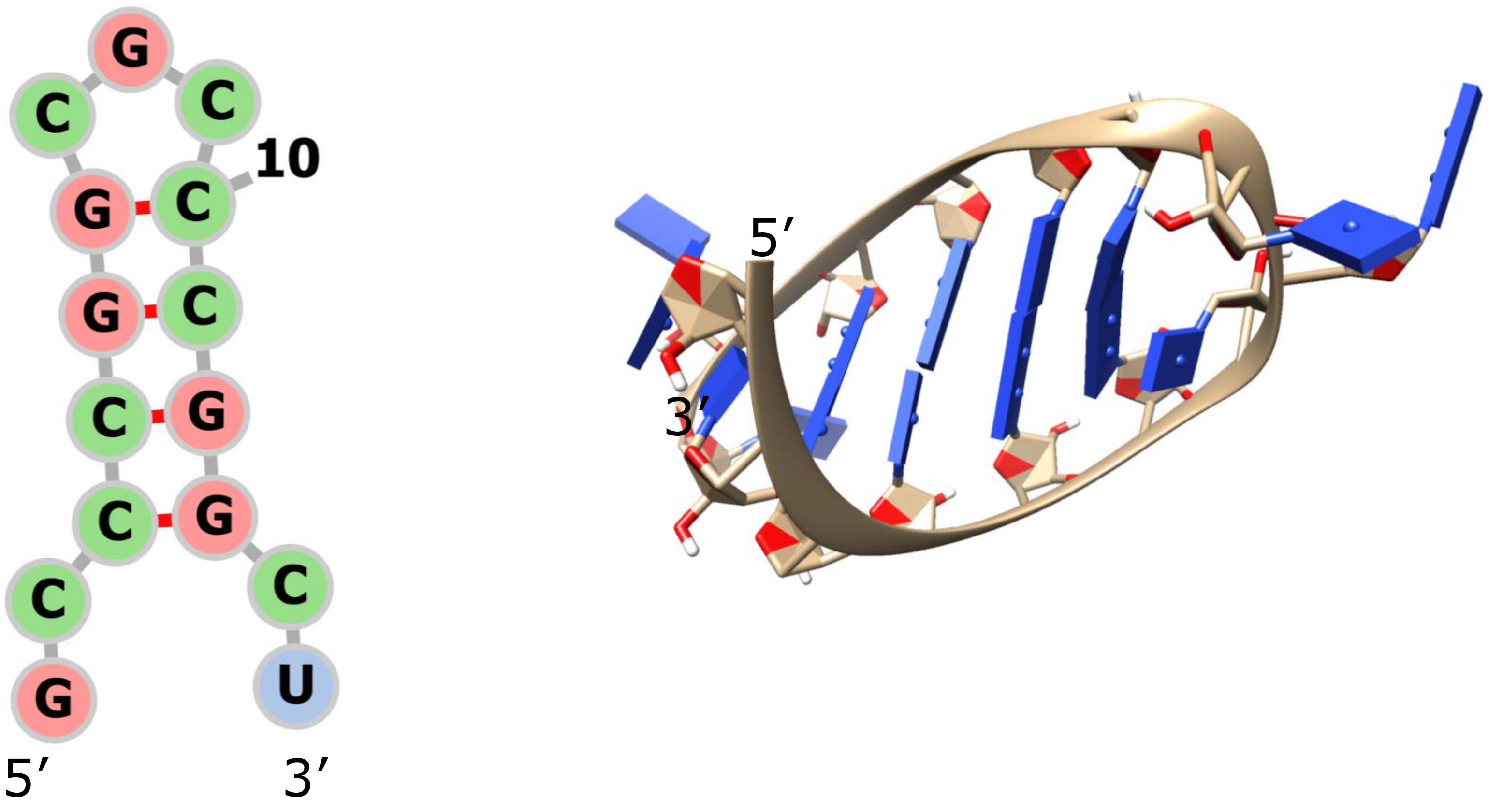

4A$_{ctrl}$: RNA moiety from RNAseP of the archaeon “Hxvol”, shuffled, MFE = -4.49

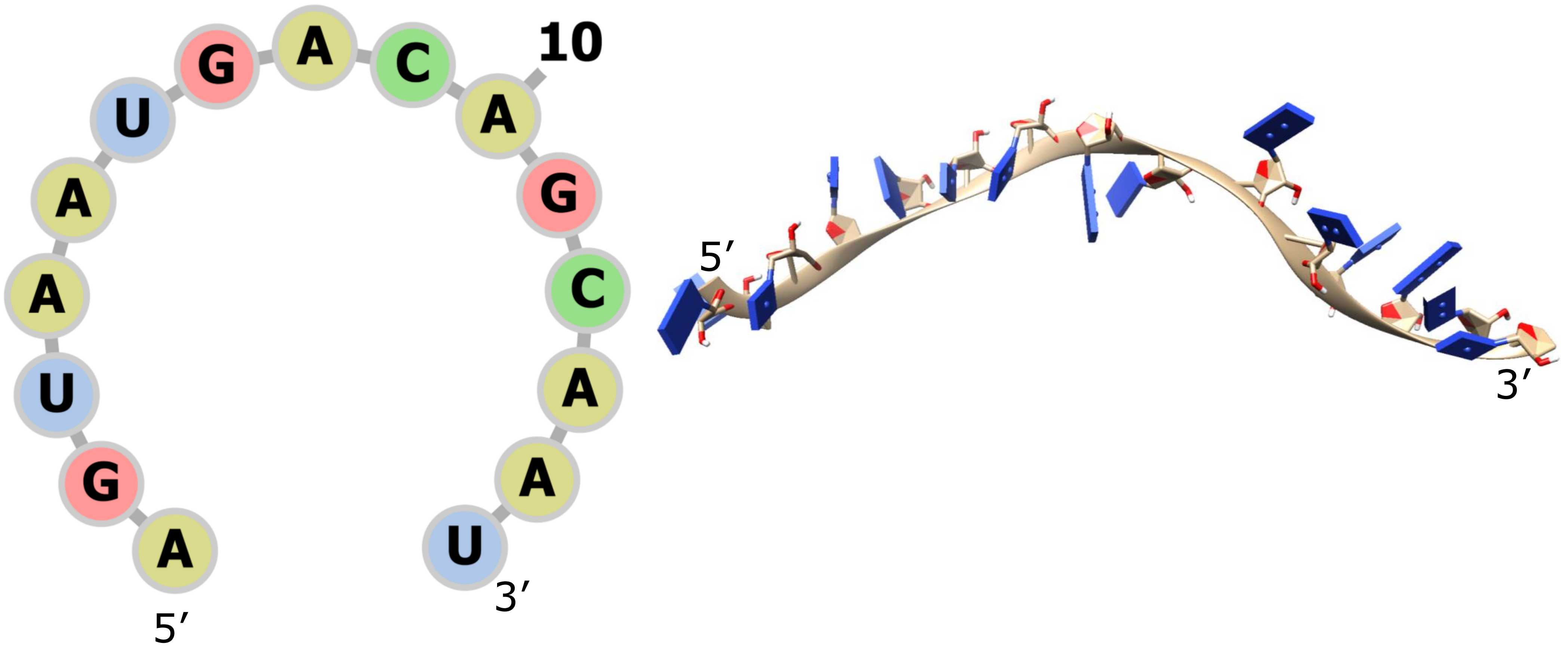

4B$_{bio}$: RNA moiety from RNAseP of the bacterium “Mygen”,
MFE = 0.0

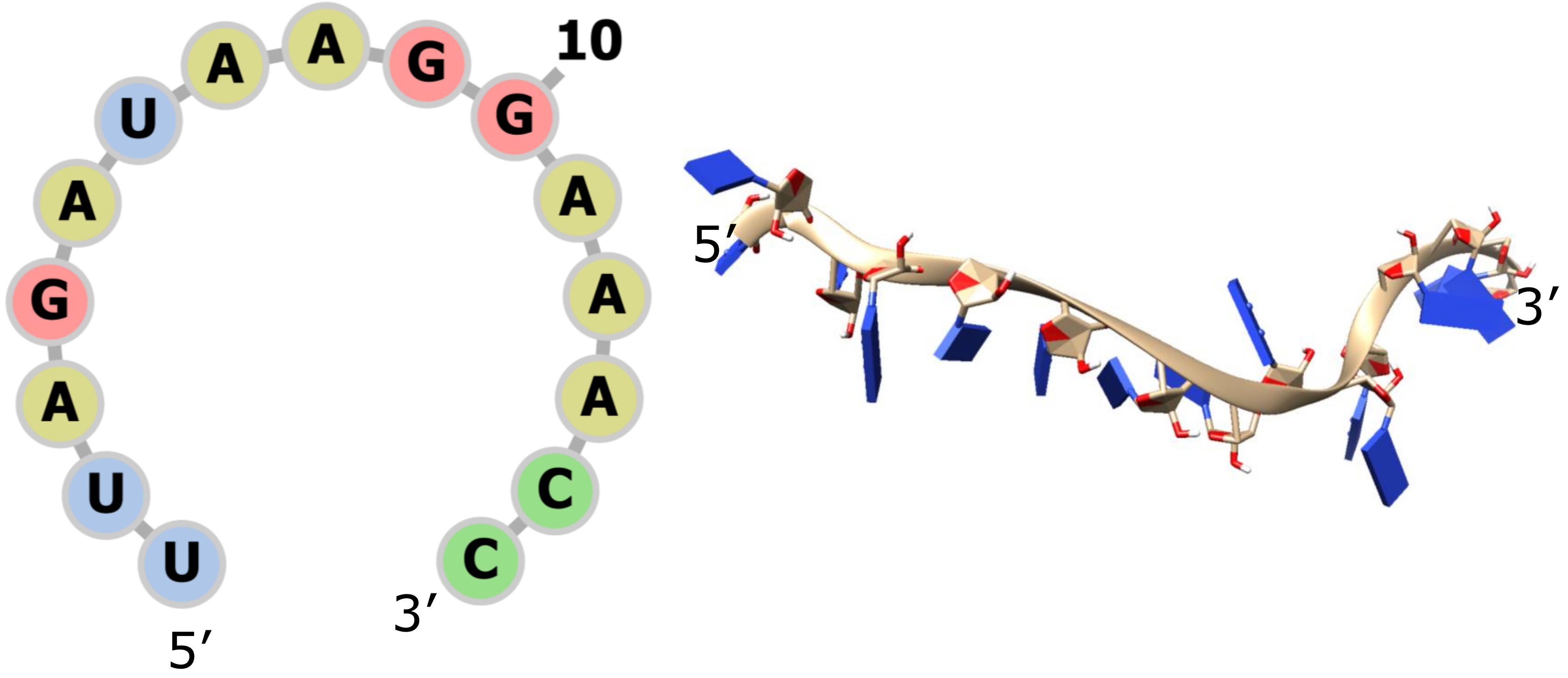

$4B_{ctrl}$: RNA moiety from RNAseP of the bacterium "Mygen", shuffled, MFE = 0.0

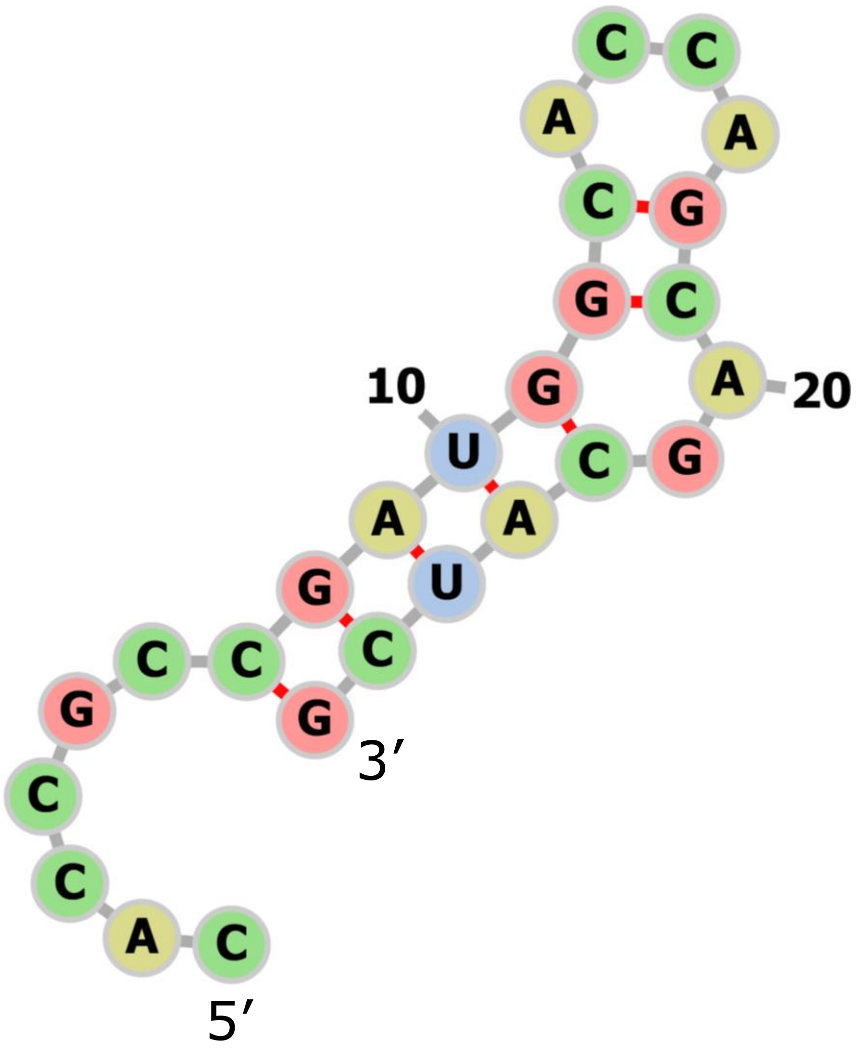

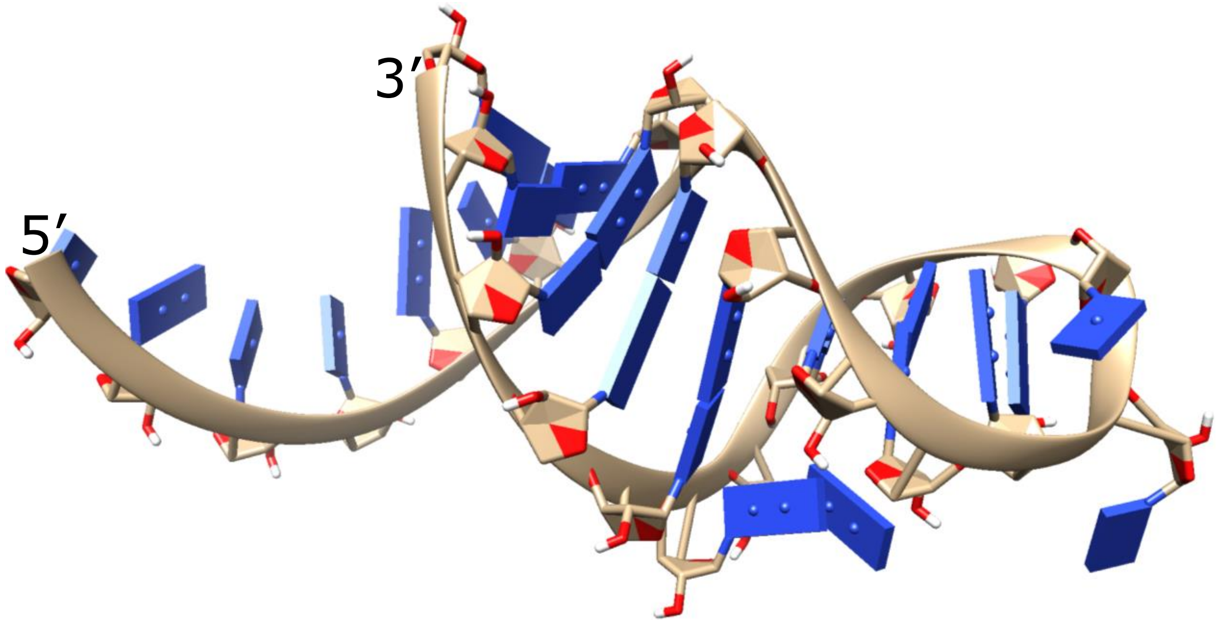

4C$_{bio}$: RNA moiety from RNAseP of the bacterium “SynCC”,
MFE = -4.72

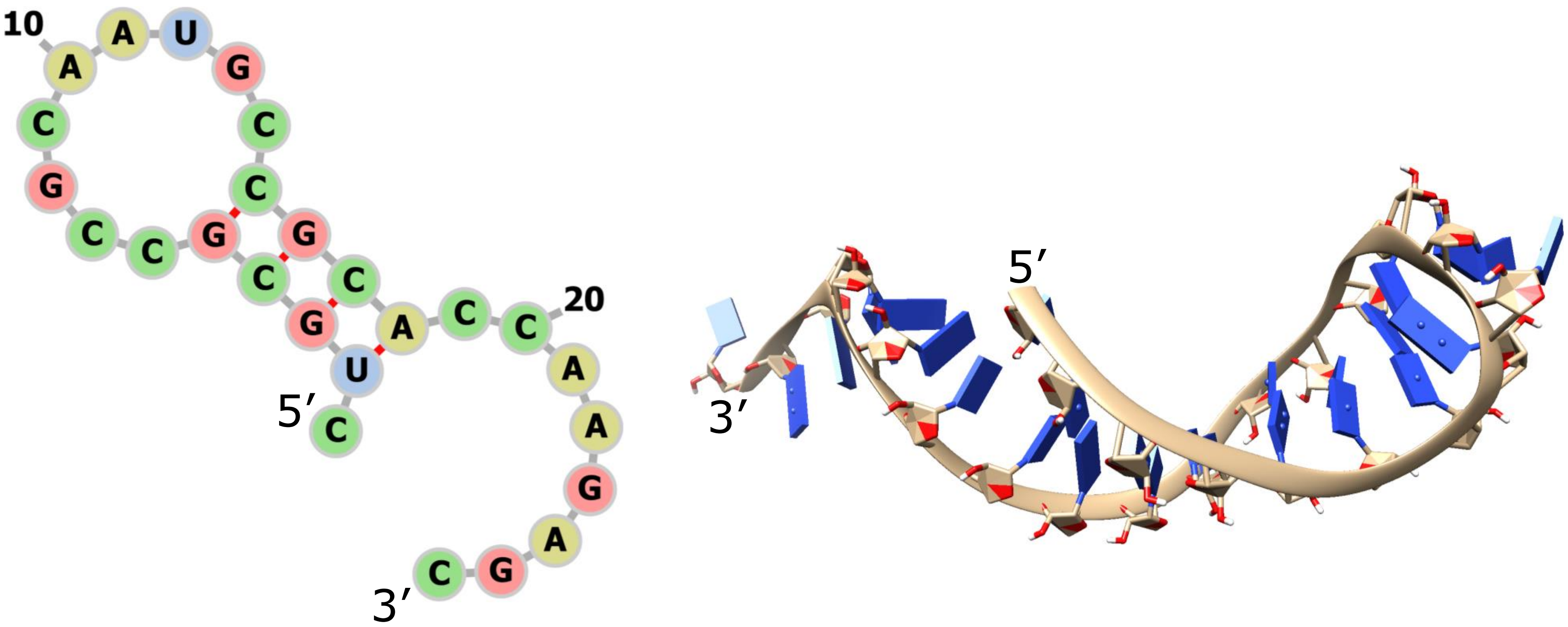

4C$_{ctrl}$: RNA moiety from RNAseP of the bacterium “SynCC”,
shuffled, MFE = -4.10

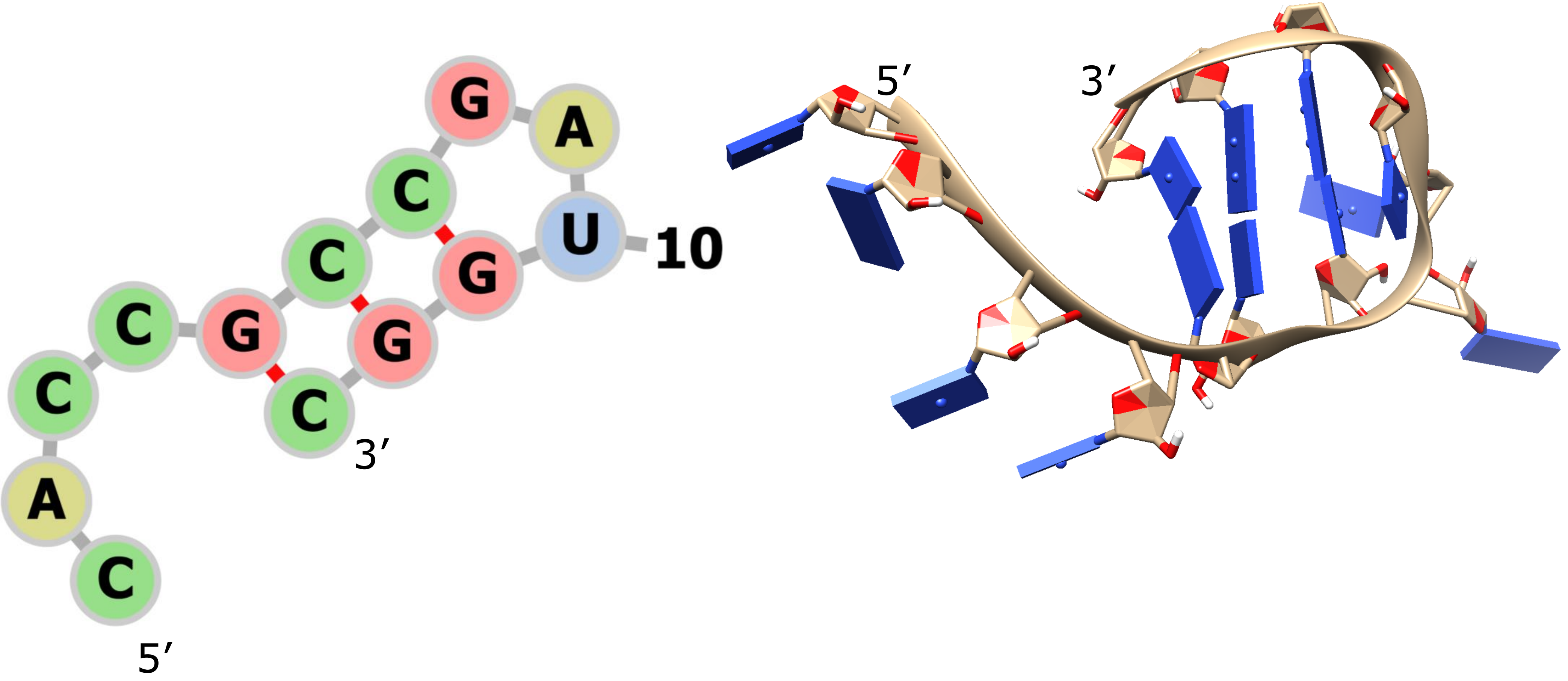

4D$_{bio}$: RNA moiety from RNAseP of the bacterium “SynCC”,
fragment 1, MFE = -1.51

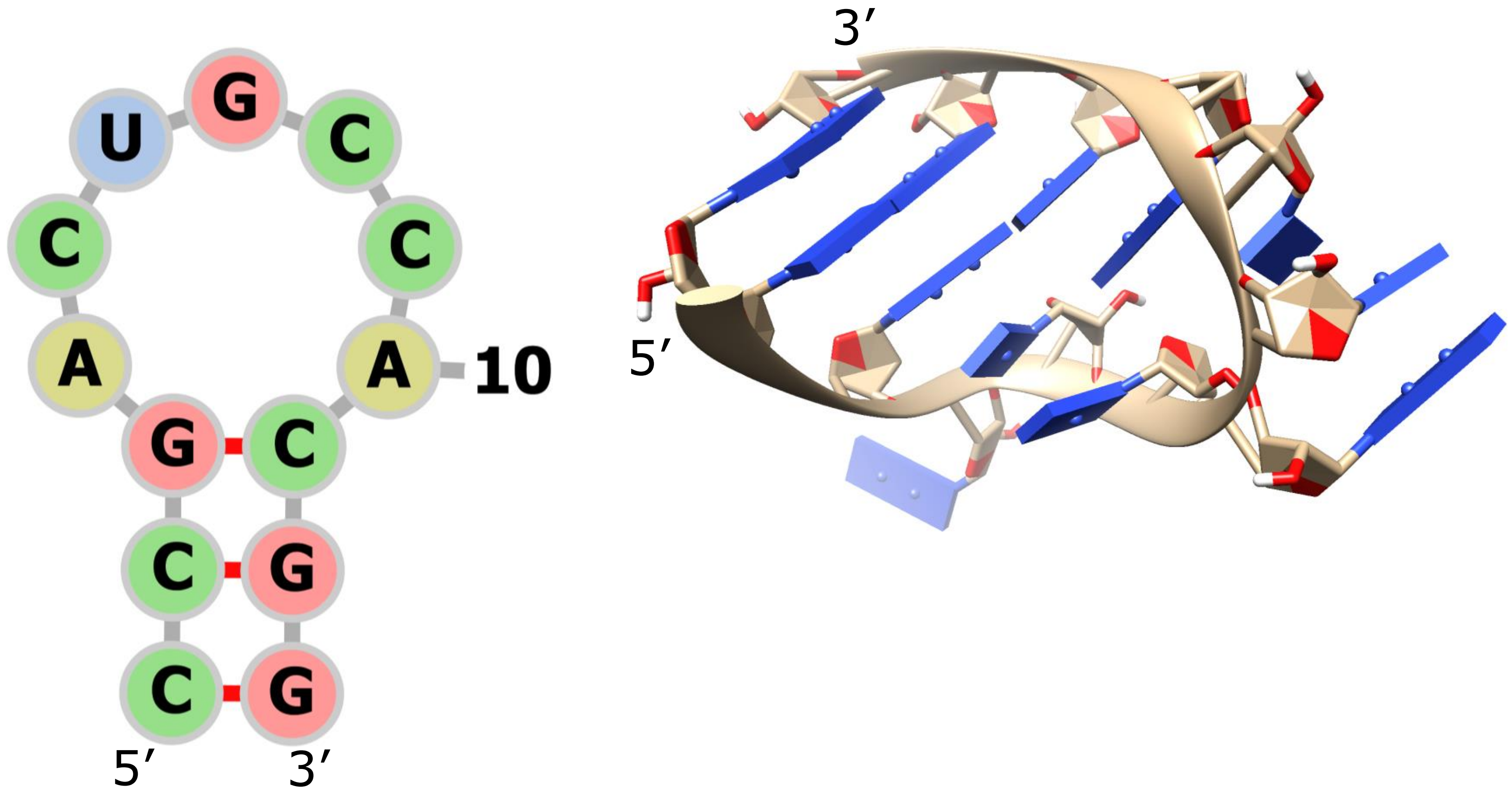

4D$_{ctrl}$: RNA moiety from RNAseP of the bacterium “SynCC”, fragment 1, shuffled, MFE = -1.21

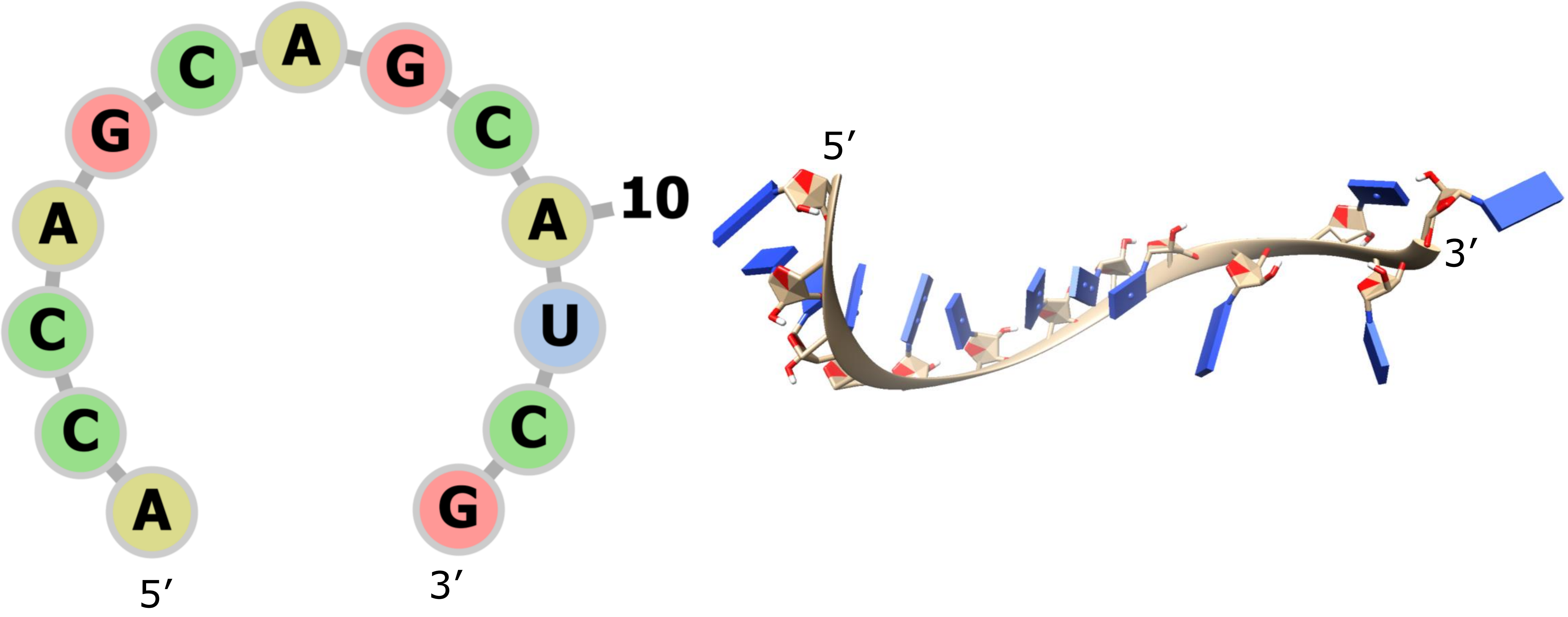

4E$_{bio}$: RNA moiety from RNAseP of the bacterium “SynCC”,
fragment 2, MFE = 0.0

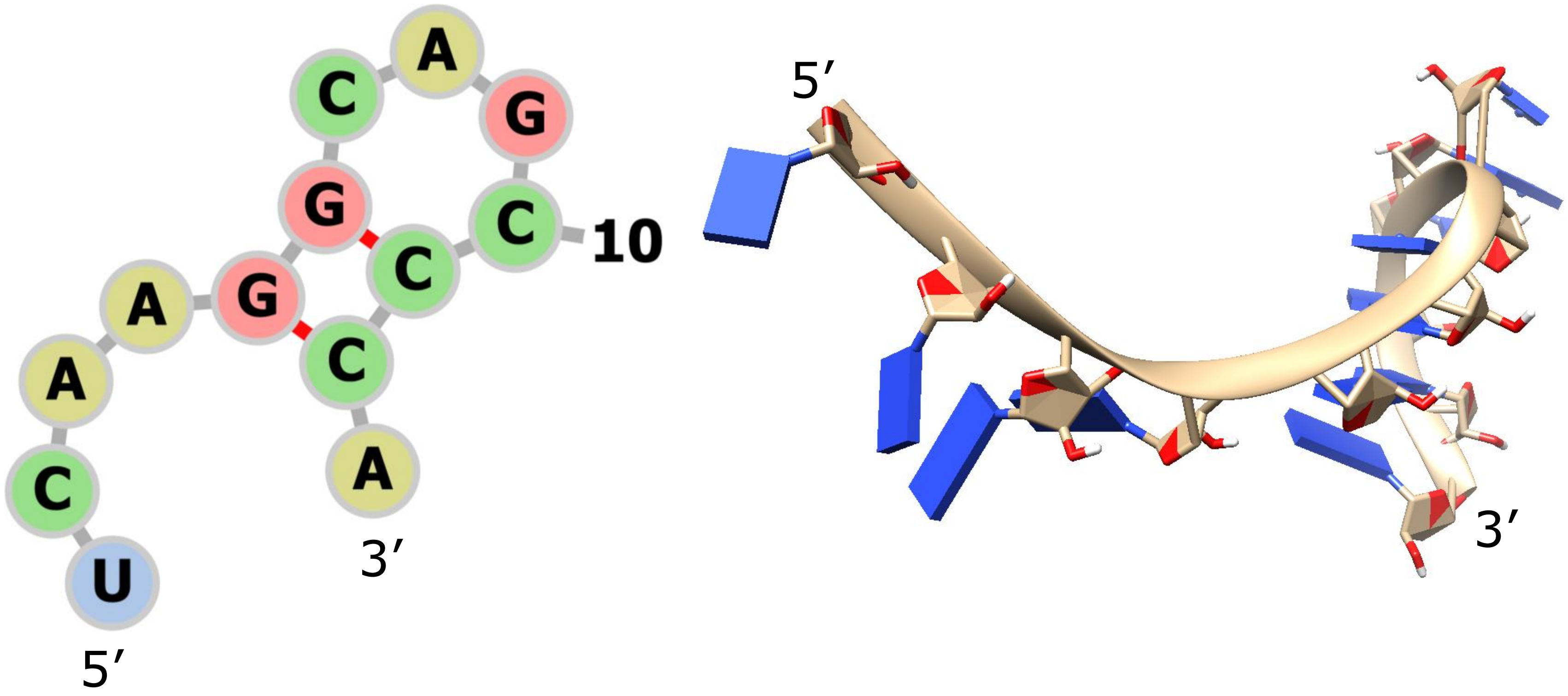

$4E_{ctrl}$: RNA moiety from RNAseP of the bacterium “SynCC”, fragment 2, shuffled, MFE = -0.65

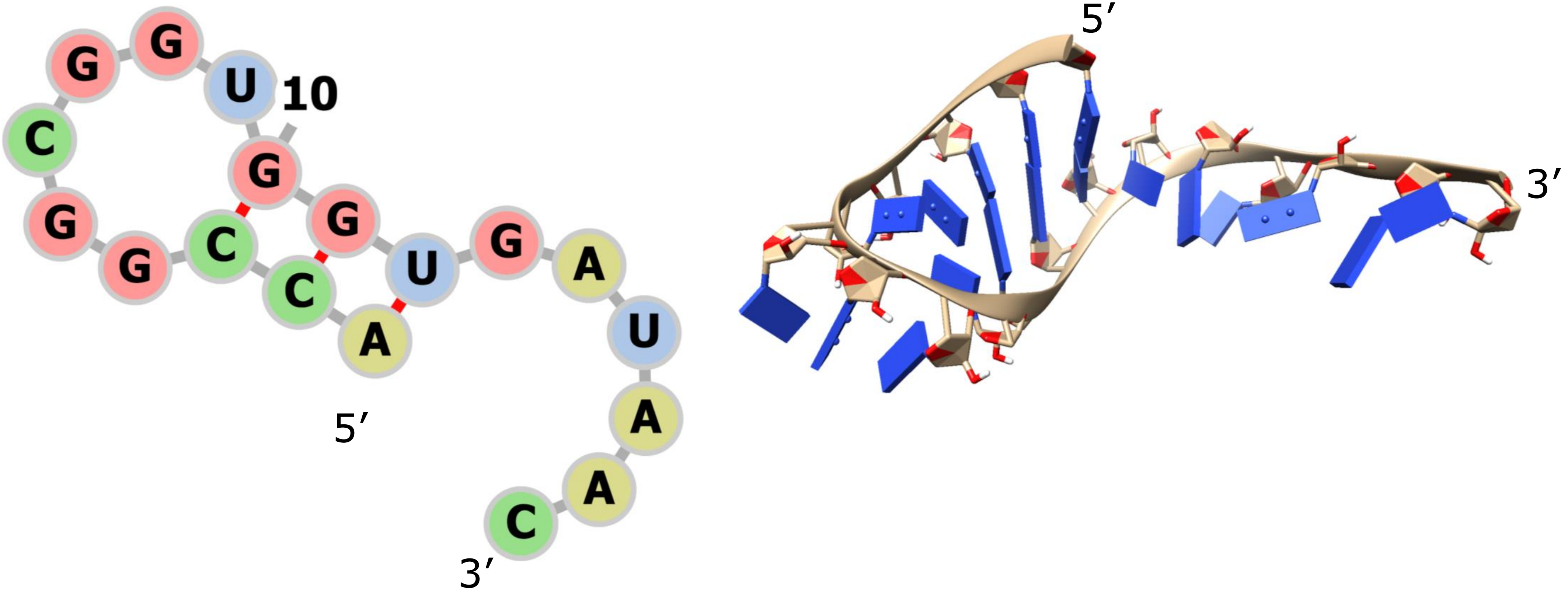

$5A_{bio}$: RNA moiety from SRP of the archaeon “Kocry”,
MFE = -2.48

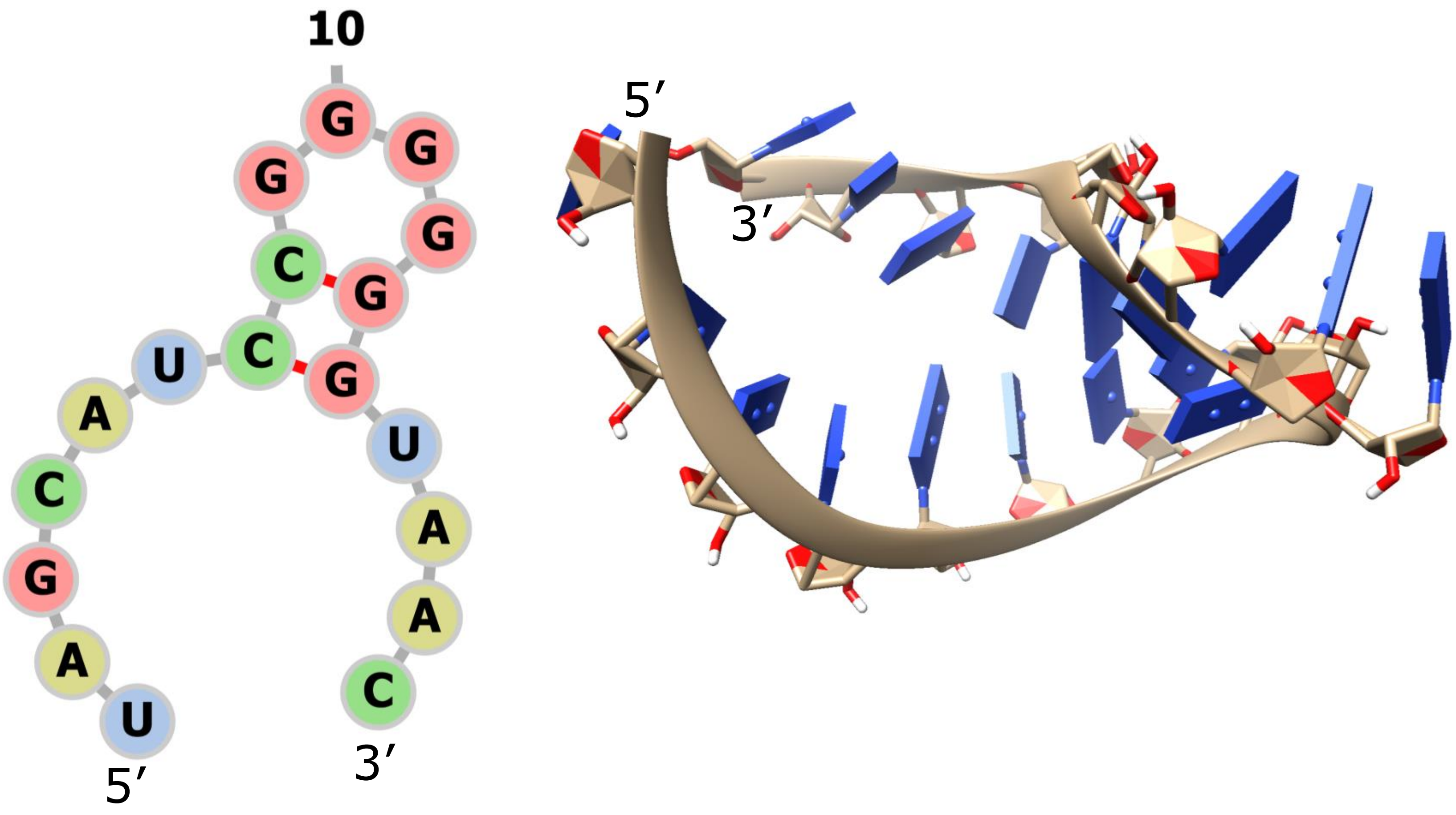

5A$_{ctrl}$: RNA moiety from SRP of the archaeon “Kocry”,
shuffled, MFE = -0.86

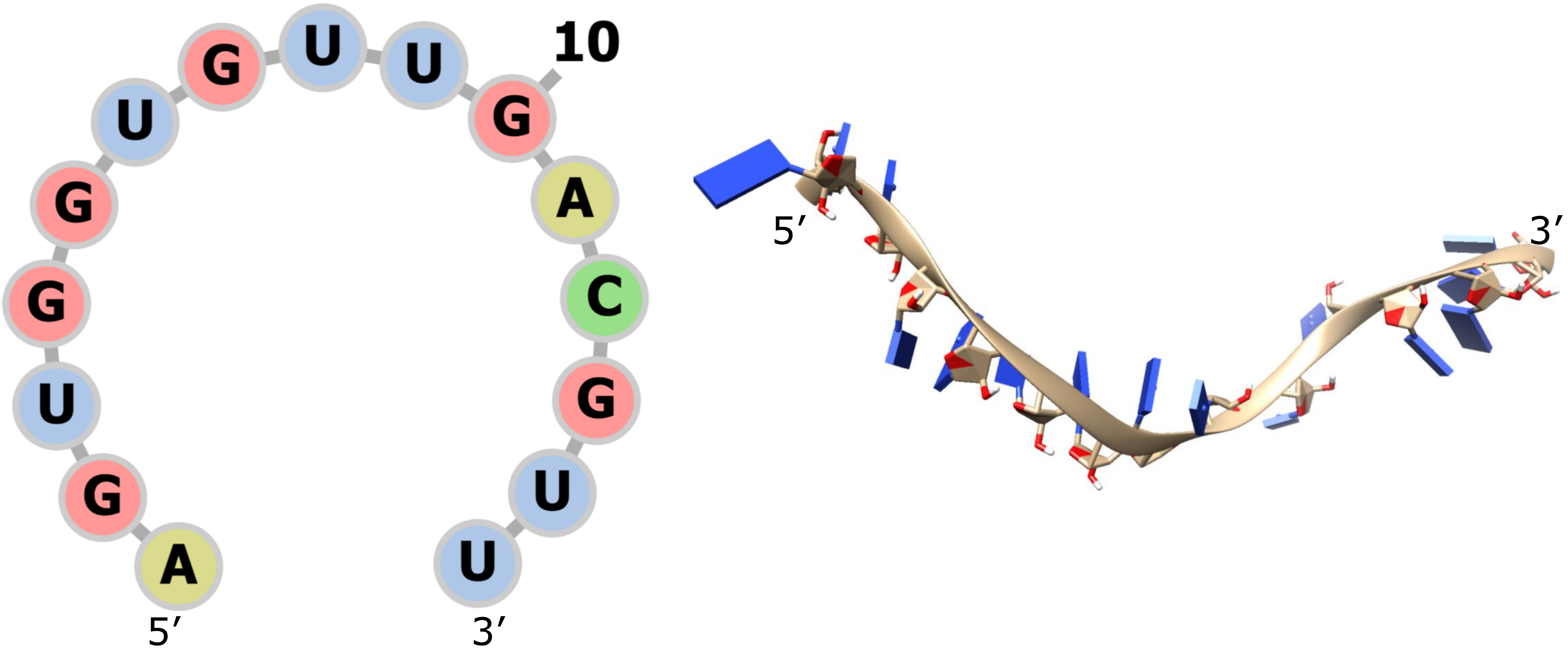

5B$_{bio}$: RNA moiety from long SRP of the bacterium “Basub”,
MFE = 0.0

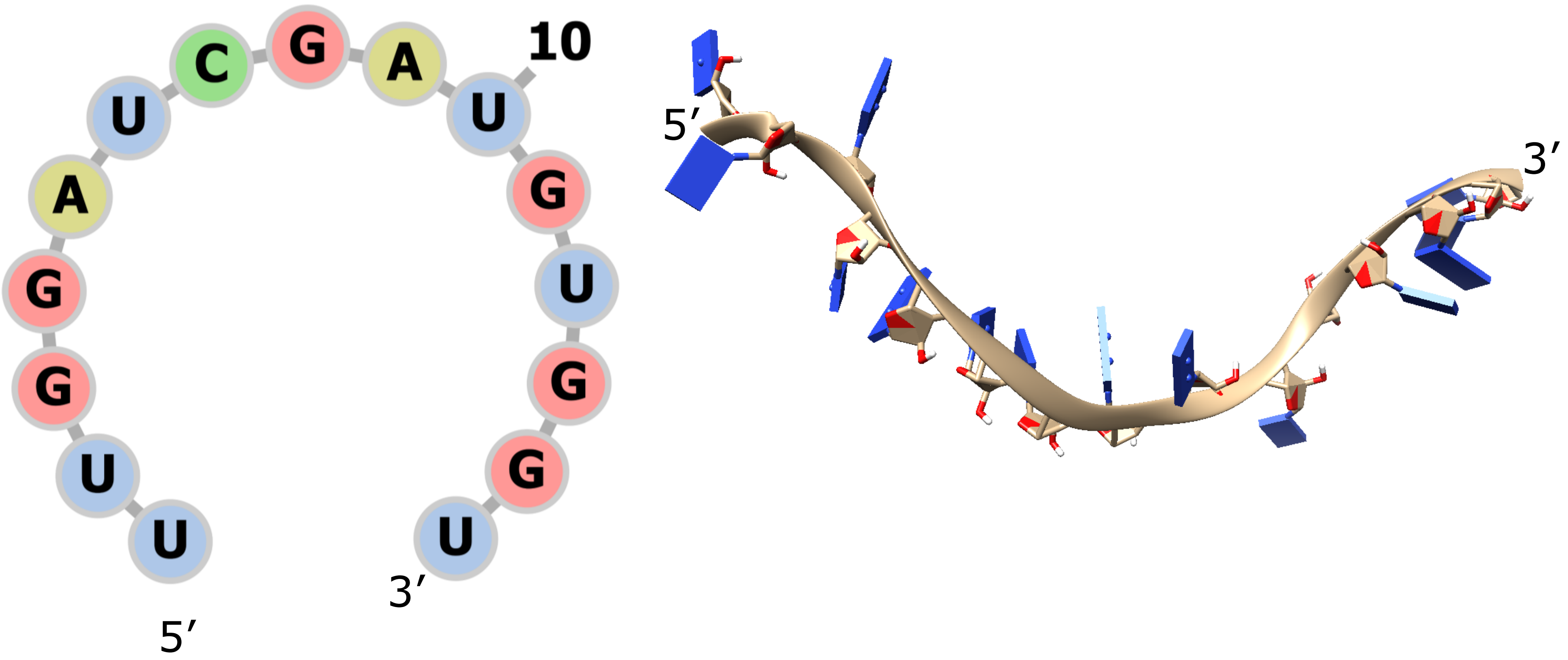

5B$_{ctrl}$: RNA moiety from long SRP of the bacterium “Basub”,
shuffled, MFE = 0.0

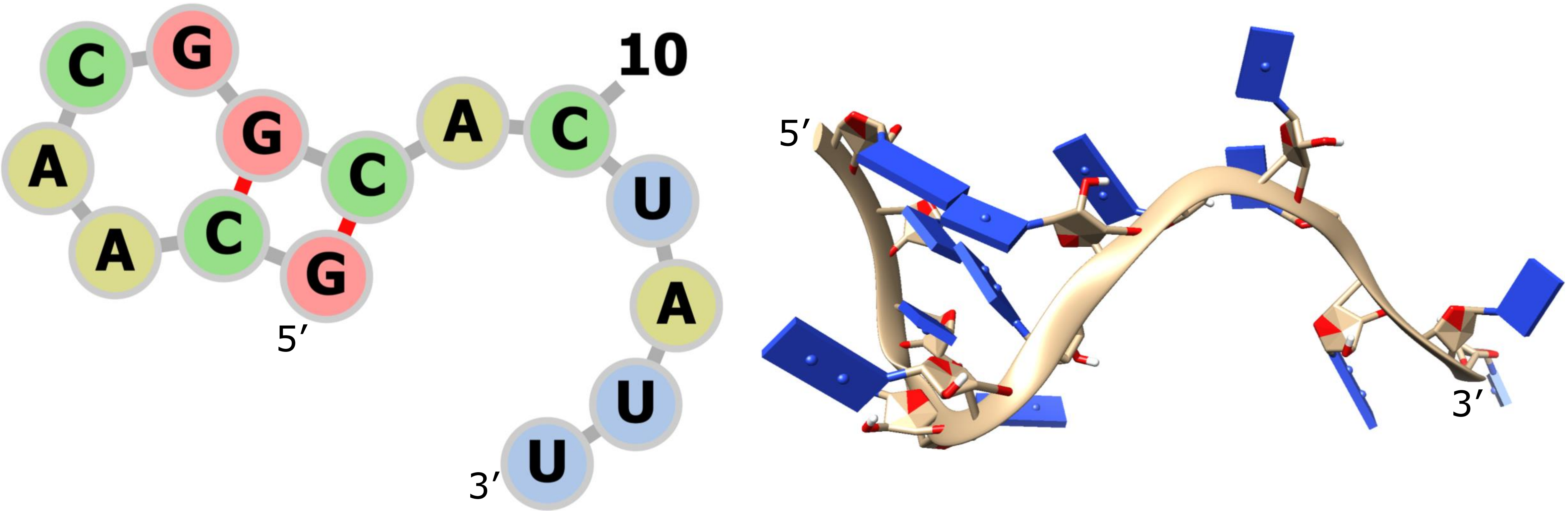

6A$_{bio}$: RNA 6S of the bacterium “Basub”, MFE = -0.25

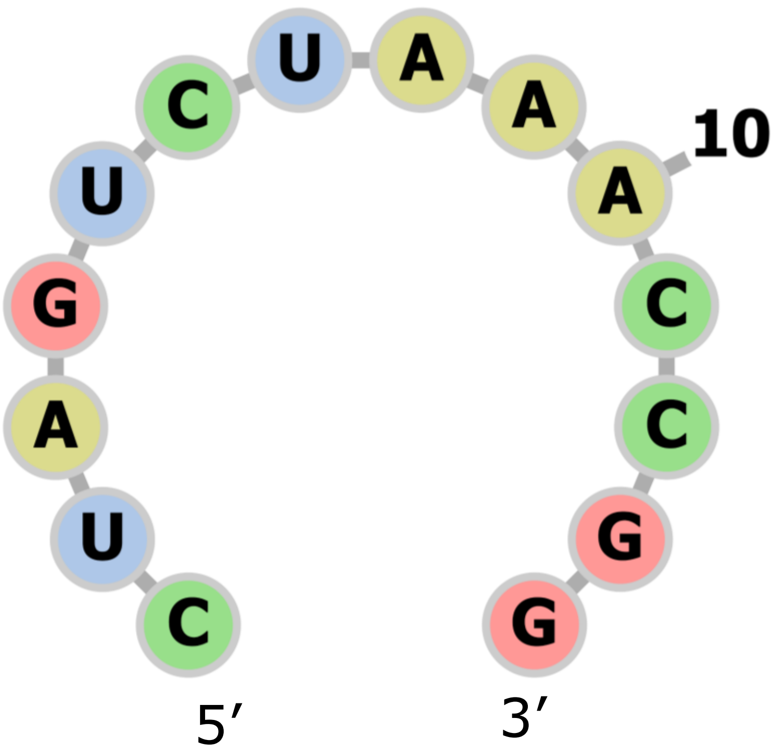

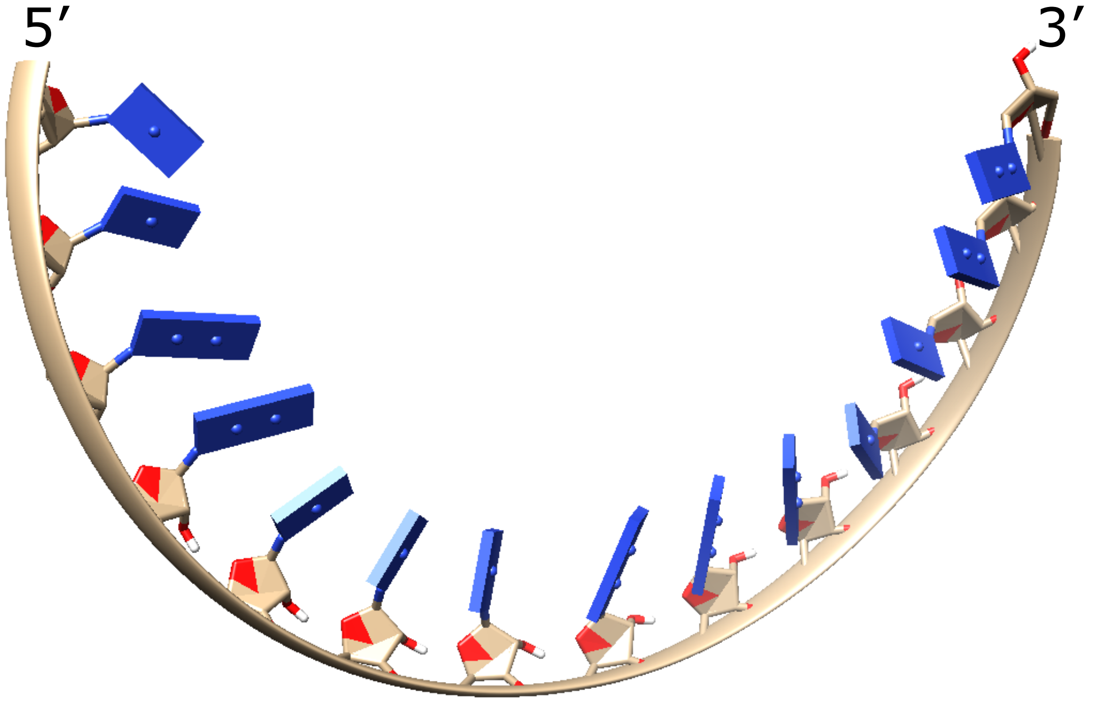

6A$_{ctrl}$: RNA 6S of the bacterium “Basub”, shuffled, MFE = 0.0

The minimum free energy is provided for each RNA, either the biological ones or the corresponding shuffled ones.

7.- tRNA, **RNY**

- A_{bio}.- Of the archaeon “Haqwa”, for Gln-UUG: 2D & 3D.
- A_{ctrl}.- Of the archaeon “Haqwa”, for Gln-UUG, shuffled: 2D & 3D.
- B_{bio}.- Of the archaeon “Thgam”, for Asn-GUU: 2D & 3D.
- B_{ctrl}.- Of the archaeon “Thgam”, for Asn-GUU, shuffled: 2D & 3D.
- C_{bio}.- Of the archaeon “Thsib”, for Asn-GUU: 2D & 3D.
- C_{ctrl}.- Of the archaeon “Thsib”, for Asn-GUU, shuffled: 2D & 3D.
- D_{bio}.- Of the bacterium “Bobur”, for Gln-UUG: 2D & 3D.
- D_{ctrl}.- Of the bacterium “Bobur”, for Gln-UUG, shuffled: 2D & 3D.
- E_{bio}.- Of the bacterium “Derad”, for Cys-GCA: 2D & 3D.
- E_{ctrl}.- Of the bacterium “Derad”, for Cys-GCA, shuffled: 2D & 3D.
- F_{bio}.- Of the bacterium “Derad”, for Gln-CUG: 2D & 3D.
- F_{ctrl}.- Of the bacterium “Derad”, for Gln-CUG, shuffled: 2D & 3D.
- G_{bio}.- Of the bacterium “Derad”, for Glu-UUC: 2D & 3D.
- G_{ctrl}.- Of the bacterium “Derad”, for Glu-UUC, shuffled: 2D & 3D.
- H_{bio}.- Of the bacterium “Derad”, for Gly-UCC: 2D & 3D.
- H_{ctrl}.- Of the bacterium “Derad”, for Gly-UCC, shuffled: 2D & 3D.
- I_{bio}.- Of the bacterium “Peubi”, for Gly-GCC: 2D & 3D.
- I_{ctrl}.- Of the bacterium “Peubi”, for Gly-GCC, shuffled: 2D & 3D.
- J_{bio}.- Of the bacterium “SagA”, for Asn-GUU: 2D & 3D.
- J_{ctrl}.- Of the bacterium “SagA”, for Asn-GUU, shuffled: 2D & 3D.
- K_{bio}.- Of the bacterium “SagA”, for Thr-GGU: 2D & 3D.
- K_{ctrl}.- Of the bacterium “SagA”, for Thr-GGU, shuffled: 2D & 3D.
- L_{bio}.- Of the bacterium “Thmar”, for Phe-gaa: 2D & 3D.
- L_{ctrl}.- Of the bacterium “Thmar”, for Phe-gaa, shuffled: 2D & 3D.

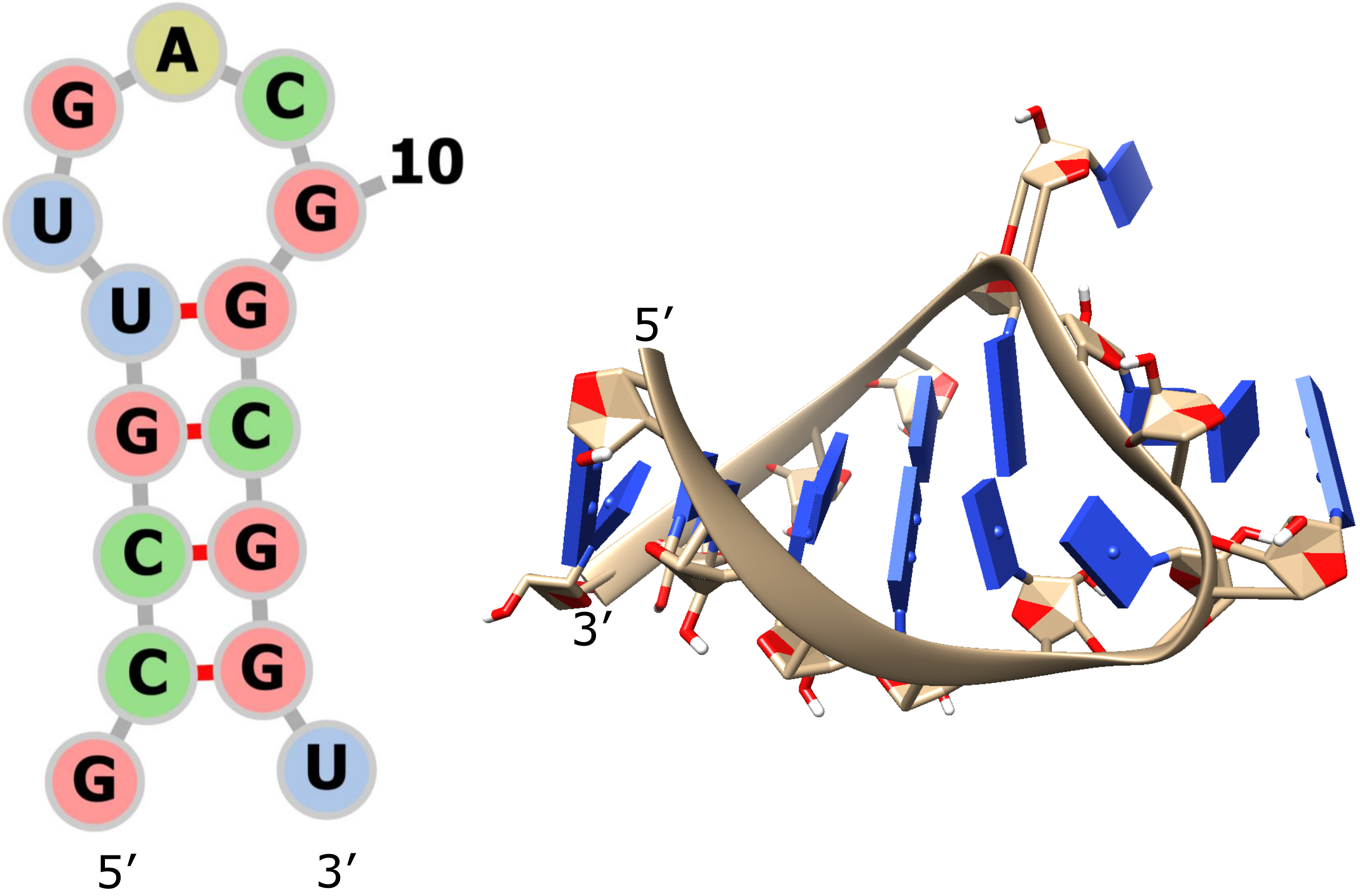

7A$_{bio}$: tRNA from the archaeon “Haqwa”,
for Gln, UUG, MFE = -4.87

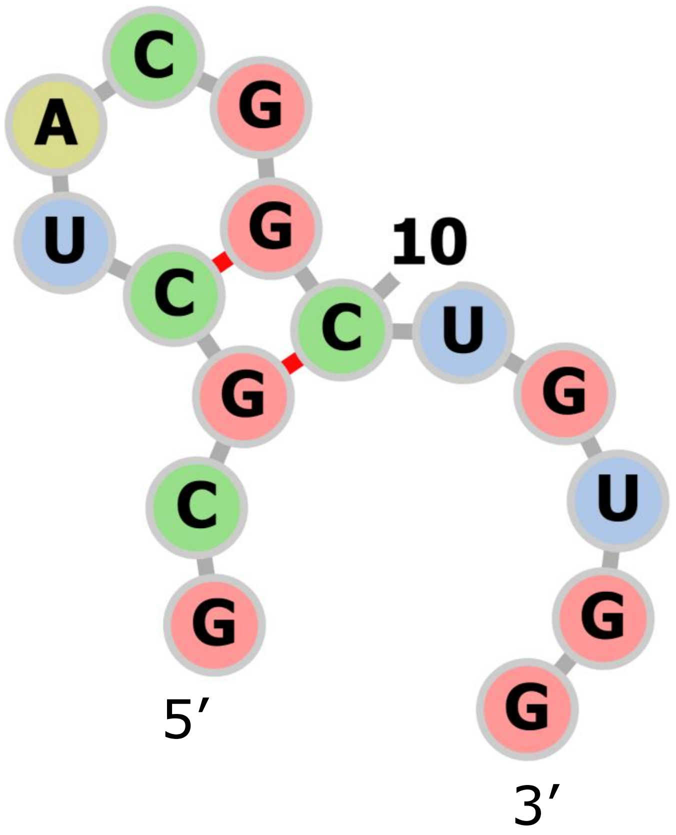

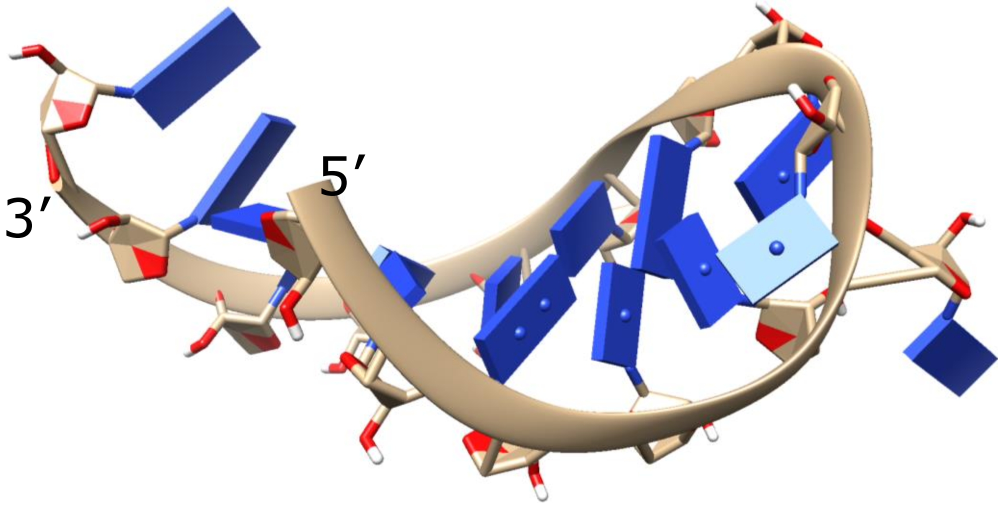

7A$_{\mathbf{ctrl}}$: tRNA from the archaeon “Haqwa”,
for Gln, UUG, shuffled, MFE = -4.14

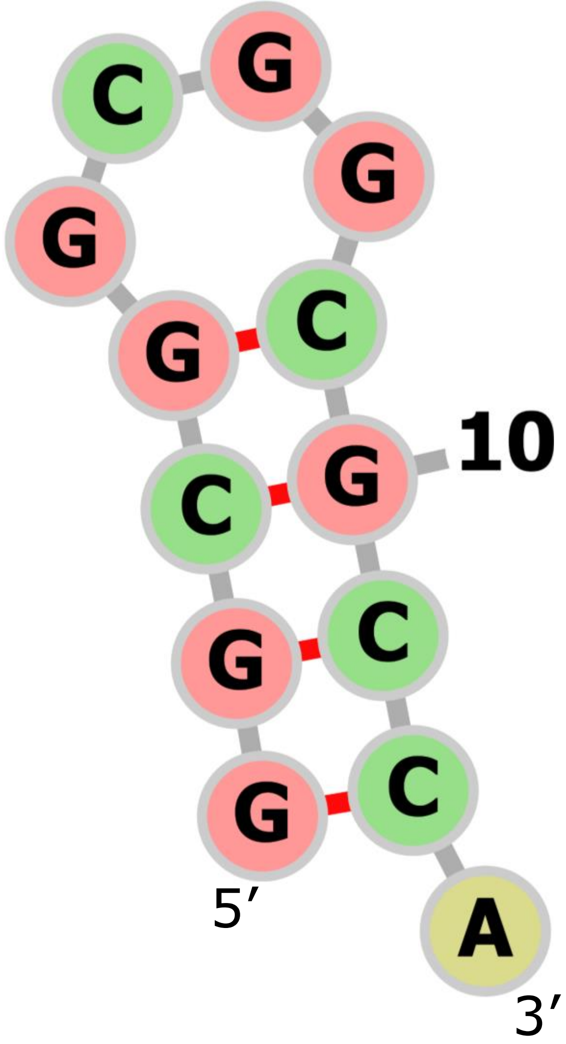

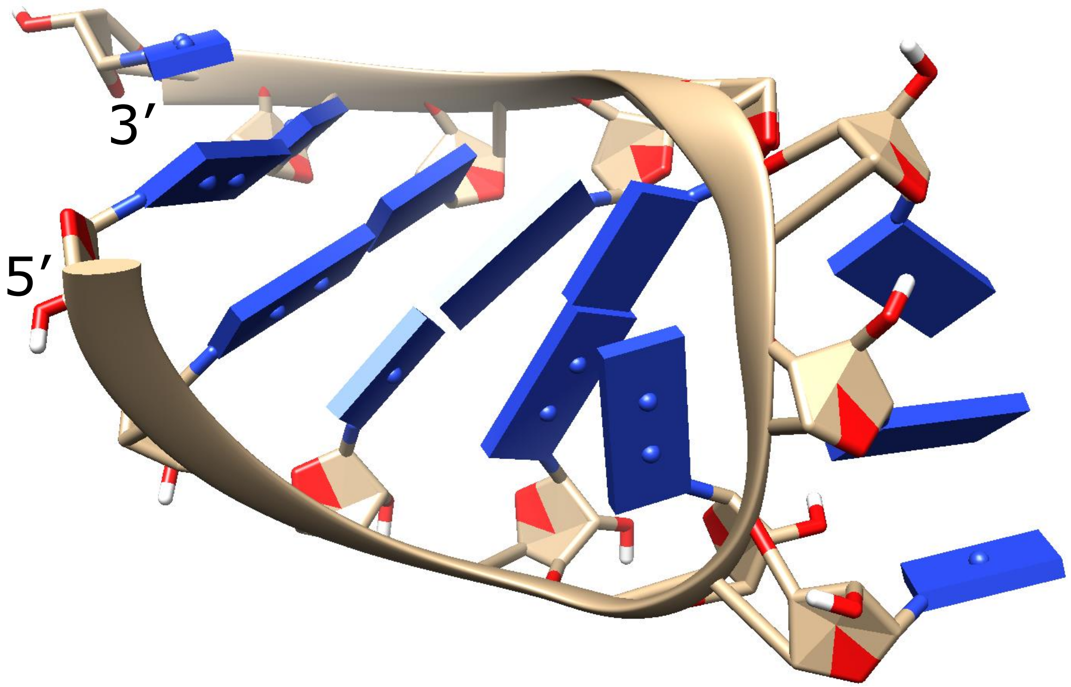

$7B_{bio}$: tRNA from the archaeon “Thgam”,
for Asn, GUU, MFE = -5.69

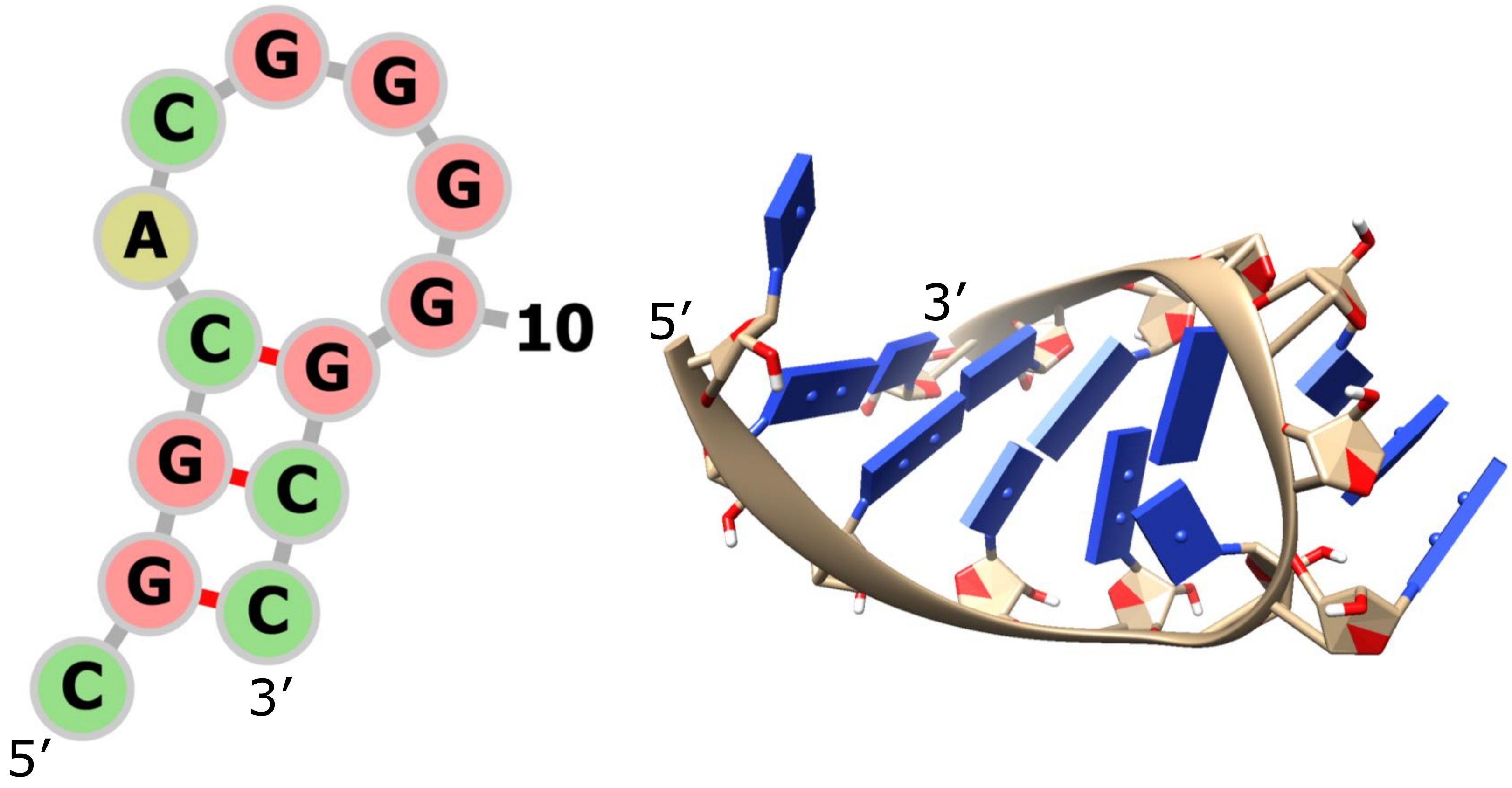

7B$_{ctrl}$: tRNA from the archaeon “Thgam”, for Asn, GUU, shuffled, MFE = -2.45

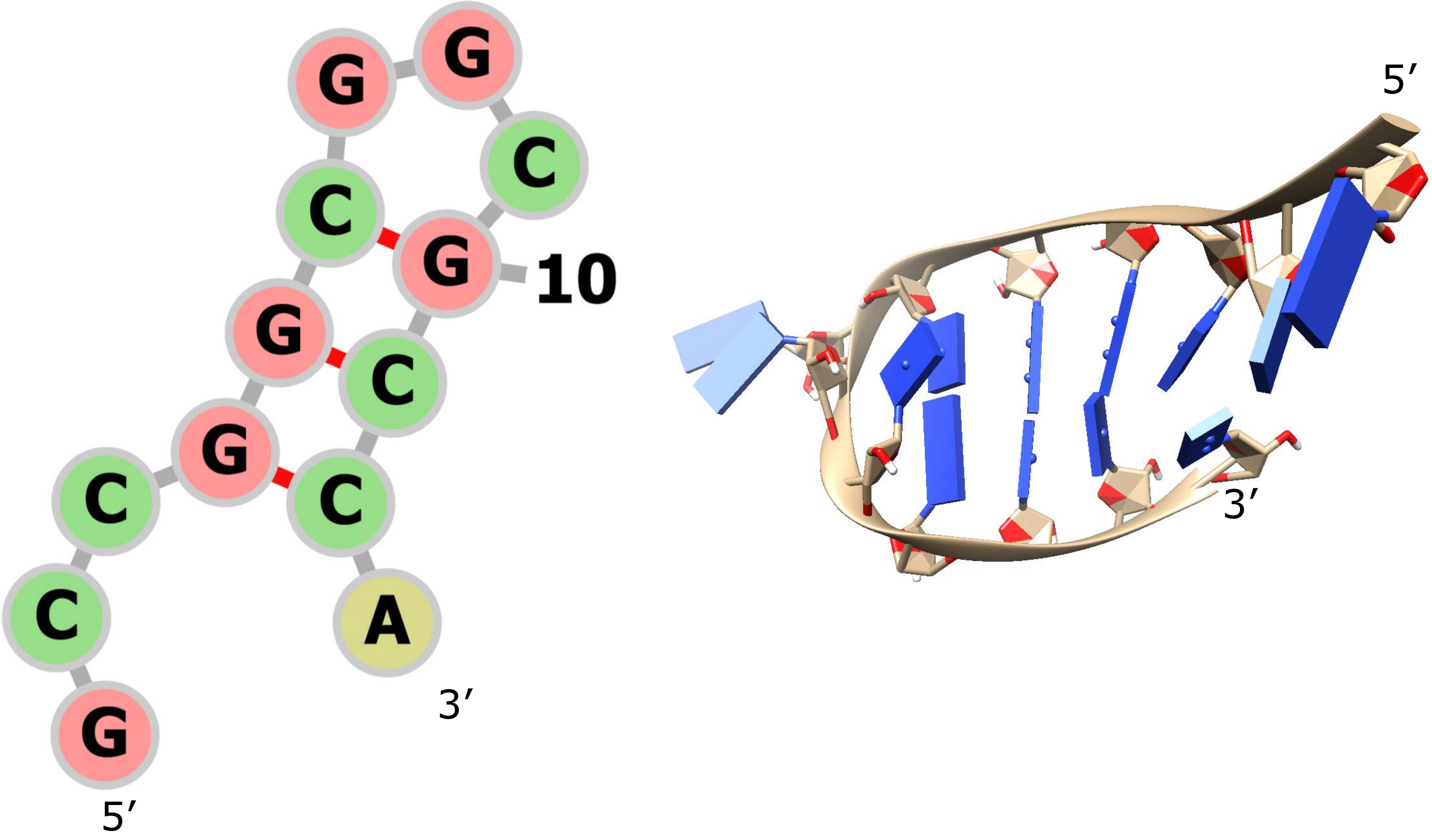

7C$_{bio}$: tRNA from the archaeon “Thsib”,
for Asn, GUU, MFE = -2.90

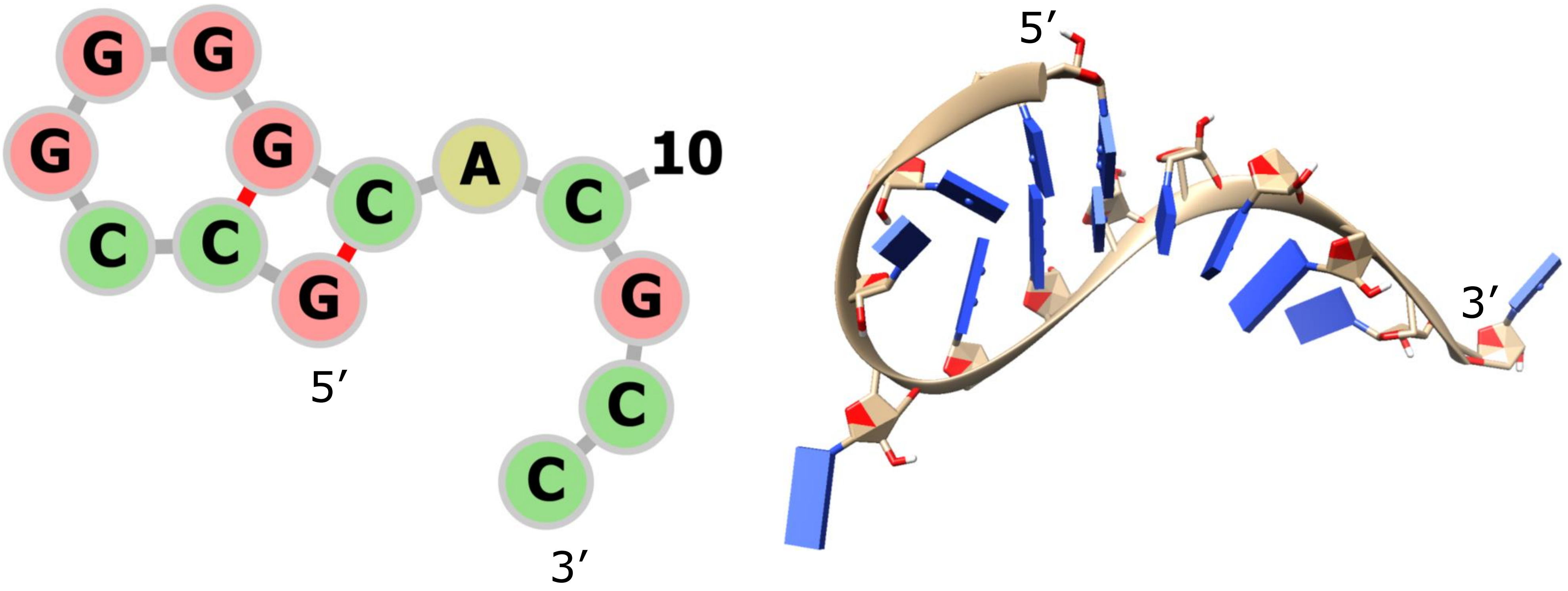

7C$_{ctrl}$: tRNA from the archaeon “Thsib”,
for Asn, GUU, shuffled, MFE = -2.08

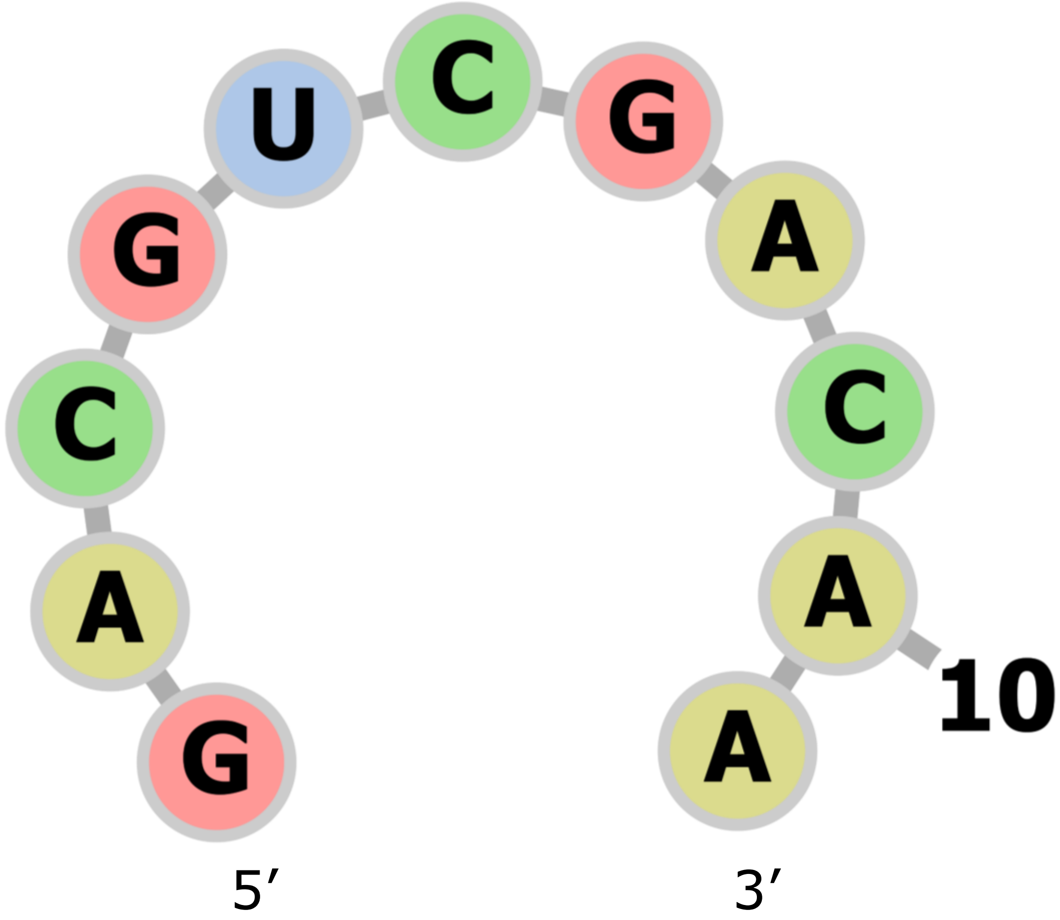

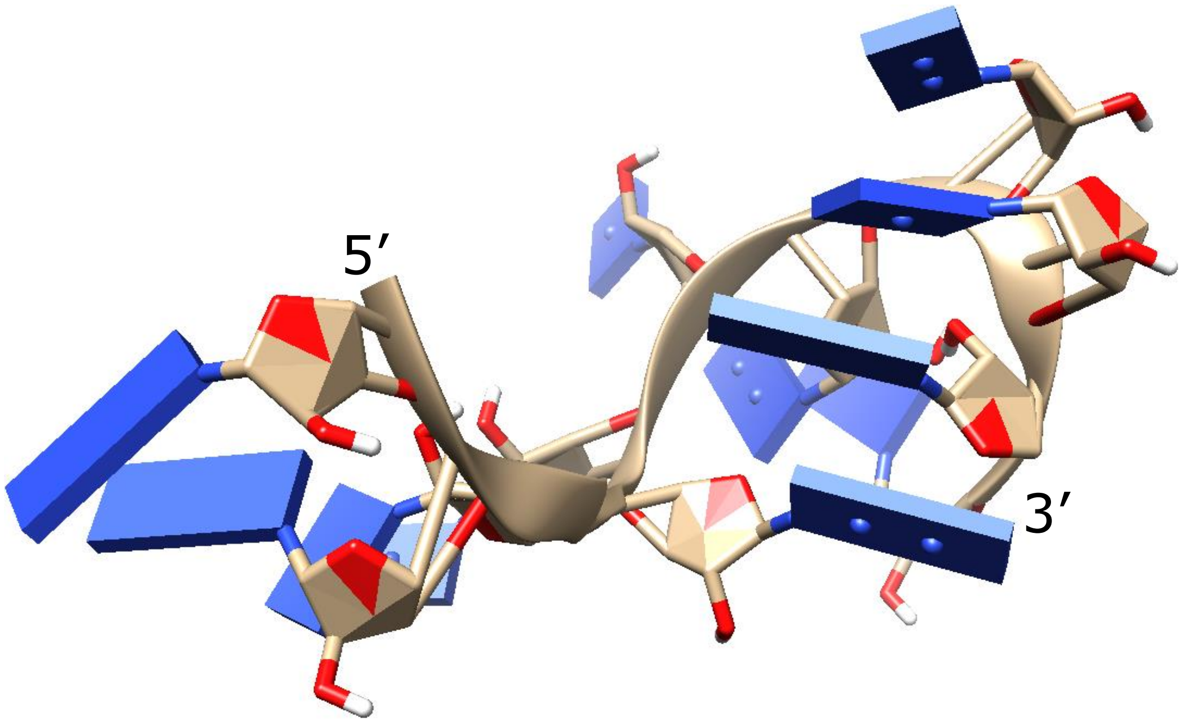

7D$_{bio}$: tRNA from the bacterium “Bobur”,
for Gln, UUG, MFE = 0.0

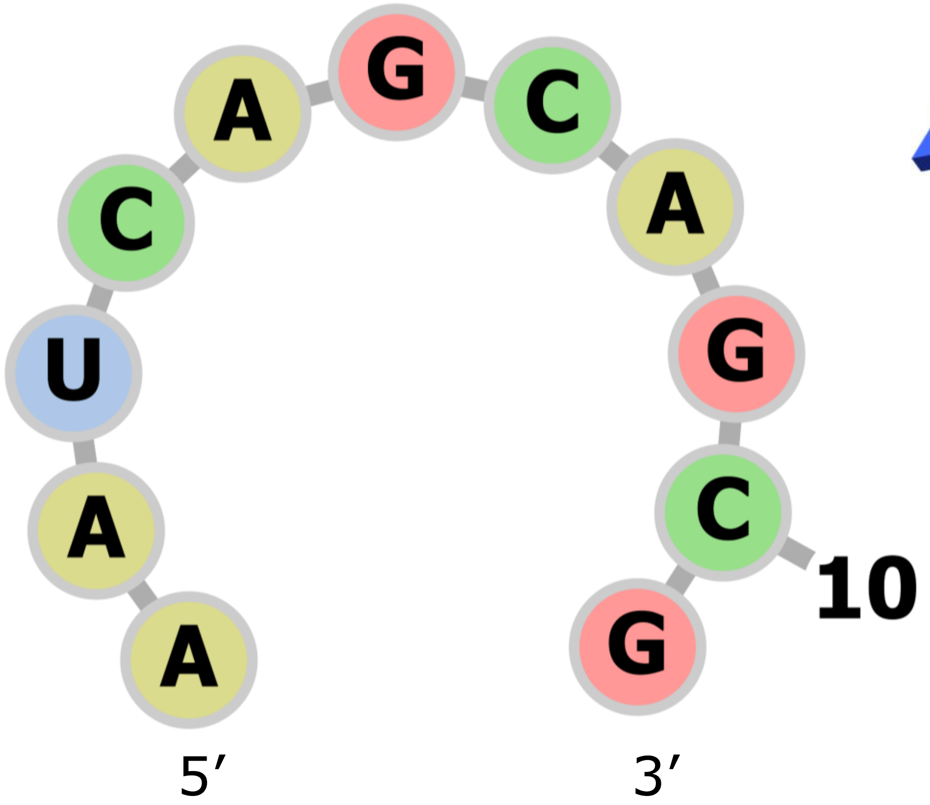

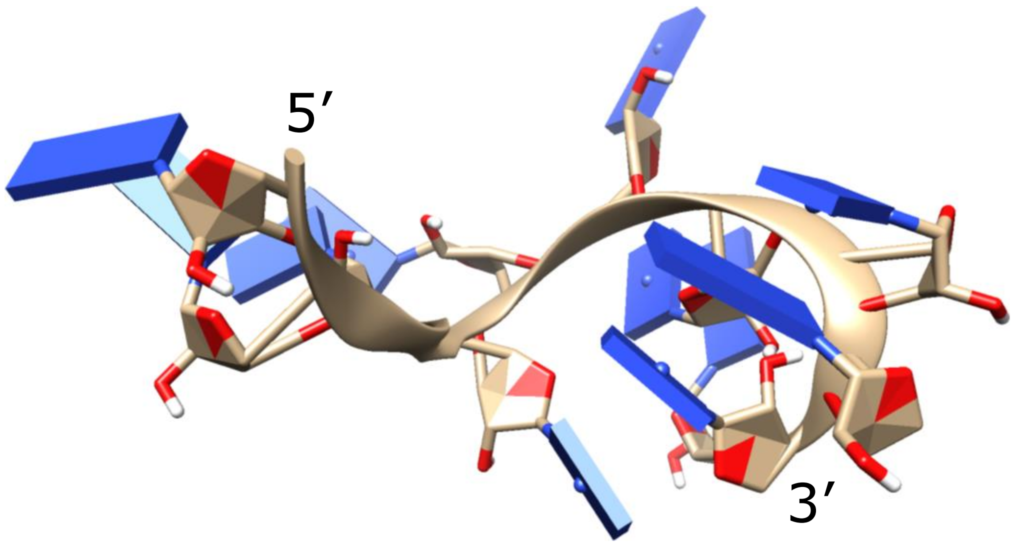

7D$_{ctrl}$: tRNA from the bacterium “Bobur”,
for Gln, UUG, shuffled, MFE = 0.0

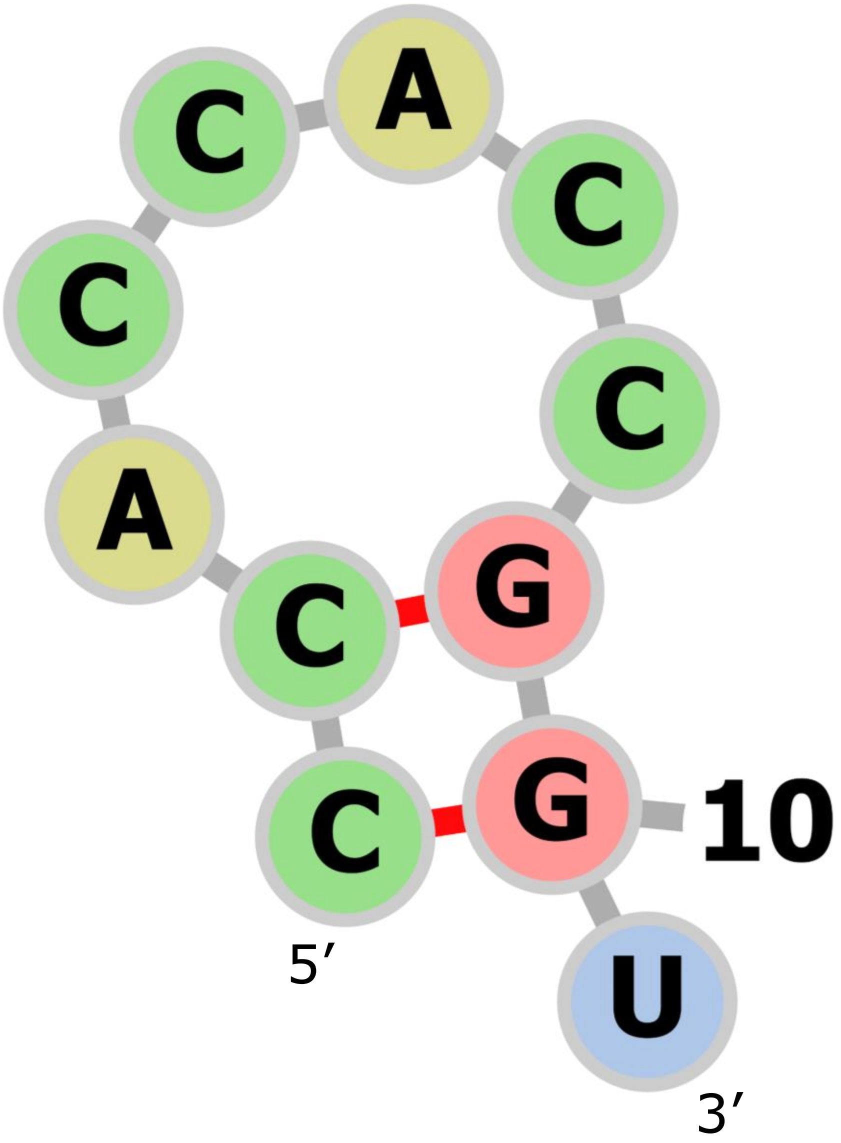

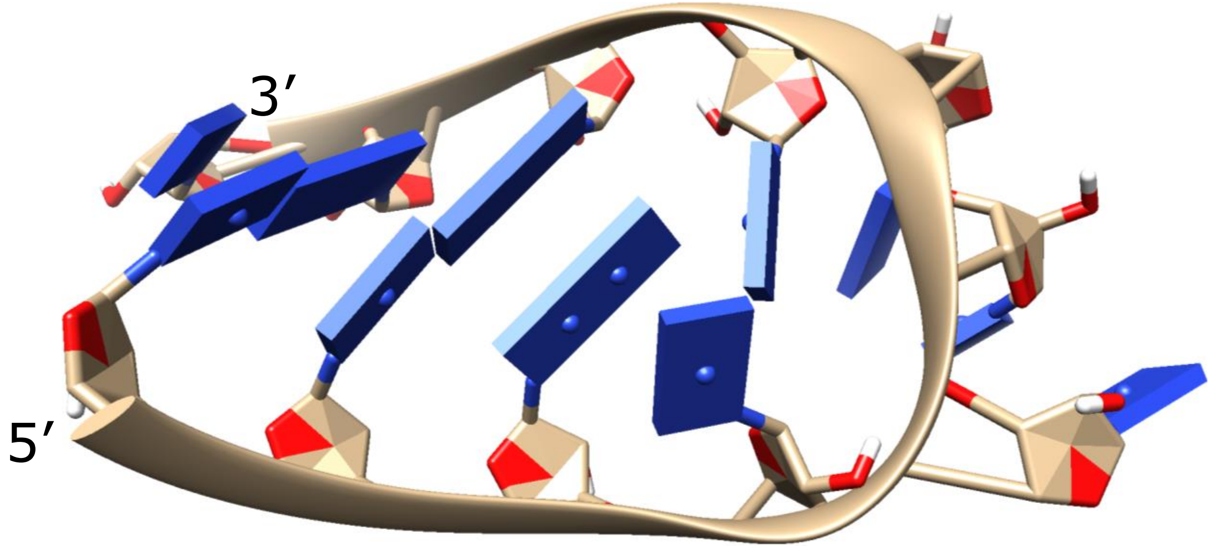

7E$_{bio}$: tRNA from the bacterium “Derad”,
for Cys, GCA, MFE = -0.80

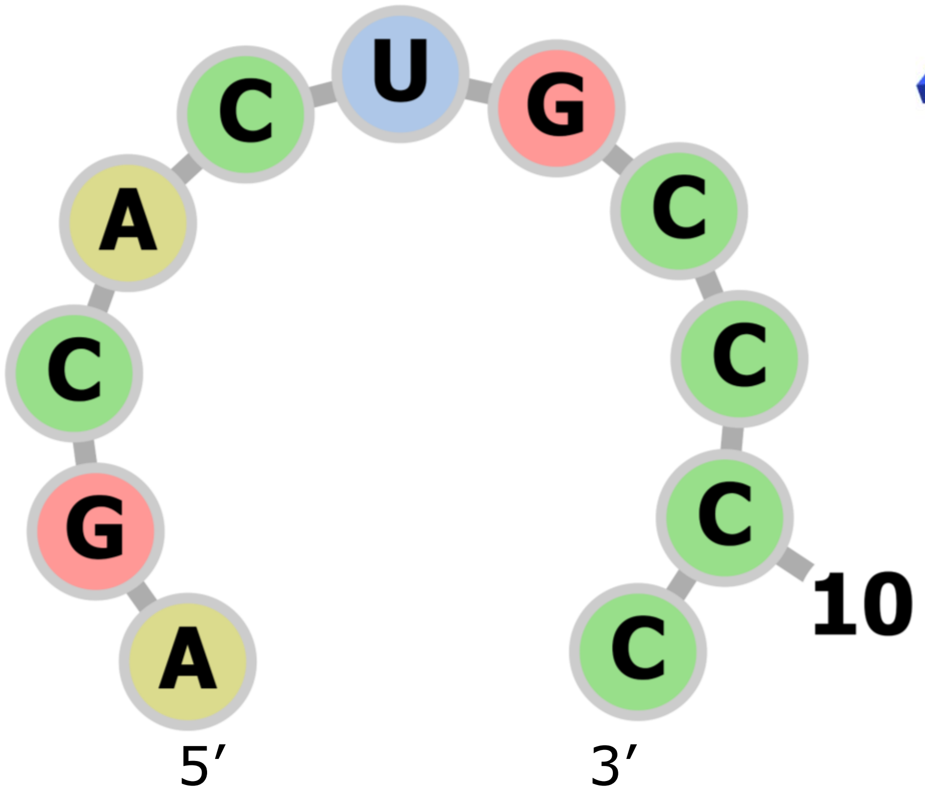

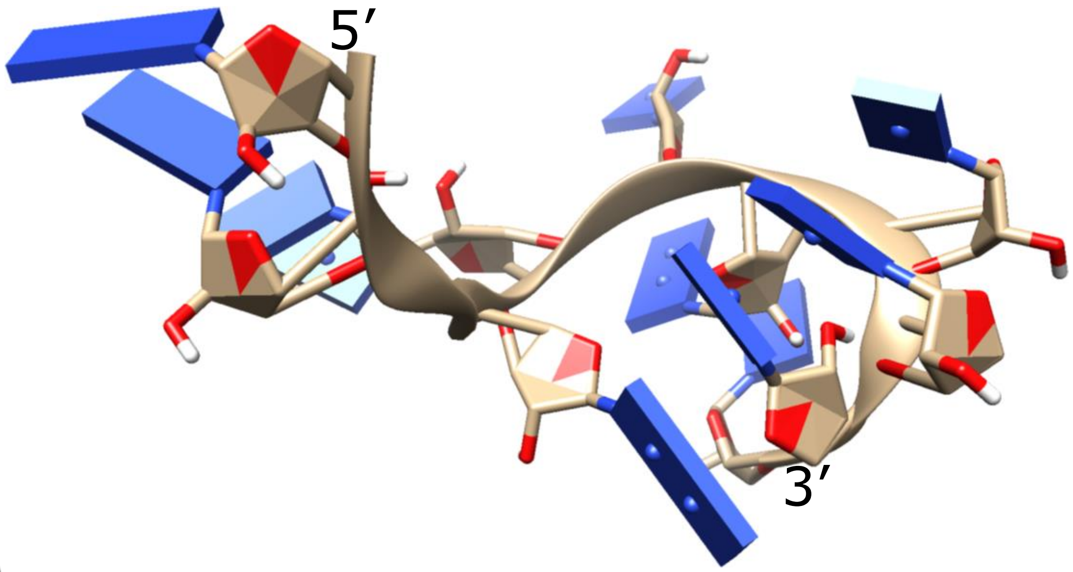

$7E_{trl}$: tRNA from the bacterium “Derad”,
for Cys, GCA, shuffled, MFE = 0.0

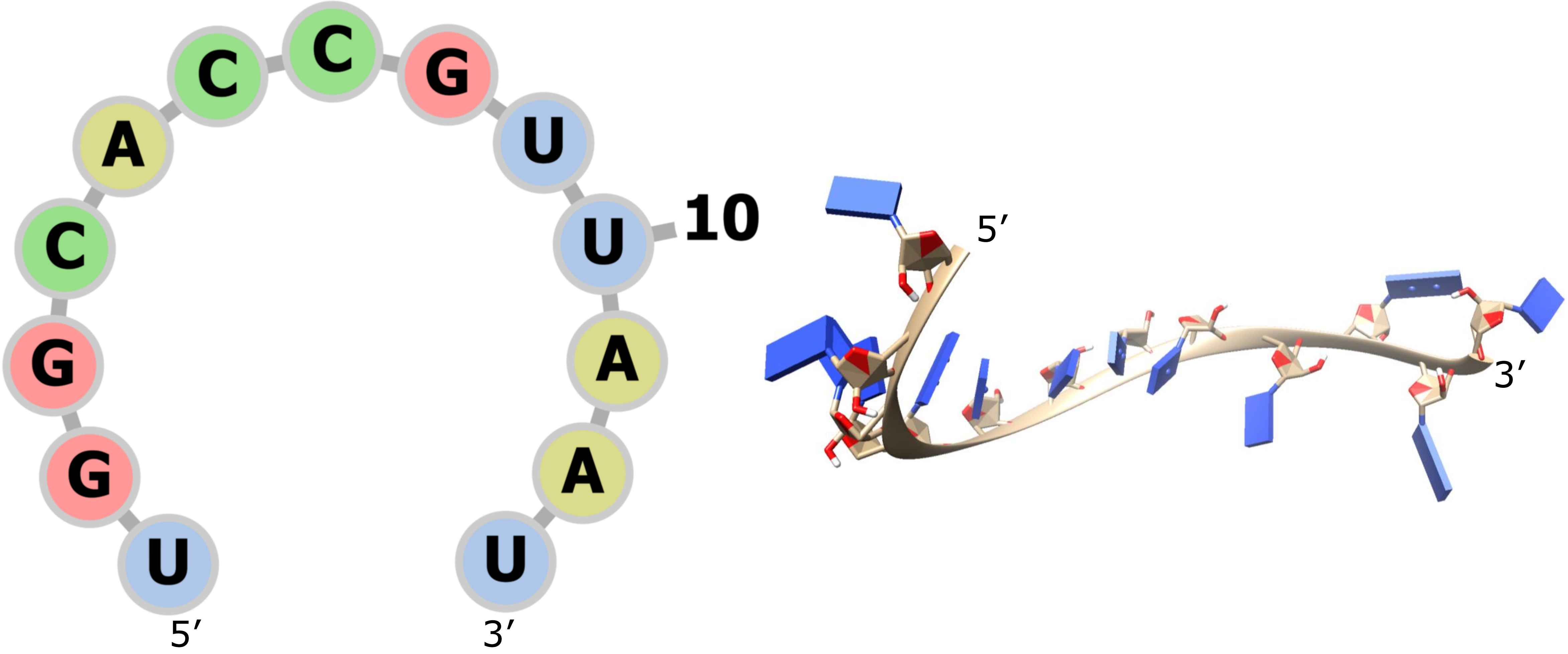

7F$_{bio}$: tRNA from the bacterium "Derad",
for Gln, CUG, MFE = 0.0

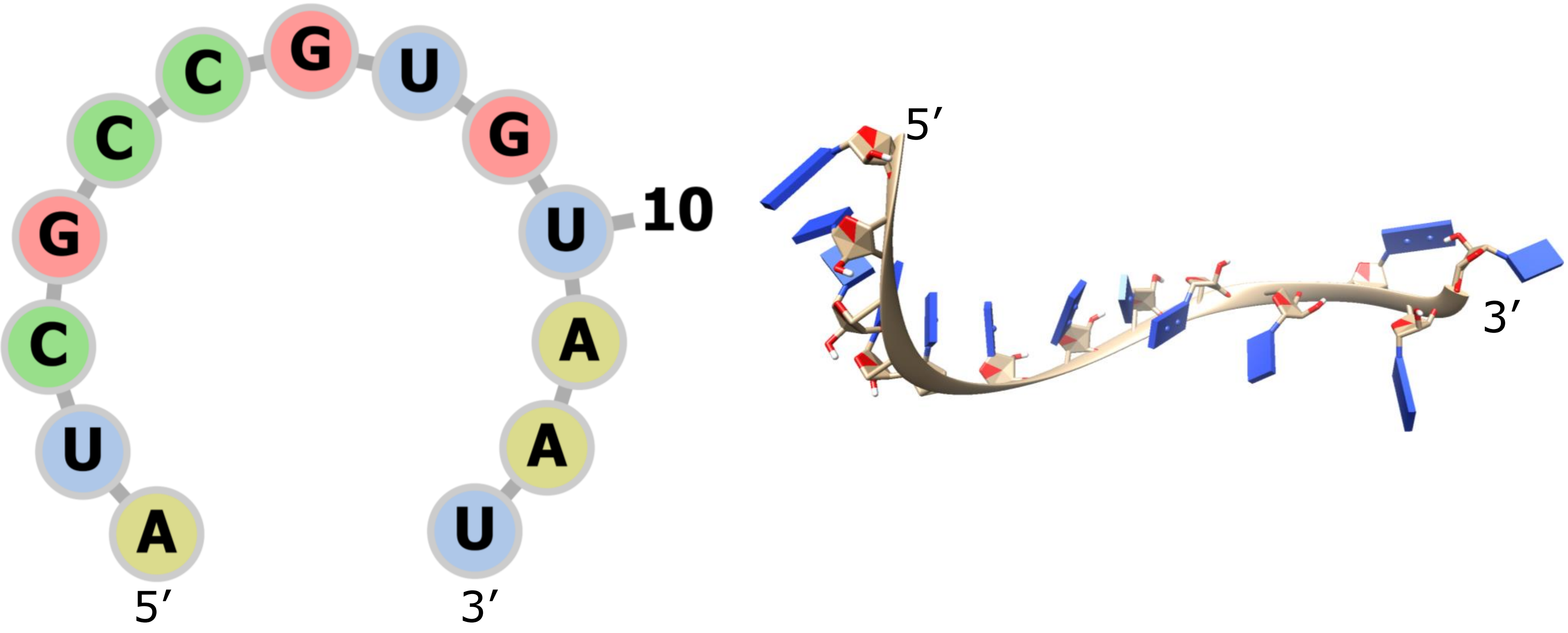

7F$_{ctrl}$: tRNA from the bacterium “Derad”,
for Gln, CUG shuffled, MFE = 0.0

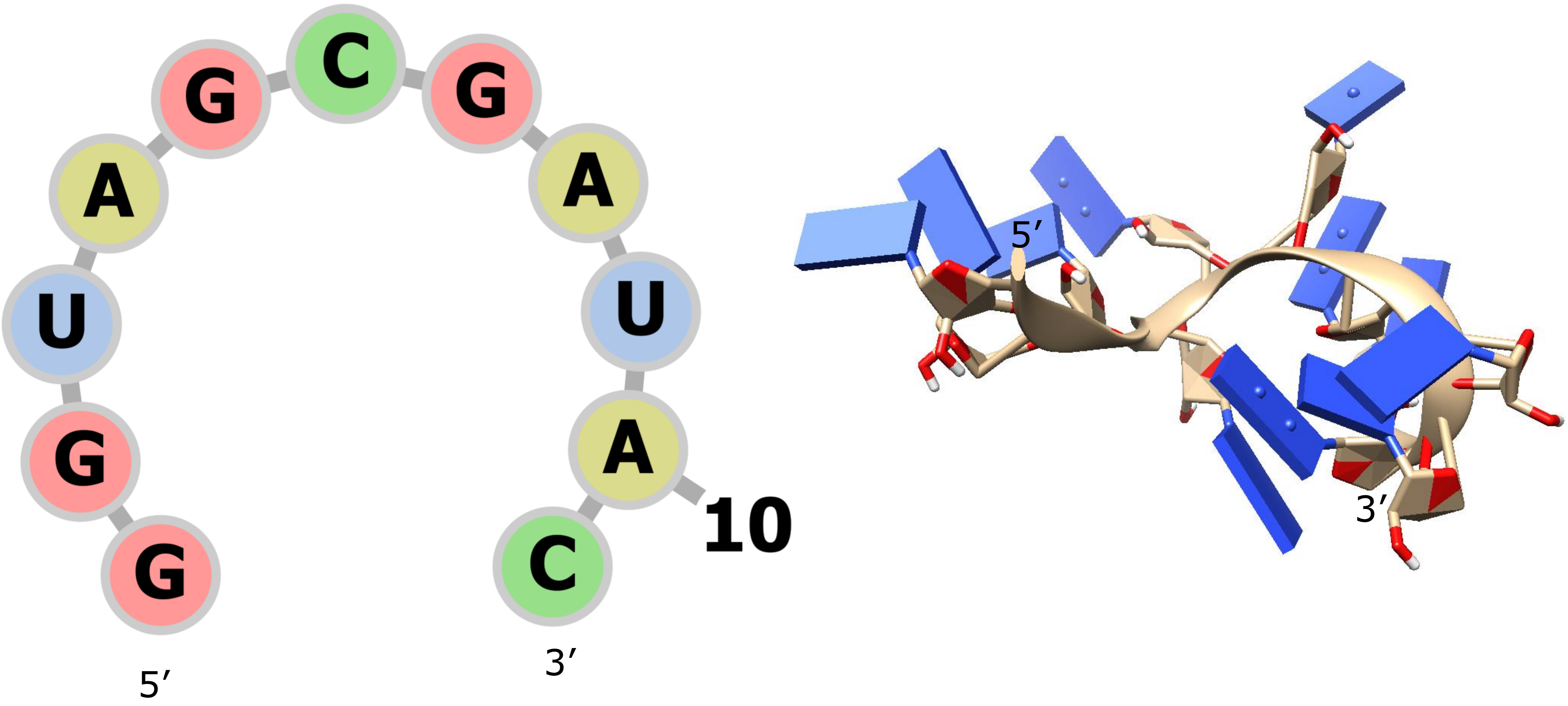

7G$_{bio}$: tRNA from the bacterium “Derad”,
for Glu, UUC, MFE = 0.0

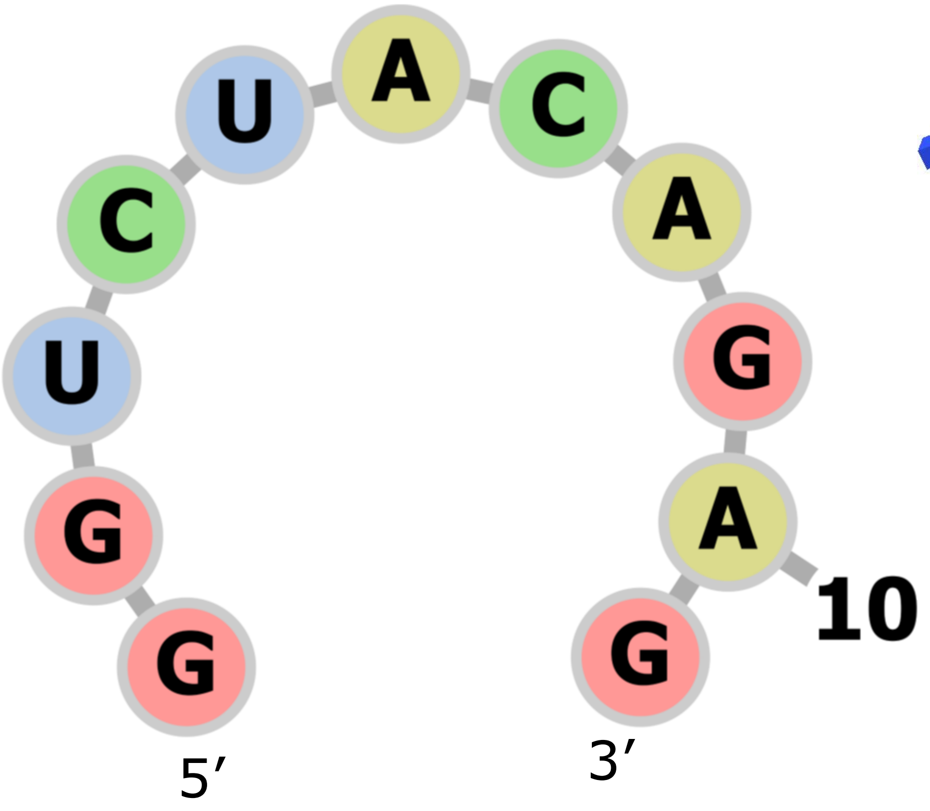

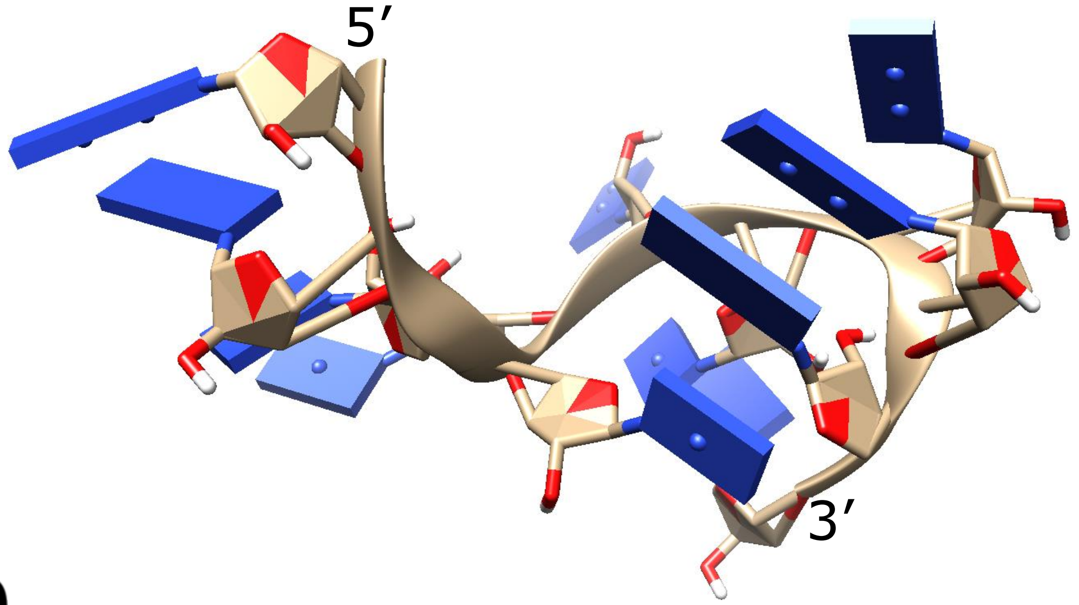

7G$_{ctrl}$: tRNA from the bacterium “Derad”,
for Glu, UUC, shuffled, MFE = 0.0

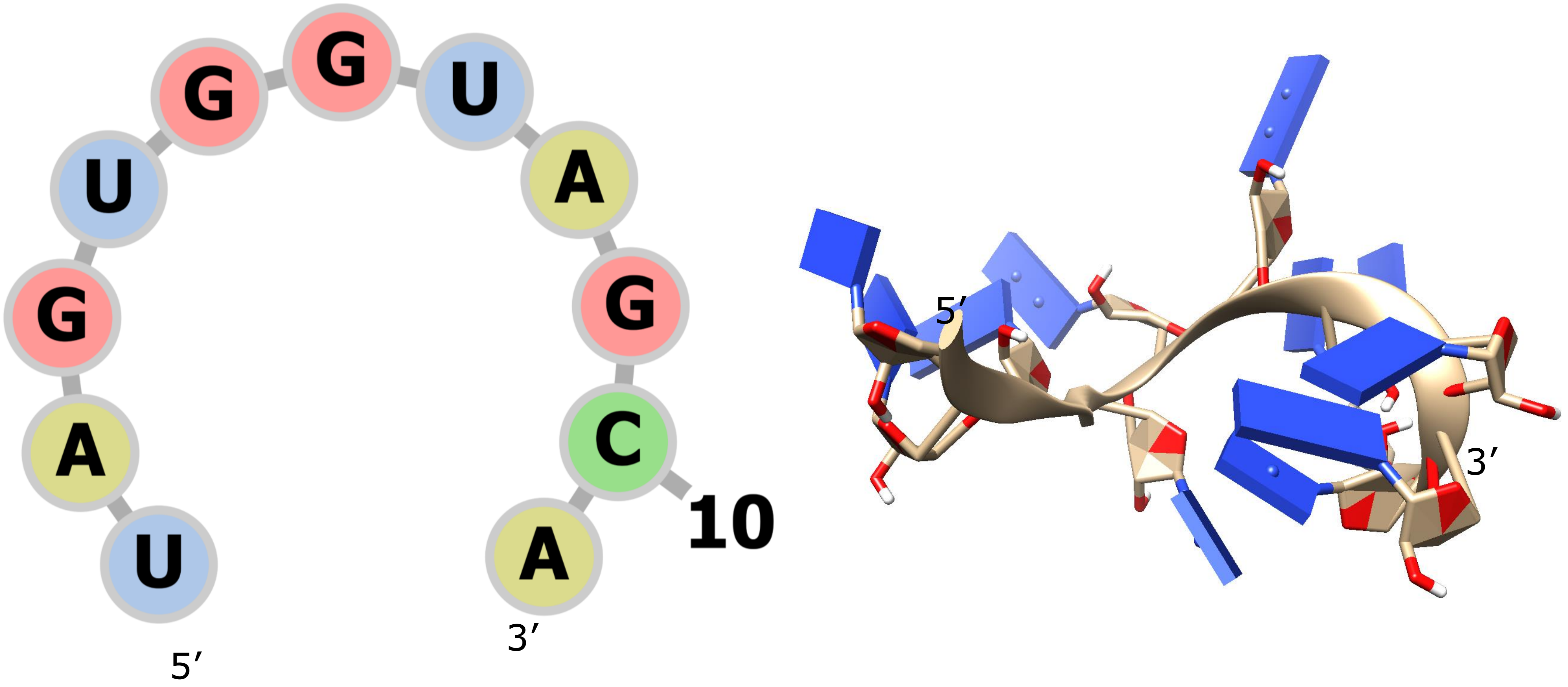

7H$_{bio}$: tRNA from the bacterium “Derad”,
for Gly, UCC, MFE = 0.0

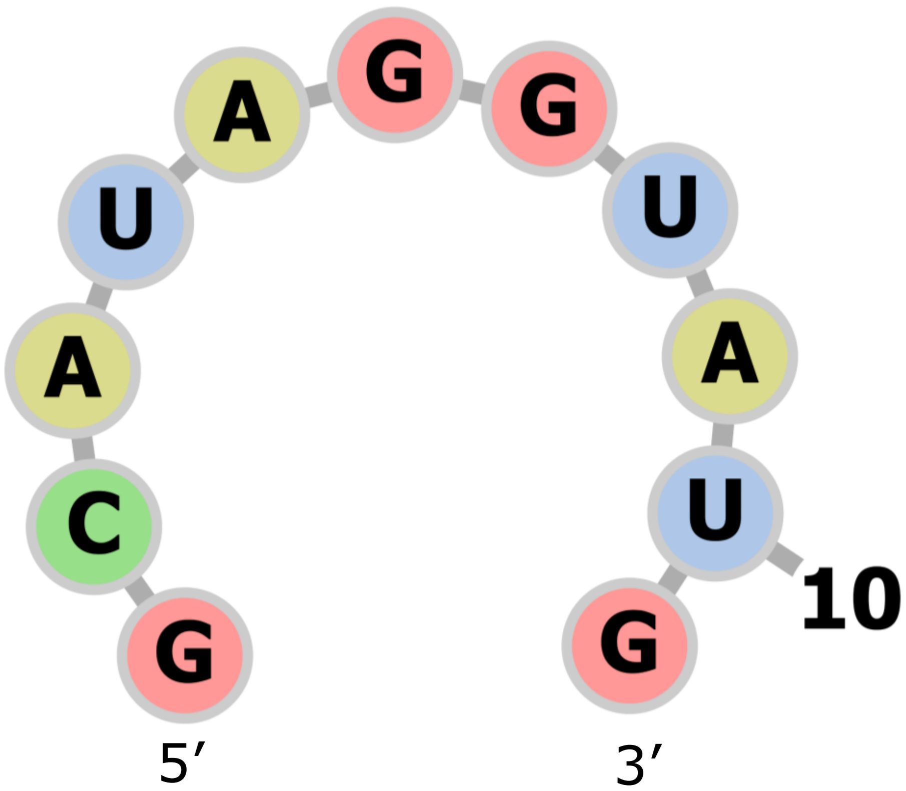

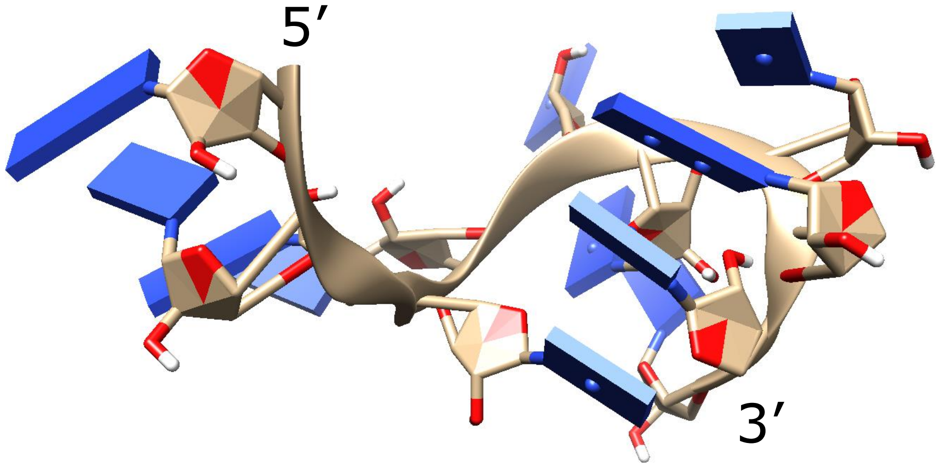

7H$_{ctrl}$: tRNA from the bacterium “Derad”, for Gly, UCC, shuffled, MFE = 0.0

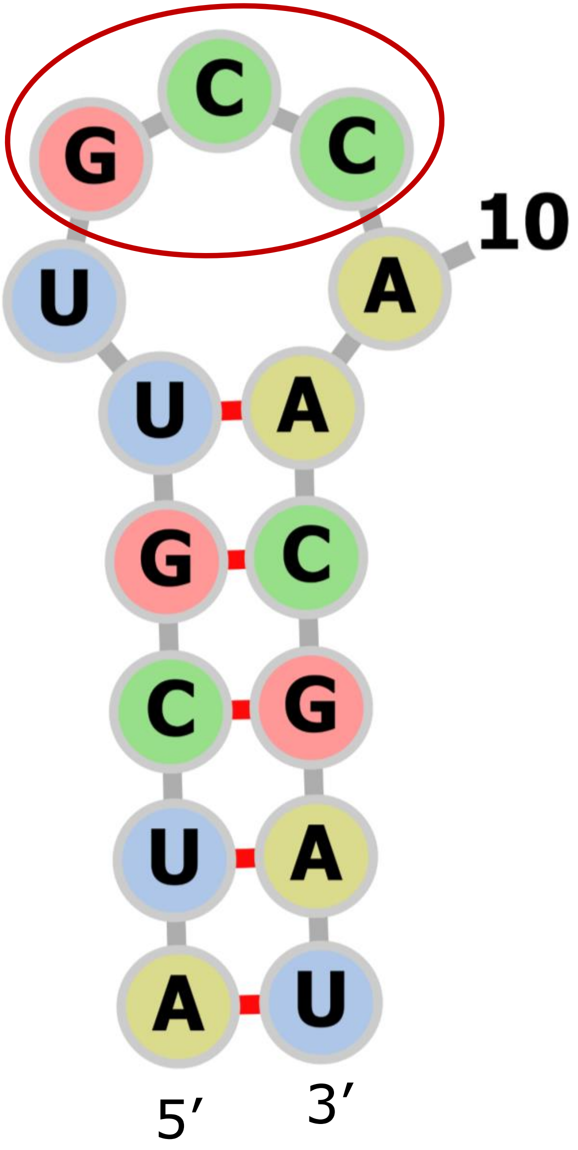

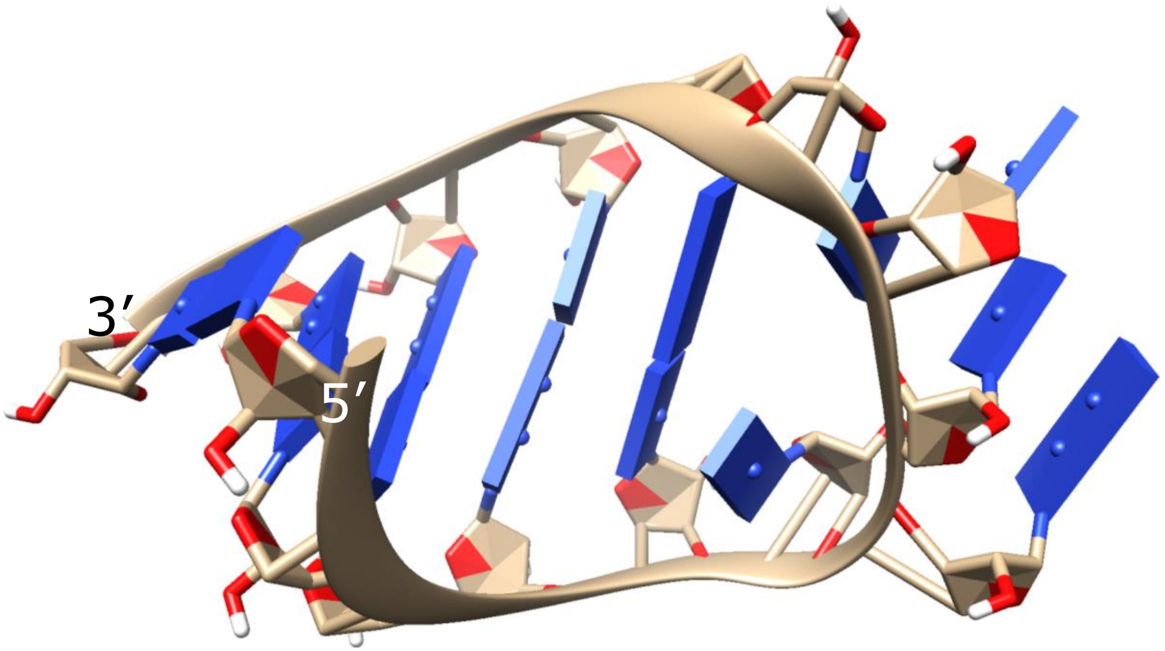

7I$_{bio}$: tRNA from the bacterium "Peubi",
for Gly, GCC, MFE = -2.97

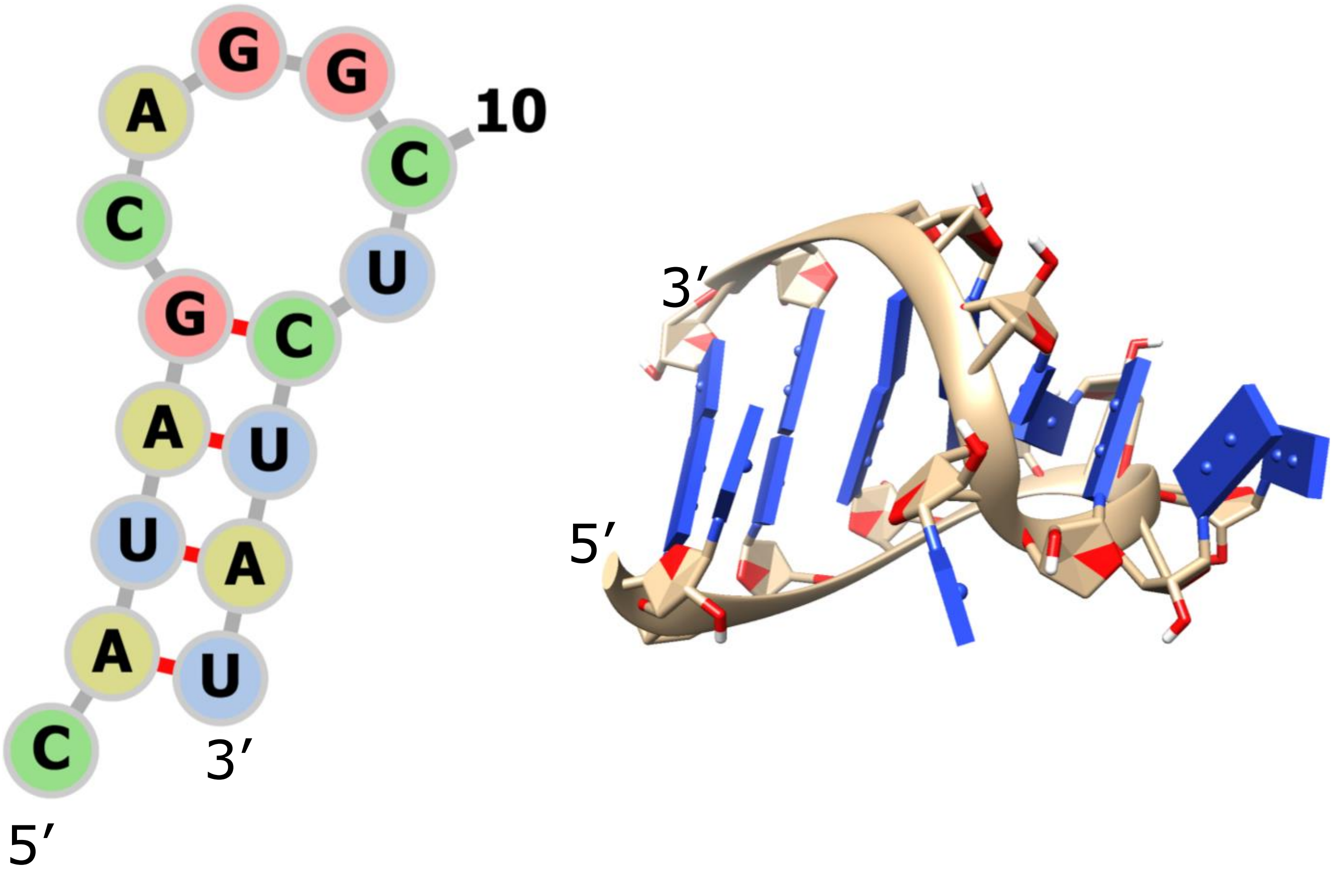

7I$_{ctrl}$: tRNA from the bacterium “Derad”,
for Gly, <u>GCC</u>, shuffled, MFE = -0.29

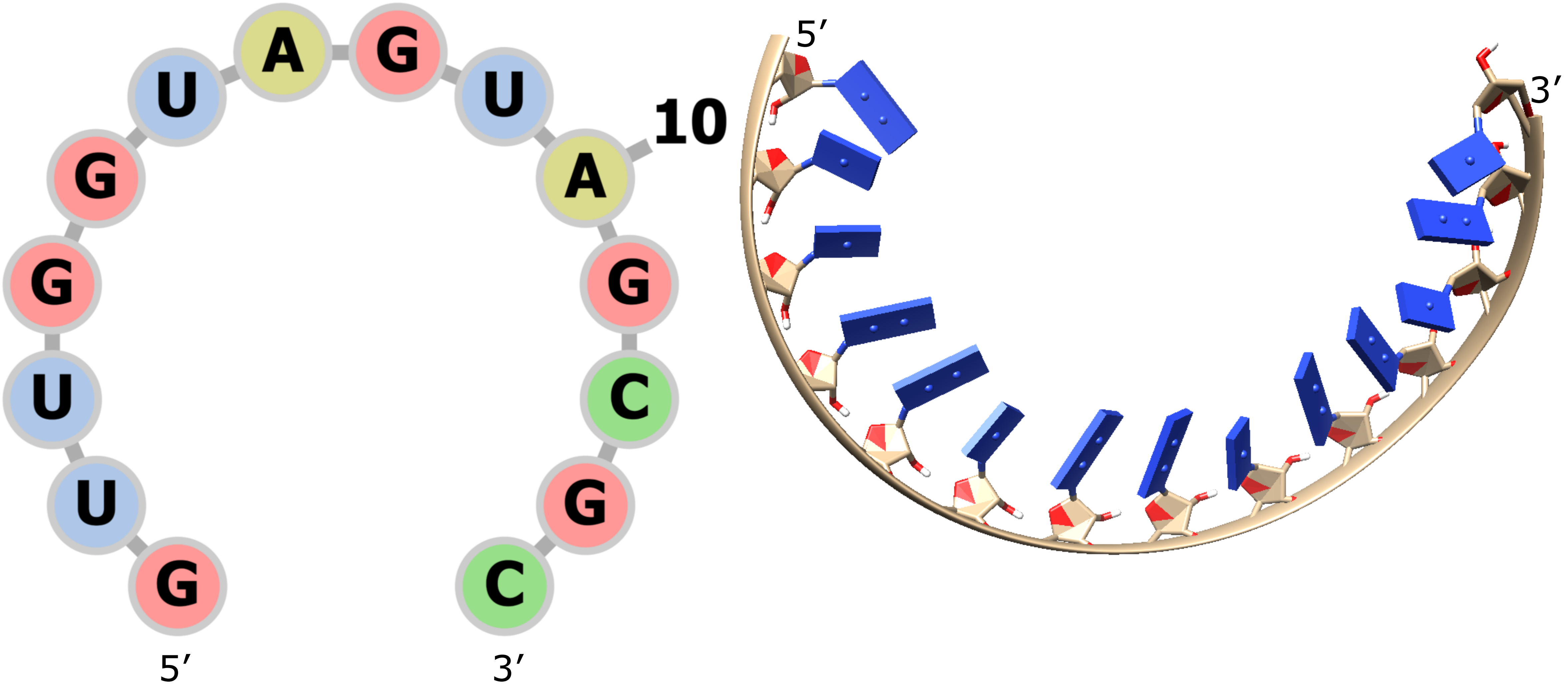

7J$_{bio}$: tRNA from the bacterium "SagA", for Asn, GUU, MFE = 0.0

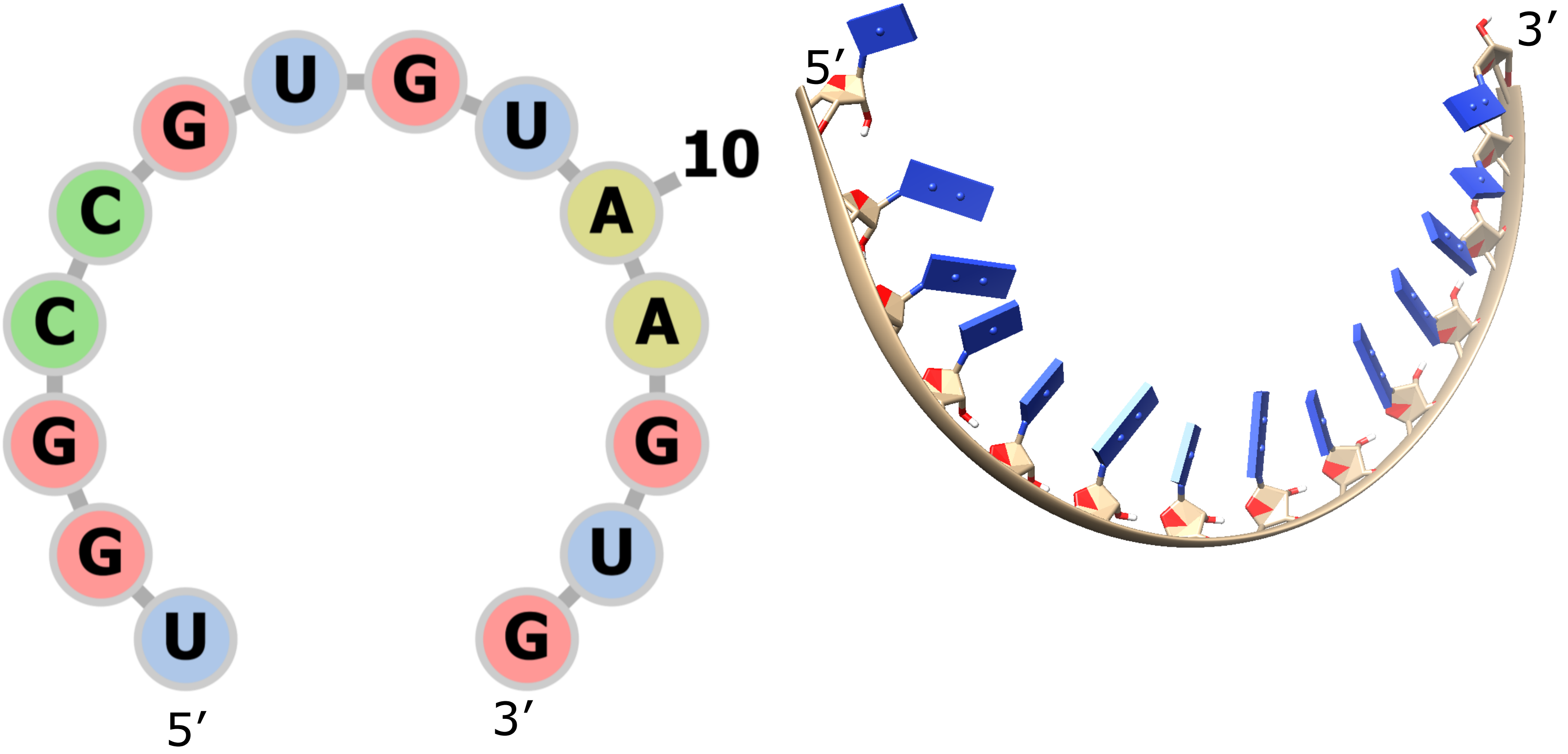

7J$_{ctrl}$: tRNA from the bacterium “SagA”, for Asn, GUU, shuffled, MFE = 0.0

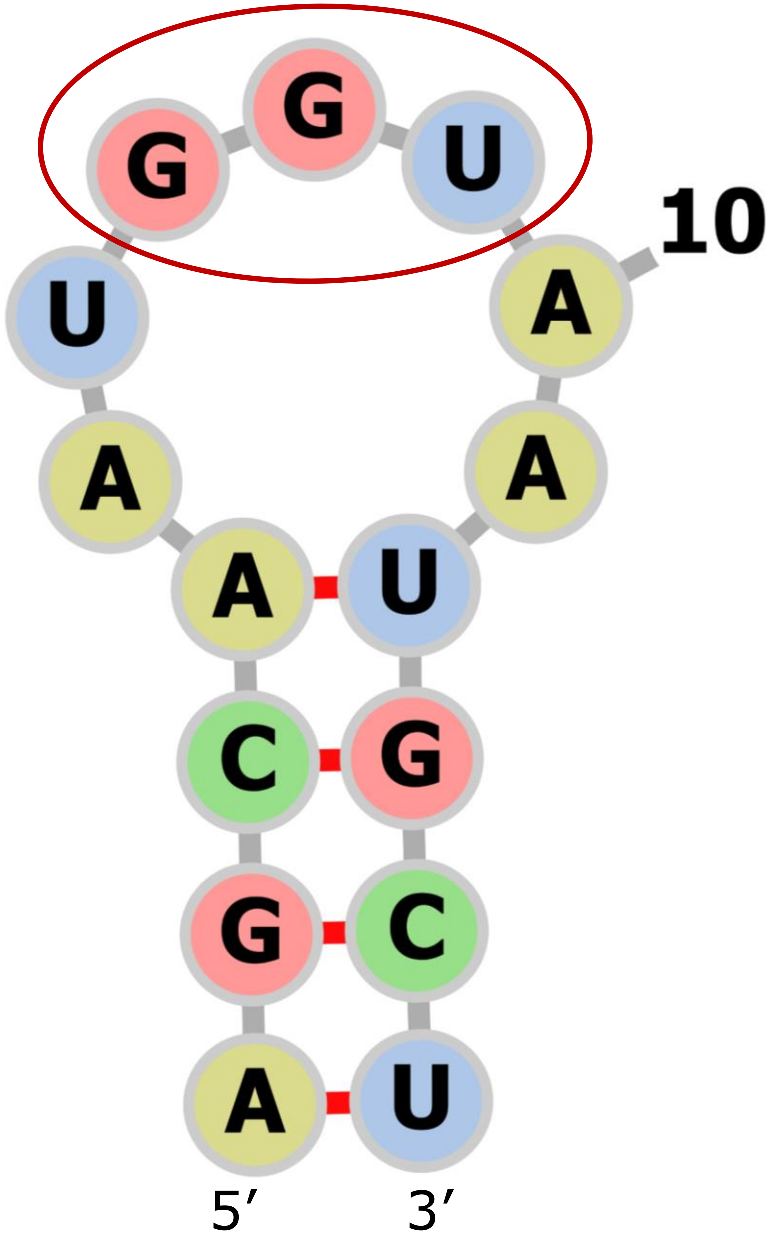

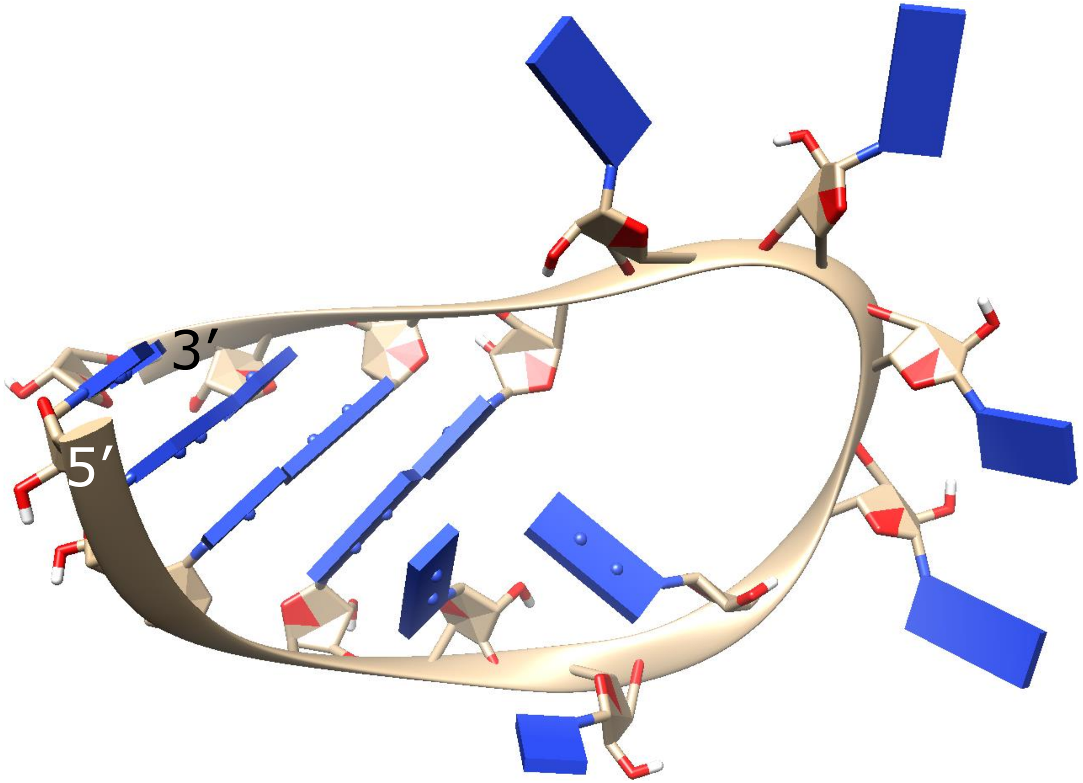

7K$_{bio}$: tRNA from the bacterium "SagA", for Thr, <u>GGU</u>, MFE = -2.40

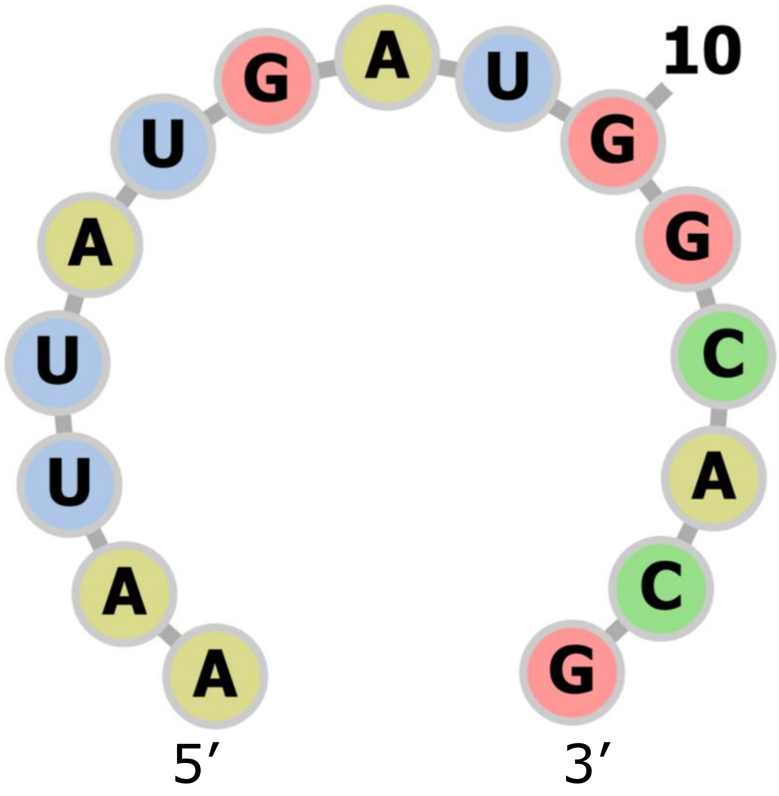

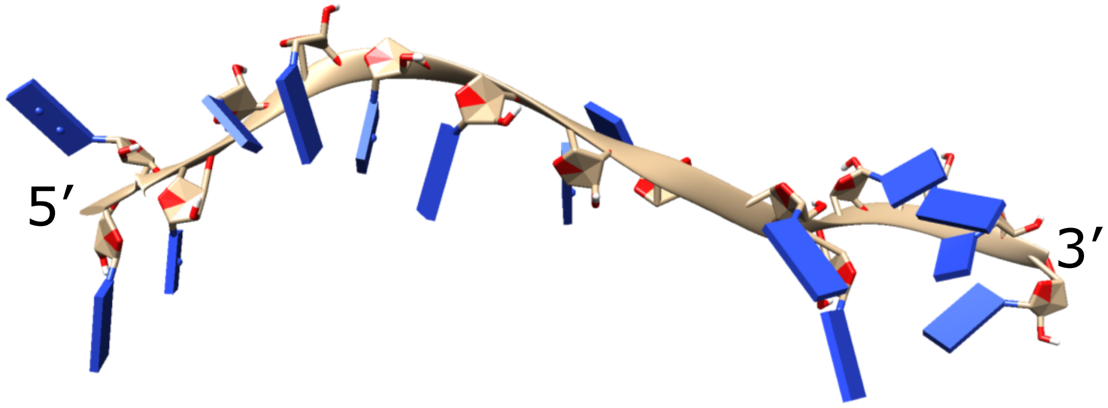

7K$_{ctrl}$: tRNA from the bacterium "SagA",
for Thr, GGU, shuffled, MFE = 0.0

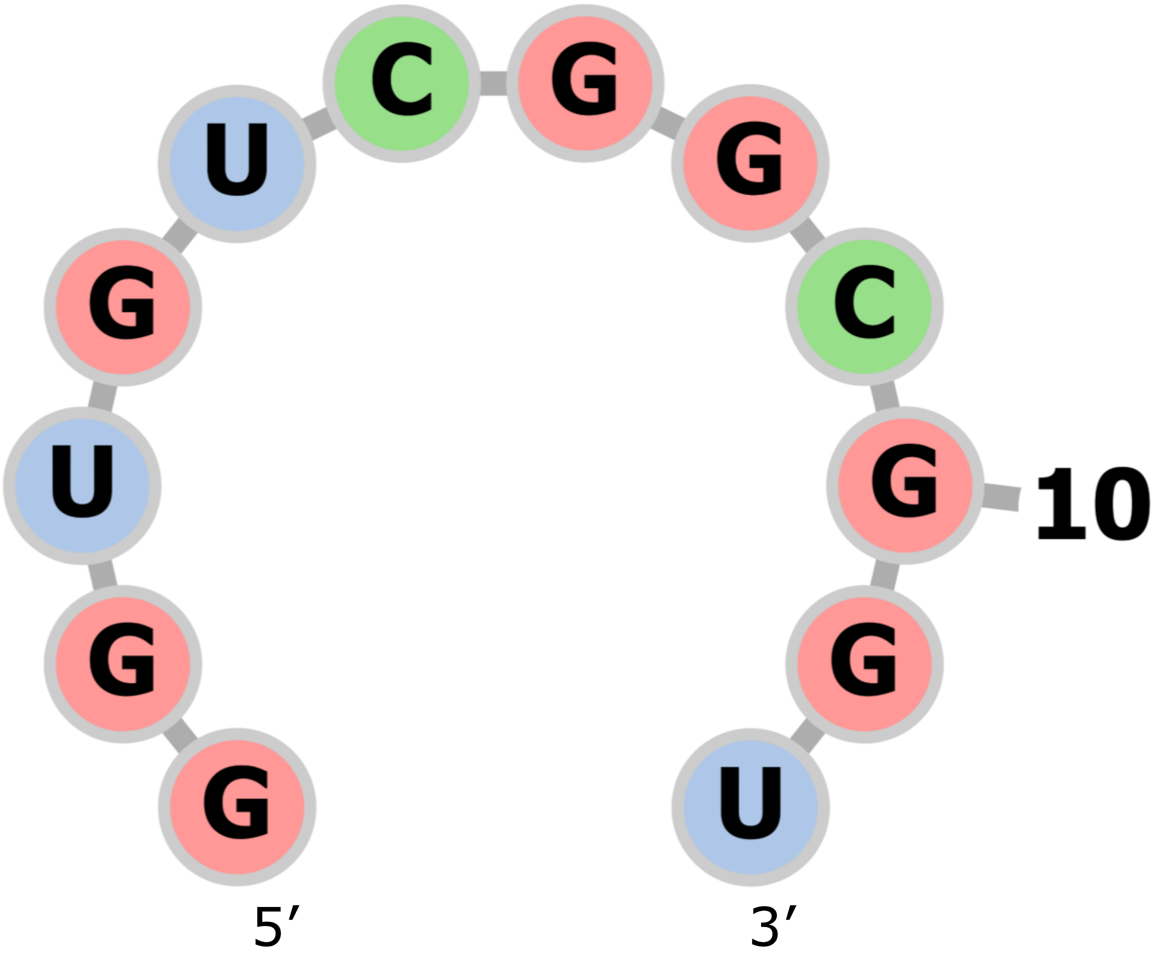

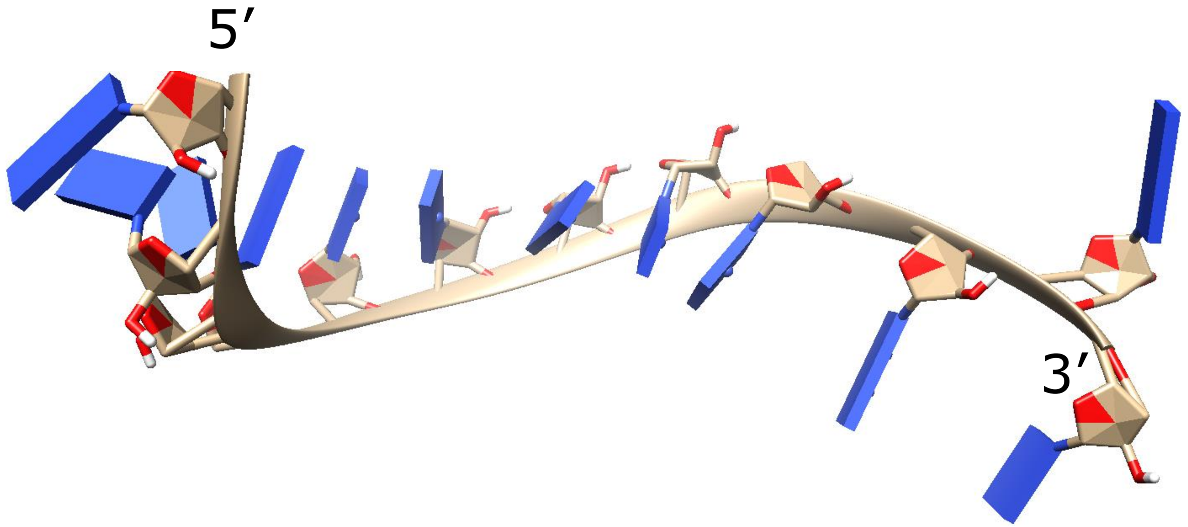

7L$_{bio}$: tRNA from the bacterium “Thmar”,
for Phe, GAA, MFE = 0.0

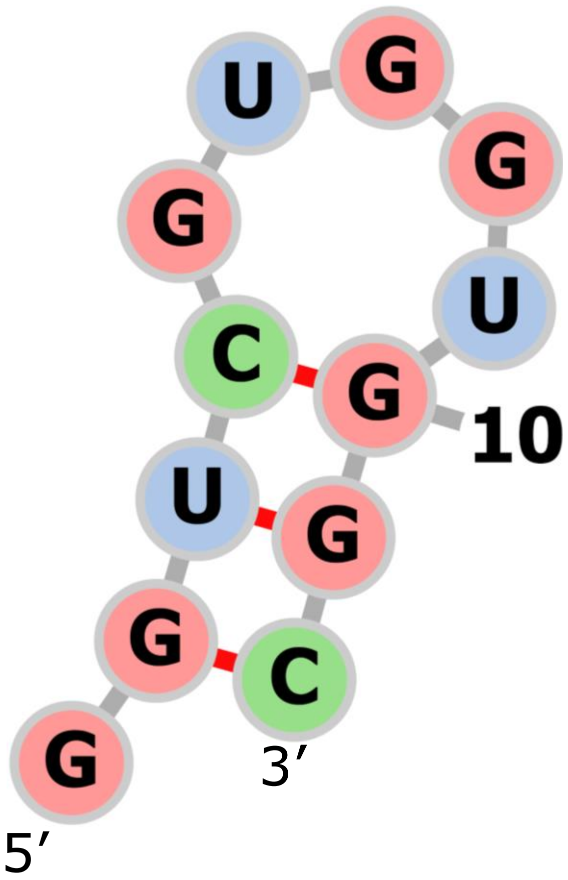

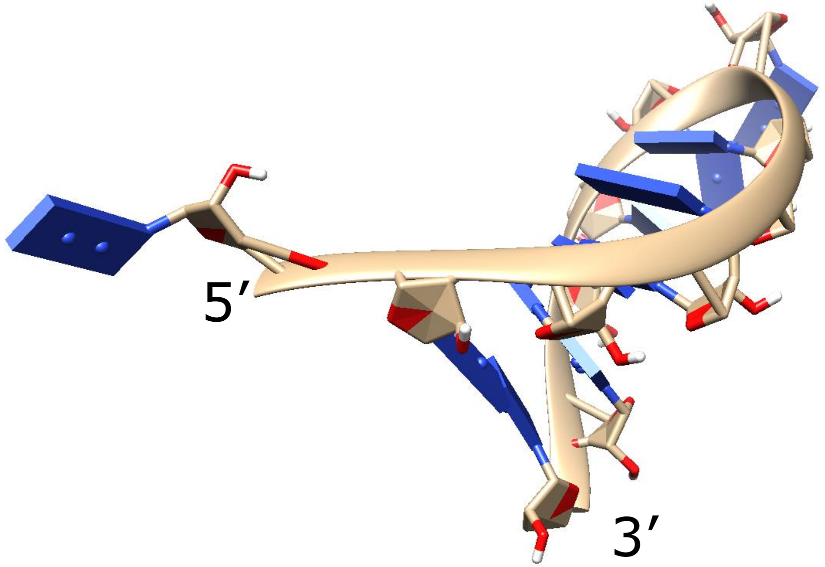

7L$_{ctrl}$: tRNA from the bacterium “Thmar”, for Phe, GAA, shuffled, MFE = -0.30
